# Reprogramming neural-tumor crosstalk: emerging therapeutic dimensions and targeting strategies

**DOI:** 10.1186/s40779-025-00661-9

**Published:** 2025-10-30

**Authors:** Qian-Qian Liu, Zi-Kai Dong, Yong-Fei Wang, Wei-Lin Jin

**Affiliations:** 1 Institute of Cancer Neuroscience, Medical Frontier Innovation Research Center, the First Hospital of Lanzhou University, the First Clinical Medical College of Lanzhou University, Lanzhou, 730000 China; 2 State Key Laboratory of Experimental Hematology, National Clinical Research Center for Blood Diseases, Haihe Laboratory of Cell Ecosystem, Tianjin Key Laboratory of Gene Therapy for Blood Diseases, CAMS Key Laboratory of Gene Therapy for Blood Diseases, Institute of Hematology and Blood Diseases Hospital, Chinese Academy of Medical Sciences and Peking Union Medical College, Tianjin, 300020 China

**Keywords:** Bioelectricity, Cancer neuroscience, Drug repurposing, Neurotransmitters, Neurotrophic factors, Targeted therapy

## Abstract

Cancer neuroscience, an emerging convergent discipline, offers novel insights into the dynamic interplay between the nervous system and cancer progression. Bidirectional signaling between the nervous system and tumors, particularly within the innervated tumor microenvironment (TME), modulates key cancer hallmarks, including proliferation, immune evasion, angiogenesis, and metastasis. Neural ablation shows heterogeneous outcomes depending on nerve subtype and tumor context, underscoring the importance of nerve-type-specific and context-dependent therapeutic approaches. These mechanistic advances are catalyzing novel therapeutic strategies that target neural-TME interactions through the integration of neuroscience and oncology. Here, we highlight recent progress in cancer neuroscience and propose revised therapeutic frameworks aimed at the neuro-innervated TME. These strategies employ interdisciplinary approaches, such as drug repurposing [β-adrenergic receptor (β-AR) blockers, antipsychotics, antidepressants], and nanotechnology-enabled targeted delivery. Both preclinical and clinical data support the potential of neural-targeted therapies to improve precision, circumvent drug resistance, and enhance clinical outcomes. By bridging neuroscience and oncology, this framework delineates a translational pathway for harnessing neural-tumor crosstalk, presenting a promising avenue for advancing cancer therapeutics and improving patient care.

## Background

Tumors are the second leading cause of death worldwide and impose a substantial economic burden [1]. The nervous system plays a pivotal role in regulating physiological processes (e.g., tissue development, organogenesis, homeostasis, and regeneration) and pathological processes (e.g., tumorigenesis, neurodegeneration, and chronic pain) [2, 3]. Given that cancers often exploit these very pathways during initiation, proliferation, and progression, the nervous system is likely implicated in multiple facets of tumor biology. Conversely, cancer and its therapies can impair neural function, establishing a pathological feedback loop that accelerates tumor progression [4, 5]. This bidirectional relationship forms the basis of an emerging discipline termed cancer neuroscience, which seeks to elucidate the intricate crosstalk between the nervous system and cancer [6].

**Fig. 1:**
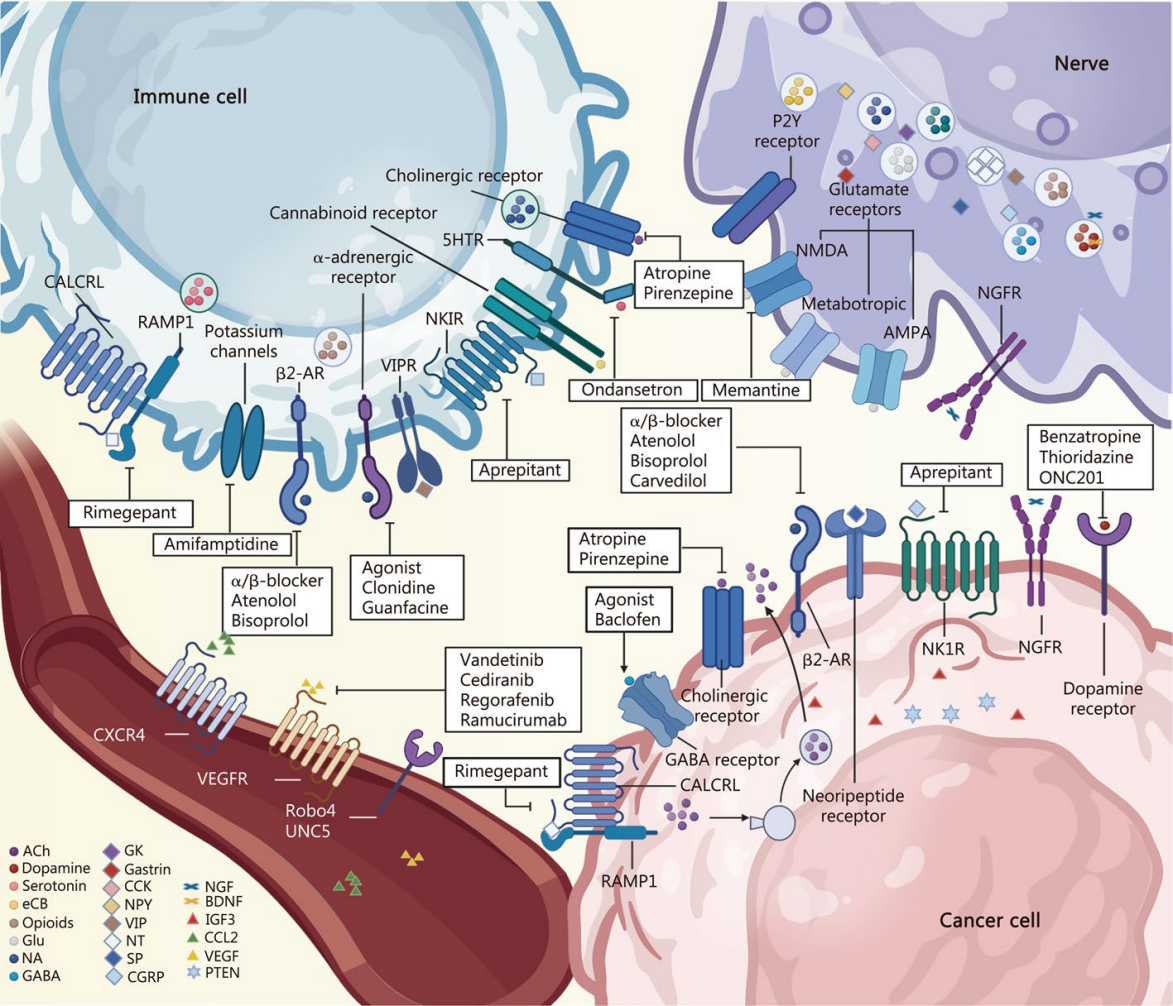
Interactions within the tumor niche. The nervous system, immune system, and blood vessels play a key role in tumor progression, invasion and migration, immune escape, and treatment resistance. Neurons release various signaling molecules, such as neurotransmitters and neuropeptides, which bind to receptors on the surfaces of immune cells and cancer cells, regulating immune function and tumor progression. Immune cells modulate their activity by expressing receptors for neural and tumor-derived signals and secrete cytokines that enhance neuronal excitability, stimulate angiogenesis, and promote cancer cell invasiveness. Cancer cells integrate signals from neurons and immune cells through specific receptors, promoting their proliferation and invasion, while also secreting factors that sustain angiogenesis, activate neurons, and modulate immune responses. Vessels, through their endothelial cells, respond to angiogenic signals, such as VEGF, secreted by cancer cells and immune cells, promoting metastatic spread and providing the necessary structural support for tumor progression. These interactions collectively form a positive feedback loop, creating a more permissive tumor microenvironment that promotes immune evasion and therapeutic resistance. This figure was created using BioRender (https://biorender.com/). ACh acetylcholine, GABA γ-aminobutyric acid, CGRP calcitonin gene-related peptide, CCK cholecystokinin, eCB endocannabinoids, CALCRL calcitonin receptor-like, RAMP1 receptor activity-modifying protein 1, 5HTR 5-hydroxytryptamine receptor, VEGF vascular endothelial growth factor, VEGFR vascular endothelial growth factor receptor, CCL2 C-C motif chemokine ligand 2, CXCR4 C-X-C motif chemokine receptor 4, β2-AR β2-adrenergic receptors, VIPR vasoactive intestinal peptide receptor, NKIR(NK1R) neurokinin 1 receptor, Robo4 roundabout guidance receptor 4, UNC5 uncoordinated-5 homolog, P2Y P2Y purinoceptor, NMDA N-methyl-D-aspartate, AMPA α-amino-3-hydroxy-5-methyl-4-isoxazolepropionic acid, NGF nerve growth factor, NGFR nerve growth factor receptor, RAMP1 receptor activity-modifying protein 1, Glu glutamic acid, NE noradrenaline, GK glucokinase, NPY neuropeptide Y, VIP vasoactive intestinal peptide, SP substance P, BDNF brain-derived neurotrophic factor, IGF3 insulin-like growth factor 3, PTEN phosphatase and tensin homolog, ONC201 dordaviprone, NT neurotensin

Tumors can actively recruit nerves into the tumor microenvironment (TME), offering a new lens through which to view neural-tumor interactions. As precancerous lesions progress, nerve density within the TME increases markedly [7, 8]. Neural signals, through ligand-receptor interactions, regulate the expression of neurotransmitters and neuropeptides on the surfaces of cancer cells [9, 10]. Moreover, the ability of cancer cells to autonomously synthesize neuromodulatory molecules suggests a potential for autocrine regulation of proliferation. Notably, neurons may form direct functional synapses with cancer cells, further enhancing regulatory control [9, 10]. Within the TME, a complex signaling network shapes interactions among nerves, immune cells, and cancer cells. For instance, vagus nerve (VN) activation induces acetylcholine (Ach) release, which suppresses macrophage activity, modulates the immune landscape, and promotes tumor-associated inflammation, a process that may contribute to resistance against targeted therapies [9]. Innervation may also directly promote metastasis, as tumor-associated nerves extend toward the central nervous system (CNS) and potentially activate metastatic precursors [11, 12]. These insights reveal therapeutic opportunities, supported by growing preclinical and clinical evidence linking neural activity to tumor progression (Fig. 1).

Over the past decade, cross-disciplinary collaboration between oncology and engineering has surged, propelled by advances in biomanufacturing, nanomedicine, and materials science [13, 14]. For example, nanomaterials with large surface-to-volume ratios and customizable surfaces enable stable, targeted drug delivery [13]. Such collaborative efforts have not only advanced our understanding of neural influences within tumors but also provided innovative strategies for targeting the neuro-innervated TME. This review synthesizes current evidence to establish a comprehensive clinical and translational framework for targeting neural-tumor interactions in cancer treatment. We first investigated the interactions between neurons and tumors, revealing how cancer cells recruit neurons into the TME and how neuronal signals, in turn, shape tumor characteristics and behavior. Building on these insights, we propose 9 conceptual frameworks for targeting the neuro-innervated TME, along with strategies to achieve precision-targeted therapy. This integrated approach, spanning from fundamental mechanistic insights to precision-targeted strategies, seeks to enhance therapeutic accuracy and efficacy, ultimately advancing the development of more effective, mechanism-based cancer treatments.

## Neural-tumor interactions

Neuronal activity governs organ development, systemic homeostasis, plasticity, and regeneration. The cellular and molecular mechanisms underlying activity-dependent physiological regulation in healthy states may offer insights into how the nervous system similarly modulates tumor biology. The formation of functional neural circuits requires axonal growth and guidance, synaptogenesis, and the refinement of neuronal connectivity. Organogenesis also depends on proper neural innervation. For example, parasympathetic input is essential for salivary gland development [15]. The nervous system further contributes to tissue regeneration, as denervation impairs regenerative capacity in adult organs, whereas restoration of cholinergic signaling enhances epithelial regeneration in salivary tissue [16]. During development and repair, blood vessels and nerves rely on shared signals and mechanisms to differentiate, extend, and navigate toward their targets [17].

Notably, neural influence begins not only after tumor onset but is evident during early precancerous stages. Inflammation and neuronal damage in both the peripheral nervous system (PNS) and CNS arise as early as pancreatic intraepithelial neoplasia stage 2. At this point, acinar-derived cells frequently invade sensory neurons and migrate along the spinal cord toward lower thoracic and upper lumbar regions. Neuronal ablation in mouse models significantly delays pancreatic intraepithelial neoplasia formation and prolongs survival [18]. During the initiation of precancerous lesions, adrenergic signaling may facilitate the malignant transformation of mammary epithelial cells. It also modulates the behavior of other TME components, such as macrophages and fibroblasts, promoting pro-tumorigenic phenotypes and altering adipocyte function. Additionally, adrenergic signaling enhances angiogenesis and lymphangiogenesis, potentially establishing a precancerous microenvironment conducive to tumor progression [19]. This underscores the compelling significance of neural-tumor interactions: the “noncanonical” roles of neural activity, including morphogenesis, activation of developmental programs to support growth, invasion, and colonization, as well as orchestration of vascular and immune niches and regenerative capacity, are deeply relevant to cancer biology. While this study focuses on the functional contributions of the nervous system during cancer progression, its role in precancerous transformation is only briefly addressed. Systematic investigation into neural mechanisms during precancerous stages remains sparse and merits dedicated exploration as a distinct research avenue. This section highlights the dynamic remodeling of neural elements within the TME and their integrative roles in modulating cancer hallmarks.

### Nerves in TME

Tumor progression is closely associated with innervation [20–23]. Several mechanisms have been proposed to explain how tumors recruit nerves, including axonogenesis, neural reprogramming, neurogenesis, and perineural invasion (PNI) [24]. In this section, we synthesize recent advances in understanding the origins and recruitment of neuronal tissue within tumors.

#### Axonogenesis

**Fig. 2:**
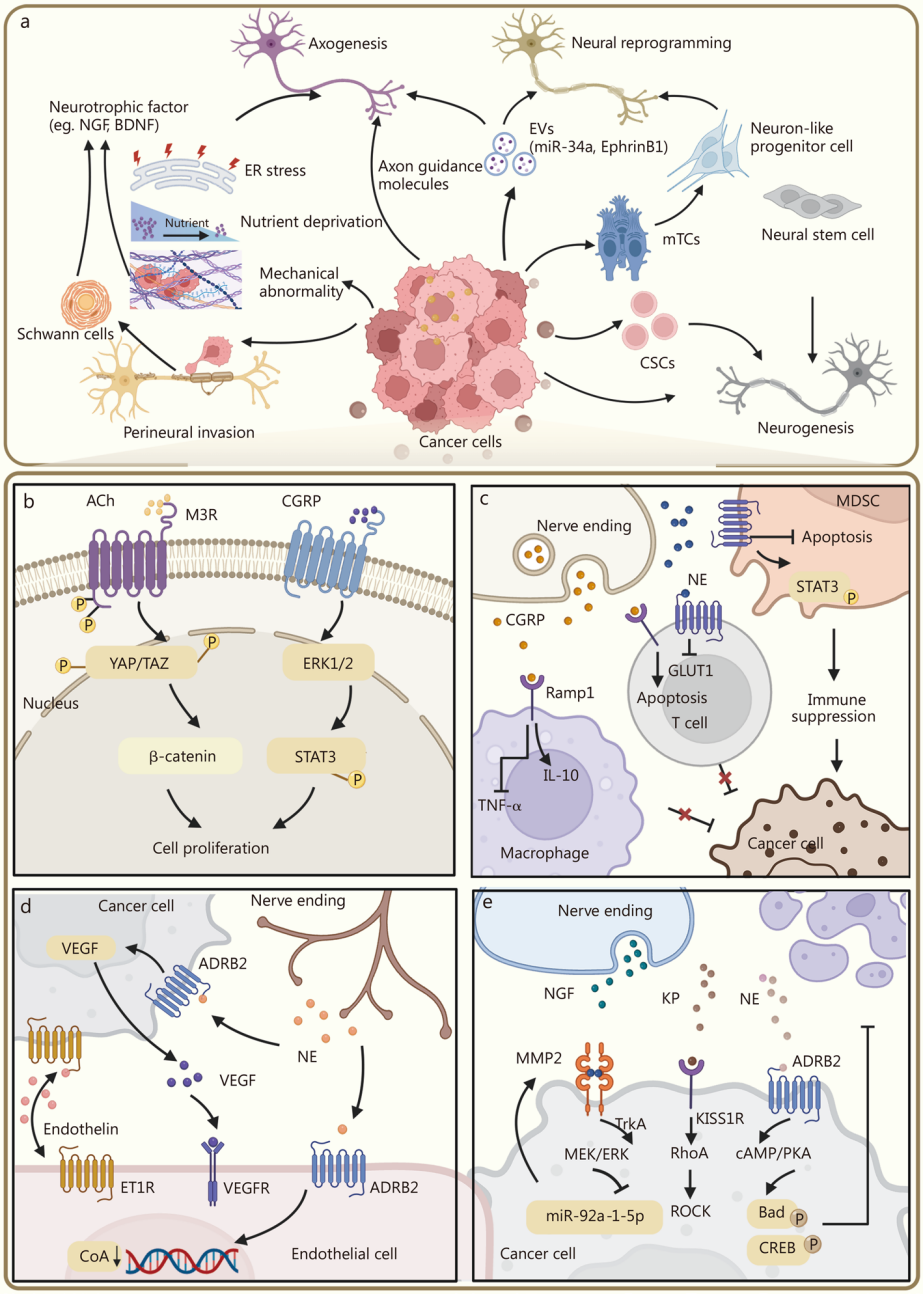
The interaction between nerves and tumors in the tumor microenvironment. This figure illustrates some representative mechanisms of nerve-tumor interactions in the tumor microenvironment. **a** There are various sources of nerves in tumors, such as neural stem cell differentiation, tumor cells inducing nerve cell reprogramming, cancer cells secreting molecules to promote axon growth and perineuronal invasion, etc. **b** ACh activates the β-catenin signaling pathway through M3R, and CGRP promotes tumor growth and proliferation through ERK1/2/STAT3 signaling. **c** CGRP can induce immunosuppression by promoting immune cell production of inflammatory cytokines, inducing T cell depletion, and inhibiting MDSC apoptosis. **d** NE promotes tumor angiogenesis by promoting the secretion of VEGF and decreasing the expression of CoA in endothelial cells. **e** NGF, KP, and NE promote tumor invasion and metastasis through MEK/ERK, RhoA/ROCK, and cAMP/PKA signaling pathways, respectively. This figure was created using BioRender (https://biorender.com/). ACh acetylcholine, M3R M3 muscarinic acetylcholine receptor, CGRP calcitonin gene-related peptide, ERK1/2 extracellular signal-regulated kinase 1/2, 2-MAPK 2-mitogen-activated protein kinase, MDSCs myeloid-derived suppressor cells, NE norepinephrine, VEGF vascular endothelial growth factor, CoA coenzyme A, NGF nerve growth factor, KP kisspeptin, MEK mitogen-activated protein kinase kinase, ERK extracellular signal-regulated kinase, RhoA ras homolog family member A, ROCK rho-associated coiled-coil containing protein kinase, cAMP cyclic adenosine monophosphate, PKA protein kinase A, BDNF brain-derived neurotrophic factor, ER endoplasmic reticulum, EVs extracellular vesicles, CSCs cancer stem cells, mTCs mature tumor cells, YAP/TAZ Yes-associated protein/transcriptional co-activator with PDZ-binding motif, STAT3 signal transducer and activator of transcription 3, GLUT1 glucose transporter 1, IL-10 interleukin-10, TNF-α tumor necrosis factor-α, ADRB2 adrenoceptor beta 2, ET1R endothelin-1 receptor, VEGFR vascular endothelial growth factor receptor, Bad Bcl-2-associated death promoter, CREB cAMP response element-binding protein, NE norepinephrine

Malignant tumors secrete axon guidance molecules and growth factors that promote nerve fiber extension. Members of the semaphorin family play a central regulatory role in this process: semaphorin 4F (Sema4F) induces nerve terminal sprouting and increases axon length by nearly threefold, while Sema3D facilitates pancreatic nerve invasion via binding to the plexin D1 receptor [25]. Various tumor microenvironmental stressors, such as endoplasmic reticulum (ER) stress, nutrient deprivation, and mechanical abnormality, can synergistically regulate neurotrophic factors, including nerve growth factor (NGF), brain-derived neurotrophic factor (BDNF), precursor for BDNF (proBDNF), and other related factors, thereby activating neural growth pathways [26–28]. Recent findings show that β-III tubulin-positive exosomes derived from cervical cancer tissues can induce neurogenesis in adjacent cervical stromal regions [29]. Moreover, CD9⁺ exosomes secreted by human papillomavirus-positive tumors promote axonogenesis and tumor nerve infiltration by carrying EphrinB1, whereas exosomes lacking EphrinB1 fail to elicit this effect [30] (Fig. 2a).

#### Neural reprogramming

Tumors can induce ectopic neuron generation through cell fate remodeling. During the progression of pancreatic ductal adenocarcinoma (PDAC), metaplastic tuft cells, abnormally appearing at precancerous sites, exhibit neuroendocrine transformation potential. Lineage tracing experiments have confirmed that these tuft cells can transdifferentiate into neural-like progenitor cells, contributing to the emergence of a neuroendocrine phenotype in PDAC [31–35]. In parallel, intrinsic nerve fibers within tumors may also undergo phenotypic reprogramming. For example, extracellular vesicles (EVs) secreted by p53-deficient cells in head and neck cancers can reprogram sensory nerves into noradrenergic nerves [36]. These findings indicate that the neuronal populations within tumors are likely of diverse origins, with their cellular composition shaped by tumor-specific factors and histological context.

#### Neurogenesis

In situ neurogenesis can arise from both neural progenitor cells and tumor stem cells. Elevated expression of neuroprecursor markers such as nestin and doublecortin within the tumor parenchyma suggests that the TME supports neuronal maturation [37, 38]. In transgenic models of prostate cancer (PCa), doublecortin-positive neural progenitors from the subventricular zone have been shown to traverse the blood-brain barrier and infiltrate tumors, with their density correlating positively with tumor invasiveness and recurrence risk [39, 40]. Notably, these progenitors possess the capacity to differentiate into noradrenergic neurons and may directly contribute to malignant progression. Further studies are needed to determine how previously uninnervated normal tissues establish new neural connections during tumorigenesis. In addition to recruiting peripheral neural progenitors, cancer stem cells (CSCs) themselves can undergo neurodifferentiation. Sympathetic nerve marker tyrosine hydroxylase has been detected in colorectal and gastric CSCs, while differentiated gastric CSCs have also been found to express the parasympathetic marker vesicular Ach transporter [41], highlighting the neurogenic potential of CSCs and suggesting a novel therapeutic avenue through targeting neurodifferentiation processes (Fig. 2a).

#### PNI

PNI, a hallmark of neural-tumor interaction, is characterized by cancer cells infiltrating the nerve sheath and establishing intimate contact with axons and Schwann cells (SCs), thereby facilitating local tumor spread and distant metastasis [42]. This process is mediated by the synergistic actions of neurotrophic signaling pathways and chemokine networks [43–46]. SCs, the principal glial cells of the PNS, ensheath axons and fulfill diverse roles, including rapid signal conduction, neurotrophic support, extracellular matrix production, neurogenesis, and neural repair [47, 48]. Upon nerve injury or tumor invasion, SCs can undergo partial dedifferentiation into a demyelinating, repair-associated phenotype [49]. These repair SCs secrete neurotrophic factors and pro-inflammatory cytokines, remodel the local microenvironment, and recruit macrophages to coordinate axonal regrowth and tissue repair [50, 51]. Furthermore, SCs undergo adaptive molecular reprogramming that promotes protective, anti-tumor neuronal responses [52]. In this context, dedifferentiated SCs play a pivotal role in shaping a permissive neural microenvironment that facilitates the progression of PC.

### The nervous system reshapes cancer hallmarks

The interplay between neurons and tumors is mediated through multiple secretory pathways by which bioactive molecules dynamically remodel tumor biology [53]. These interactions can be broadly classified into 3 modes. (1) Autocrine signaling: certain tumor cells, particularly in neuroendocrine tumors or neuroendocrine carcinomas, secrete neuropeptides that act in a paracrine-like fashion, indicating a self-stimulatory mechanism that promotes tumor progression. (2) Endocrine signaling: systemic neuroactive molecules travel via the bloodstream to reach the TME, where they modulate cellular characteristics and functions at distant sites. (3) Paracrine signaling: neuroactive substances are predominantly released by neighboring neurons and bind to specific receptors on tumor cells, thereby altering tumor behavior. Importantly, these neuroactive signals also act on non-malignant components of the TME, including immune and stromal cells [53, 54]. Collectively, neural innervation regulates diverse tumor traits through both direct interactions with cancer cells and indirect modulation of the surrounding microenvironment.

#### Cancer proliferation

Uncontrolled proliferation is a hallmark of cancer, and neural signaling contributes to aberrant tumor growth through multiple mechanisms. Cholinergic stimulation of the gastric epithelium induces the expression of NGF, and NGF overexpression expands enteric innervation and promotes carcinogenesis. Blocking the NGF/tropomyosin receptor kinase (Trk) signaling pathway suppresses epithelial proliferation by impairing Yes-associated protein function through the M3 muscarinic acetylcholine receptor (M3R) [55]. Calcitonin gene-related peptide (CGRP) has also been shown to markedly promote tumor growth through extracellular signal-regulated kinases (ERKs)/signal transducer and activator of transcription 3 (STAT3) signaling pathways [56, 57] (Fig. 2b). Additionally, the neuron-specific protein neuroligin-3 (NLGN3), once cleaved by a disintegrin and metalloproteinase domain-containing protein 10 (ADAM10), drives malignant glioma proliferation [58, 59]. Together, these findings demonstrate that, beyond classical neurotransmitters, neural signals orchestrate tumor cell cycle regulation via context-specific protein networks within the TME.

#### Cancer immunity

Both neurons and glial cells within the nervous system modulate immune cells in the TME [60], thereby shaping the immune landscape and influencing therapeutic outcomes [61–63]. Neural regulation of immune responses and cancer cell behavior occurs through both direct and indirect mechanisms [64, 65]. Sympathetic β-adrenergic signaling suppresses interferon-γ production by T cells and enhances the survival of myeloid-derived suppressor cells (MDSCs) [66]. CGRP drives macrophage polarization and accelerates CD8^+^ T cell depletion (Fig. 2c), whereas substance P (SP) supports T cell survival and promotes the secretion of inflammatory cytokines [67]. Tumor-associated nerves have been shown to express immune checkpoint molecules such as programmed death-1 (PD-1) and programmed death-ligand 1 (PD-L1), directly inhibiting T cell activity. Indirect modulation involves the nervous system’s systemic control of physiological processes that impact the TME [64, 68]. For example, the sympathetic nervous system can regulate hematopoiesis within the bone marrow niche, thereby influencing immune cell output and facilitating tumor metastasis [69, 70]. The effects of specific nerve types on immunity and tumor progression are likely complex and context-dependent. For example, parasympathetic and sensory nerves exhibit opposing roles in pancreatic cancer. Parasympathetic nerves appear to exert antitumor effects in PDAC. In mouse models of PDAC, parasympathetic denervation significantly increased tumor necrosis factor (TNF) levels and promoted the recruitment of tumor-associated macrophages (TAMs) [71, 72], thereby enhancing malignant epithelial proliferation and increasing tumor incidence [72, 73]. In contrast, sensory nerves play a pro-tumorigenic role, particularly in the setting of chronic pancreatic inflammation, a well-established risk factor for PDAC. In PDAC models, upregulation of transient receptor potential vanilloid 1 (TRPV1) and transient receptor potential ankyrin 1 (TRPA1) channels in sensory neurons promotes neurogenic inflammation and accelerates tumorigenesis [74, 75]. Notably, ablation of sensory neurons in murine models of pancreatitis suppresses inflammation and delays cancer initiation [18].

#### Cancer angiogenesis

Tumor cells depend on the vascular system for oxygen and nutrient delivery, as well as for the clearance of metabolic waste. Notably, nerve fibers and blood vessels frequently co-localize within neurovascular bundles, an anatomical arrangement that implies functional crosstalk between the nervous and vascular systems [76, 77]. Norepinephrine (NE) released from sympathetic nerve terminals activates β2-adrenergic receptors (β2-AR) on endothelial cells, suppressing oxidative phosphorylation and promoting aerobic glycolysis, a metabolic shift essential for angiogenesis [66]. In PCa models, β2-AR-deficient endothelial cells exhibit enhanced oxidative phosphorylation, increased glucose uptake, and elevated expression of mitochondrial genes such as cytochrome c oxidase assembly factor 6 (CoA6) [78]. Moreover, chronic stress has been shown to upregulate vascular endothelial growth factor (VEGF), markedly increasing *VEGF* mRNA and protein levels within tumor tissues [79]. These findings underscore the complexity of neurovascular signaling networks and their contributions to tumor angiogenesis, providing a mechanistic rationale for the development of anti-angiogenic therapies targeting neural regulatory pathways (Fig. 2d).

#### Cancer invasion and metastasis

Tumor invasion and metastasis are leading causes of cancer-related mortality and remain major obstacles to effective therapy [80]. Metastasis requires that tumor cells at the primary site to degrade the basement membrane, enter the circulatory system, and extravasate into distant tissues to establish secondary tumors [80]. Neural signals play key roles in orchestrating these processes. Kisspeptin (KP), a neuropeptide, typically signals via its receptor, G protein-coupled receptor 54. KP can suppress metastasis by regulating matrix metalloproteinase (MMP) expression through the mitogen-activated protein kinase (MAPK)/ERK pathway or by inhibiting cytoskeletal remodeling via suppression of the Ras homolog family member A/Rho-associated coiled-coil containing protein kinase signaling axis [81, 82] (Fig. 2e). However, in certain contexts, such as triple-negative breast cancer (TNBC), KP exerts a pro-metastatic effect by promoting cell invasion through the activation of cortactin, cofilin, and membrane-type 1 MMP [83]. These context-dependent effects underscore the complexity of neuropeptide signaling in cancer metastasis. Beyond molecular mechanisms, neural influences on metastasis can also be observed at a macroscopic level, often with divergent effects depending on the nerve type. In PCa research, parasympathetic cholinergic fibers have been shown to enhance tumor invasiveness in two mouse models via activation of stromal type 1 muscarinic Ach receptors [14, 84]. In contrast, sensory nerve inactivation may paradoxically promote tumor aggressiveness. Cardiac metastasis, tumor cells isolated after capsaicin-induced sensory denervation exhibited increased invasive and metastatic potential compared to controls [85, 86]. However, caution is warranted in extrapolating these findings to humans, as patterns of tumor innervation may differ substantially between mouse models and clinical settings.

#### Other hallmarks

Metabolic reprogramming is a hallmark of cancer, enabling tumor cells to sustain growth under nutrient-limited conditions. Aerobic glycolysis allows cancer cells to outcompete neighboring normal cells for glucose uptake [87]. In glioblastoma (GBM), tumor cells autonomously synthesize dopamine, activating dopamine receptor D2 and significantly increasing glucose uptake and glycolytic flux, establishing a unique, dopamine-driven metabolic program [88]. The Warburg effect refers to the phenomenon whereby cancer cells preferentially utilize glycolysis for ATP production even under conditions of sufficient oxygen availability. NE can amplify the Warburg effect, particularly in the context of comorbid factors such as hypertension and obesity [89]. NE contributes to immunosuppression by inhibiting pancreatic insulin secretion via β2-AR signaling, which in turn suppresses T cell glycolysis and oxidative phosphorylation, thereby promoting immune metabolic exhaustion [90, 91]. This also indicates that neural signals regulate cancer progression through multi-dimensional mechanisms, influencing not a single marker but the entire trajectory of malignant evolution. The influence of neural signaling extends beyond metabolism, affecting cancer hallmarks such as genomic instability and inflammation within the TME [92, 93]. For example, catecholamine exposure in ovarian cancer cells has been shown to induce double-stranded DNA breaks [92]. These processes underscore the central role of neural-tumor interactions within the tumor ecosystem and point out that targeting neural regulatory networks may break through the limitations of traditional therapies by blocking key pathways.

Synapses are the fundamental units of neural communication, and interactions between neurons and brain tumor cells can occur via two distinct mechanisms [41]: 1) bona fide chemical synapses, as seen in GBM, and 2) pseudo-tripartite synaptic structures, such as those observed in breast-to-brain metastases (B2BM) [94]. In gliomas, functional neuron-tumor chemical synapses have been identified at cellular interfaces, displaying electrophysiological characteristics consistent with α-amino-3-hydroxy-5-methyl-4-isoxazolepropionic acid receptor (AMPAR)-mediated signaling [94, 95]. These synapses do not form through direct neuronal connections but rely on tumor microtubes (TMs) within glioma tissue, enabling signal propagation via gap junctions [94, 96]. In parallel, studies of B2BM have shown that activation of glutamate ligand-gated channels, particularly N-methyl-D-aspartate receptors (NMDAR), facilitates the brain colonization of breast cancer cells [97]. B2BM cells express adhesion molecules such as postsynaptic density protein 95, which support the formation of synapse-like junctions between non-neuronal cells and axons, and also promote synaptogenesis in adjacent astrocytes [40]. Through this non-destructive strategy, B2BM cells form pseudo-tripartite synapses to capture extracellular glutamate and other neurotransmitters, enhancing survival and expansion within the brain microenvironment [40]. Future studies are needed to elucidate how tumor cells mimic neuronal connectivity to integrate host neural cues, and whether disrupting these synaptic-like interactions could impair tumor adaptation and growth in the CNS.

The mechanisms underlying neural-tumor interactions are not universally conserved across cancer types [98]. While processes such as neurotrophin-mediated axonogenesis, neuro-tumor electrical coupling, and neural modulation of immune responses have been reported in various solid tumors [26–28, 94, 95, 99], considerable heterogeneity exists in neural dependency, degree of innervation, neural subtype composition, and responsiveness to neural signals [14]. For instance, gliomas form functional synapses with neurons and exhibit pronounced reliance on neural electrical activity, whereas such phenomena are absent in colorectal cancer (CRC) [94, 95]. Similarly, parasympathetic innervation appears to suppress tumor progression in PC but promotes invasion in PCa [100]. These divergent observations underscore the importance of analyzing tumor-neuron crosstalk in a tumor-type-specific context, with consideration of biological factors such as cancer stage, tissue origin, and microenvironmental conditions. Tailoring mechanistic investigations and therapeutic strategies accordingly may enhance the precision and efficacy of interventions targeting neural pathways in oncology.

## Updated strategies for tumor innervation disruption

In recent years, the emergence of cancer neuroscience has transformed our understanding of tumor biology, positioning the nervous system as a key regulator of cancer progression. In this section, we systematically synthesize 9 emerging strategies for disrupting neural-tumor symbiosis: inhibiting tumor growth, overcoming drug resistance, enhancing anti-tumor immunity, preventing invasion and metastasis, modulating neural electrical activity, alleviating neuropathic pain, balancing the stress-hypothalamic-pituitary-adrenal (HPA) axis, elucidating brain-body communication in tumor progression, and exploring bioelectric neuroimmunotherapy (Table 1) [26, 28, 59, 84, 94, 95, 97, 101–168]. By integrating preclinical discoveries with early clinical evidence, we assess the translational potential of these approaches and propose a strategic roadmap for therapeutically targeting tumor innervation.

**Table 1:** Evidence and targets for targeting the innervation tumor microenvironment

Tumor type	Therapeutic concept	Mechanism and target	Contribution to cancer	Therapeutic approaches/representative drugs	Reference
Melanoma	Tumor growth	SCs (nerve restoration)	Selective transection of sensory nerves in the dorsal skin led to rapid melanoma proliferation during Wallerian degeneration and SC transdifferentiation to rSCs	Minocycline	[135, 147, 148]
Cervical cancer	Tumor growth	Neurogenesis	NGF and TrkA were distinctly overexpressed in SCC, compared to AC and normal cervical tissue, and were associated with a higher grade for SCC	GNF-5837	[130, 160]
Gastric cancer	Tumor growth	Cholinergic signaling (NGF, Wnt pathway)	Vagal innervation induces NGF secretion via acetylcholine signaling, promoting gastric tumorigenesis through Wnt activation	Silencing M3R with siRNAs	[113]
Thyroid cancer	Tumor growth	Neural-tumor crosstalk (MAPK/ERK and PI3K/Akt pathways)	NrCAM depletion markedly curbs thyroid cancer cell growth and tumorigenicity	NrCAM antibody	[142]
Glioma	Tumor growth	Protein signalling (NLGN3, ADAM10)	NLGN3, a synaptic adhesion protein, is released from neighboring non-glioma neurons and cleaved by ADAM10 in an activity-dependent manner. The resultant NLGN3 then promotes growth via a PI3K-mTOR pathway	INCB7839	[168]
Pancreatic cancer	Stress adaptation	Axonal recruitment (NGF)	In nutrient-poor, serine-deprived desmoplastic environments, pancreatic cancer cells increase NGF production to recruit axons for L-serine acquisition	Larotrectinib	[143]
	Stress adaptation	Axonal recruitment (NGF)	β1-containing integrins sense ECM stiffness, and YAP1 relays this signal to the nucleus to induce the expression of neurotrophic genes like BDNF and NGF	–	[28]
Colon cancer	Stress adaptation	Axonal recruitment (proBDNF)	ER stress in cancer cells induces XBP1 expression, which stimulates proBDNF secretion and promotes neurite outgrowth by upregulating EGLN3 via c-Myc in neuronal cells	Anti-proBDNF antibody	[26]
Lung cancer	Stress adaptation	Metabolic reprogramming	Under metabolic stress, NSCLC cells expressing HTR1D increase glucose uptake to gain a growth advantage via the 5-HT-mediated Warburg effect and control metabolic reprogramming by activating the PI3K/Akt/mTOR signaling pathway	LY294002	[167]
Glioma	Stress adaptation, electrical hyperactivity, and seizures	Microtube formation (connexin 43 gap junctions)	Glioma cells form microtubes that enable direct communication with surrounding cells via connexin 43-mediated gap junctions, propagating calcium waves linked to treatment resistance	Vinorelbine	[150, 161]
Optic gliomas	Immune remodelling	Neuron-immune-cancer axis	Meningeal T cells activated by MDK from Nf1-mutant retinal ganglion cells boost CCL4 production by CD8^+^ T cells, triggering NF-κB-dependent CCL5 upregulation in microglia. This promotes optic glioma growth by inhibiting cancer stem cell apoptosis and enhancing tumor progression via the Akt/GSK3β/CREB pathway	–	[129]
Melanoma	Immune remodelling	Sensory nerve innervation	Skin sensory nerves impede the maturation of intratumoral HEVs and limit the formation of mature tertiary lymphoid structures containing organized CD4^+^ T cells and B cell clusters	Surgical or chemical skin sensory denervation	[117]
	Immune remodelling	SCs	SCs upregulate MAG to recruit MDSCs via chemotaxis in the tumor microenvironment	Radiation-induced elimination of SCs	[139]
Breast cancer	Immune remodelling	Sema4D	The absence of Sema4D significantly impedes tumor growth and metastasis in mice	Sema4D-directed antibody	[132]
Ovarian cancer	Metastasis	β-adrenergic signaling	NE promotes sphere formation of FTE cells in ULA culture and enhances resistance to apoptosis in a β-AR-dependent manner	β-AR blocker	[105]
Brain metastases	Metastasis	Anoikis resistance (TrkB)	TrkB, a neurotrophic receptor, suppresses anoikis and promotes metastasis by enabling cancer cell circulation	TrkB antagonist	[163]
Osteosarcoma	Metastasis	Protein signaling (NGF)	NGF promotes MMP-2-dependent cell migration by inhibiting the effects of miR-92a-1-5p via the MEK/ERK signaling cascade	Lipocalin-2, Larotrectinib	[106]
Salivary adenoid cystic carcinoma	Metastasis	Protein signaling (CXCL12)	CXCL12/CXCR4 promotes tumor cell EMT and triggers PNI through the activation of the Twist/S100A4 pathway	AMD3100	[122]
Pancreatic cancer	Metastasis	Glutamate signaling (AMPAR)	In PDAC cells, neuronal glutamate induces calcium influx via NMDAR, activating the CaMKII/ERK-MAPK pathway. This upregulates METTL3 transcription, whose mRNA is modified by m^6^A, leading to increased HK2 expression and enhanced PNI	STM2457	[101, 114]
	Metastasis	Protein signaling (CCL2)	In pancreatic cancer, TGF-α induces DRG neurons to secrete CCL2, which promotes paxillin phosphorylation and cytoskeletal remodeling via CCR4, facilitating tumor cell migration toward nerves	C021, 6-B345TTQ	[110]
	Metastasis	Exosomes (lncRNA XIST)	Exosomes transfer lncRNA XIST to neural cells, acting as a sponge for miR-211-5p to relieve its inhibition of GDNF, thereby enhancing pancreatic cancer cell PNI	GW4869	[108]
Brain metastases	Metastasis, electrical hyperactivity, and seizures	Direct synapse formation (NMDAR)	Breast cancer cells form astrocyte-like synapses (pseudo-tripartite synapses) to access neuronal glutamate, activating the NMDAR pathway and promoting brain colonization	NMDAR antagonist	[97]
	Metastasis	Neurochemical signaling (GABA)	Breast cancer brain metastases upregulate GABA receptors and GABA production	Bilobalide, Picrotoxin	[131, 154]
	Metastasis	Neurochemical signaling (GABA)	Metastatic brain tumors upregulate the expression of ABAT to degrade neuronal GABA, thereby generating energy	Picrotoxin	[115]
Glioma	Lectrical hyperactivity and seizures, tumor growth	Direct synapse formation (AMPAR)	Nerves form direct glutamatergic synapses with glioma cells, modulating tumor microtube-mediated invasion	AMPAR antagonist	[94, 95]
	Electrical hyperactivity and seizures	Glutamate signaling (AMPAR)	Gliomas release high levels of glutamate, which promotes tumor growth via Ca^2^⁺-permeable AMPA receptors, establishing a nerve-tumor hyperactivity and proliferation feedback loop	AMPAR antagonist	[94]
	Electrical hyperactivity and seizures	Protein signaling (BDNF)	BDNF enhances the surface transport of AMPA receptors on glioma cells, amplifying glutamate-induced currents and calcium transients	NTRK2 antagonist	[112]
	Electrical hyperactivity, seizures, and tumor growth	Neurochemical signaling (GABA)	Gliomas downregulate inhibitory GABA currents in the surrounding tumor microenvironment	GABA inhibitor	[60, 152]
	Electrical hyperactivity, tumor growth,	Thrombospondin-1	The thrombospondin-1 secreted by glioma will promote the enhancement of functional neuronal connections between the tumor and the brain	Gabapentin	[119]
	Electrical hyperactivity	SCs (collagen 1a2)	In NF1-deficient neurofibromas, sensory axons extending to NF1-mutant dorsal root ganglion neurons exhibit higher action potential issuance than wild-type controls, mediated by increased collagen 1a2 expression	TTX	[121]
Pancreatic cancer	Neuropathic pain	Inflammation around nerves	Mast cells are specifically enriched in intrapancreatic nerves, correlating with neuropathic abdominal pain in pancreatic cancer patients	Mast cell degranulation inhibition	[157]
Lung carcinoma	Neuropathic pain	Macrophage to neuron-like cell transformation (MNT)	MNTs exhibit nociceptive activity under cancer conditions. Genetic and pharmacological targeting of Smad3 effectively blocks MNT-driven tumor innervation	SIS3	[118, 141]
Melanoma	Neuropathic pain	Peripheral nerve resident macrophages and SCs (TRPA1)	Activation of SCs TRPA1 releases M-CSF, sustaining rMΦ expansion and inducing oxidative stress, which targets neuronal TRPA1 to generate pain signals	A967079	[126]
Bone metastasis	Neuropathic pain	Sensory nerve (TRPV1^+^)	In mouse bones, CGRP^+^ sensory nerves are prevalent, and TRPV1 activation by the acidic cancer microenvironment in bone promotes SN activation and bone pain	SB366791, Bafilomycin A1	[123, 134, 140, 145]
Pancreatic cancer	Neuropathic pain	Neurotrophin signalling	NGF and other neurotrophins activate TRPV1. TRPV1 activation triggers membrane depolarization and release of substance P or CGRP to transmit pain signals	CGRP_8-37_	[102, 162]
Breast cancer	Stress-HPA axis	Systemic signals	Glucocorticoids drive stress-induced metastasis by promoting both metastasis and immune dysfunction	GR antagonist	[107, 116, 124, 128, 133, 138, 144, 146, 149, 151, 158]
Cancer cachexia	Brain-body communication	Area postrema neurons	Blocking pro-inflammatory signaling (e.g., IL-6) and inhibiting AP network over-activation can effectively regulate systemic metabolic and immune responses, restore metabolic homeostasis, and slow cachexia progression	Anti-IL-6 antibody, suppression of Il6ra in AP neurons	[103, 104]
Breast cancer	Brain-body communication	Neurons and neural circuits	Activating CeM^CRH^ neurons and their projections to the LPGi increases anxiety-like behavior and accelerates tumor growth in mice	Alprazolam	[84, 109, 153, 155, 156]
	Brain-body communication	Neurons and neural circuits	Chemical genetics reveals that stimulating PVN^CRH^ neurons during specific circadian phases restores GC rhythms, slows tumor growth, and increases intratumoral CD8^+^ T cells	Chronotherapy	[136, 137, 165, 166]
Glioma	Brain-body communication	Neurons and neural circuits	Olfactory experience directly modulates glioma formation, with ORN activity affecting glioma development	PPP, AXL1717	[120, 127]
Normal tissue	Bioelectronic neuro-immunology	Neurons and neural circuits	High-intensity electroacupuncture at the foot-sanli point activates the sympathetic-splenic axis and induces norepinephrine, preventing systemic inflammation. In contrast, low-intensity electroacupuncture at the same point activates the vagus nerve-adrenal axis, inhibiting inflammation	Electroacupuncture	[125, 164]
Breast cancer	Bioelectronic neuro-immunology	Neurons and neural circuits	Vagus nerve stimulation enhances TFF2 expression, while bilateral subdiaphragmatic vagotomy in mice abolishes splenic TFF2 responses, leading to increased MDSCs and colonic carcinogenesis	Semapimod	[111, 159]

*5-HT* 5-hydroxytryptamine, *ABA* amylobarbitonic acid, *AC* adenocarcinoma, *ADAM10* a disintegrin and metalloproteinase 10, *AMPA* α-amino-3-hydroxy-5-methyl-4-isoxazolepropionic acid, *AMPAR* AMPA receptor, *AP* area postrema, *AXL1717* XL1171 AXL inhibitor, *BDNF* brain-derived neurotrophic factor, *CaMKII/ERK-MAPK* calcium/calmodulin-dependent protein kinase II/extracellular signal-regulated kinase mitogen-activated protein kinase, *CCL2* chemokine (C-C motif) ligand 2, *CCL5* chemokine (C-C motif) ligand 5, *CCR4* C-C chemokine receptor type 4, *CeMC* central medial amygdala cortex, *CGRP-*_8-37_ CGRP_8-37_- fragment, *CGRP* calcitonin gene-related peptide, *c-Myc* cellular myelocytomatosis oncogene, *CRH* corticotropin-releasing hormone, *CXCL12/CXCR4* chemokine (C-X-C motif) ligand 12/chemokine (C-X-C motif) receptor 4, *DRG* dorsal root ganglion, *ECM* extracellular matrix, *EGIL3* egl nine homolog 3, *EMT* epithelial-mesenchymal transition, *ER* endoplasmic reticulum, *ERK* extracellular signal-regulated kinase, *FTE* fallopian tube epithelial, *GABA* γ-aminobutyric acid, *GC* glucocorticoids, *GDN* glial cell line derived neurotrophic factor, *GR* glucocorticoid receptor, *HEVs* high endothelial venules, *HK2* hexokinase 2, *HTR1D* 5-hydroxytryptamine receptor 1D, *IL-1β* interleukin 1 beta, *IL-6* interleukin 6, *IL-6R* interleukin 6 receptor, *IncRNA* long non-coding RNA, *LPGi* lateral paragigantocellular nucleus, *M3R* muscarinic receptor 3, *m*^*6*^*A* N^6^-methyladenosine, *MAG* myelin-associated glycoprotein, *Mast* mast cell, *M-CSF* macrophage colony-stimulating factor, *MDK* macrophage-derived growth factor, *MDSCs* myeloid-derived suppressor cells, *MEK* mitogen-activated protein kinase kinase, *METTL3* methyltransferase like 3, *MMP-2* matrix metallopeptidase 2, *MNT* macrophage neuron-like transformation, *MTT* macrophage-like transformation, *NE* nerve ending, *NF1* neurofibromin, *NF-κB* nuclear factor kappa-B, *NGF* nerve growth factor, *NLGN3* neuroligin 3, *NMDAR* N-methyl-D-aspartate receptor, *NrCAM* neuronal cell adhesion molecule, *NSCLC* non-small cell lung cancer, *ORN* olfactory receptor neurons, *PDAC* pancreatic ductal adenocarcinoma, *PI3K/Akt/mTOR* phosphoinositol 3-kinase/Akt/mechanistic target of rapamycin, *PI3K-mTOR* phosphoinositol 3-kinase/mechanistic target of rapamycin, *PNI* perineural invasion, *PPP* para-pineal pigment, *PVN* paraventricular nucleus, *rMΦ* reactive macrophages, *SCs* Schwann cells, *SCC* squamous cell carcinoma, *Sem* semaphorin, *Smads* small mother against decapentaplegic, *SN* sensory neurons, *TF2* transferrin 2, *TFF2* trefoil factor 2, *TrkA* tropomyosin receptor kinase A, *TrkB* tropomyosin receptor kinase B, *TRPA1* transient receptor potential ankyrin 1, *TRPV1* transient receptor potential vanilloid 1, *TTX* tetrodotoxin, *Twist/S100A4* twist family BHLH transcription factor 1/S100 calcium binding protein A4, *ULA* ultra-low adhesion, *Wnt* wingless/integrated 1, *XBP1* X-box binding protein 1, *XIST* X(inactive)specific transcript, *YAP1* Yes1 associated transcriptional regulator 1, *β-AR* β-adrenergic receptor

### Inhibiting tumor growth via modulation of the nervous system

Emerging evidence highlights the nervous system as a pivotal driver of tumor growth, survival, and progression, offering novel avenues for therapeutic intervention [169]. A range of strategies is under investigation to modulate neural influence on cancer, including blockade of growth signals, receptor-specific targeting, and both surgical and pharmacological denervation [170]. Each of these approaches holds transformative potential, representing a paradigm shift in the way cancer may be treated through neural pathway modulation.

An initial strategy for disrupting neural-tumor interactions involves reducing nerve density within the TME, either by eliminating existing nerves or by preventing neurogenesis. This can be accomplished through surgical or pharmacological denervation, such as chemical denervation or botulinum toxin administration, which have been shown in PC models to significantly reduce tumor volume [171, 172]. Neural ablation, a strategy aimed at selectively removing or inhibiting specific nerve populations within the TME, is not universally beneficial and appears to depend on both nerve subtype and tumor context. Sensory nerve ablation, for instance, can exacerbate melanoma progression by triggering SC-mediated repair-like programs [173]. In contrast, parasympathetic denervation in PC increases tumor incidence [72, 73]. Conversely, in the same PC context, sensory nerves contribute to tumorigenesis via TRPV1/TRPA1-mediated neurogenic inflammation, and their ablation suppresses disease initiation [74, 75]. These contrasting findings underscore the complexity of nerve-tumor crosstalk and highlight the need for nerve-type-specific and context-dependent therapeutic strategies.

**Fig. 3:**
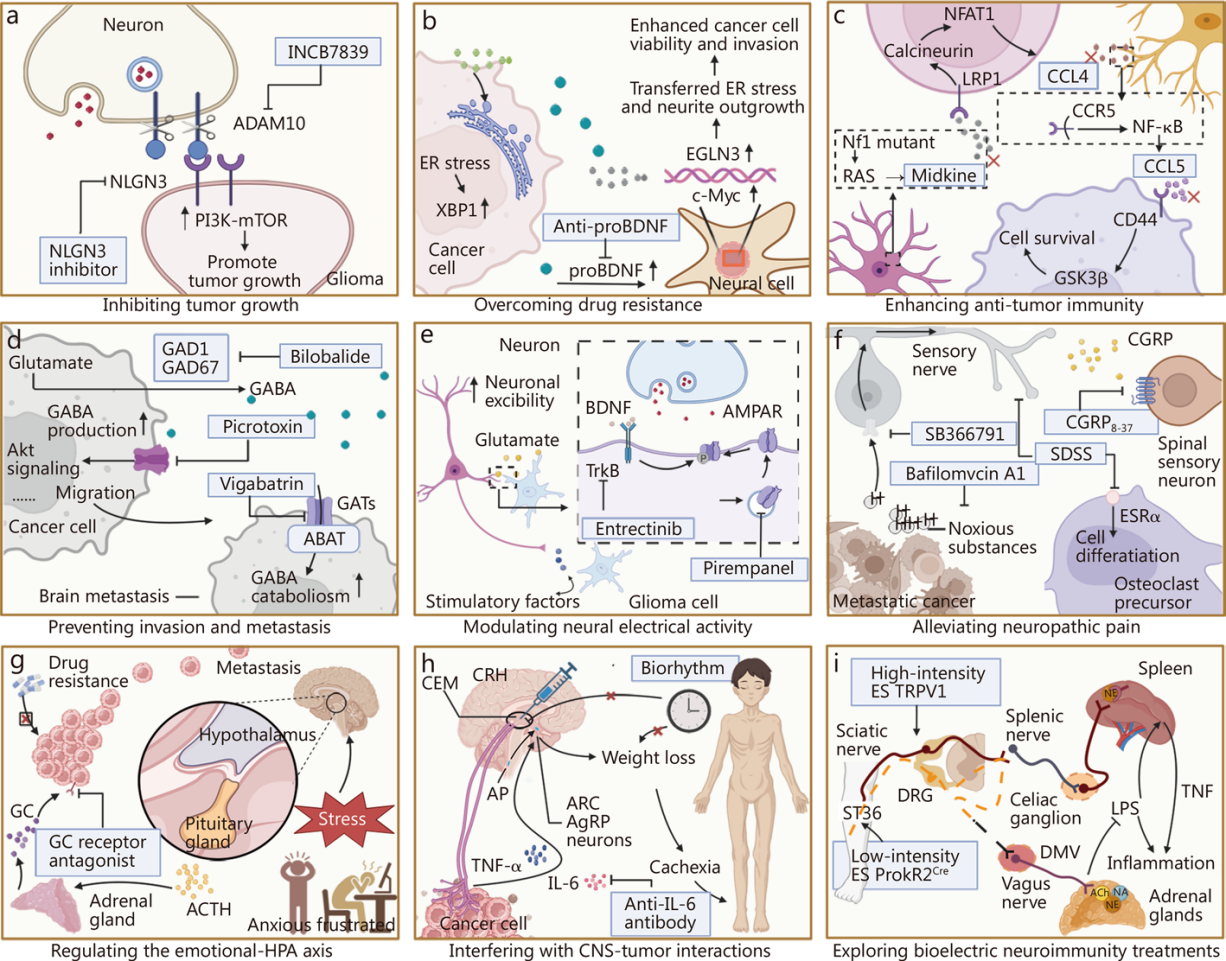
The updated strategies in tumor innervation disruption. This figure mainly introduces some examples of updated strategies for targeting tumor innervation. **a** Inhibiting tumor growth by blocking ADAM10 and NLGN3. **b** Overcoming drug resistance by targeting increased innervation caused by ER stress. **c** Reshaping the tumor immune microenvironment by regulating the neuro-immune-tumor axis. **d** Reducing tumor metastasis by preventing distant tumor colonization. **e** Inhibiting tumor growth by disrupting nerve electrical activity in gliomas. **f** Alleviating metastatic bone pain by targeting sensory nerves. **g** Reducing tumor resistance by blocking glucocorticoid receptors. **h** Controlling tumor progression by targeting central nervous system (CNS) neurons and regulating biological rhythms. **i** Inhibiting systemic inflammation by stimulating the release of regulatory neurotransmitters through electroacupuncture. This figure was created using BioRender (https://biorender.com/). ADAM10 a disintegrin and metalloproteinase domain-containing protein 10, NLGN3 neuroligin-3, ER endoplasmic reticulum, INCB7839 aderbasib, PI3K phosphoinositide 3 kinase, mTOR mechanistic target of rapamycin, XBP1 X-box binding protein 1, BDNF brain-derived neurotrophic factor, EGLN3 Egl-9 family hypoxia inducible factor 3, c-Myc cellular myelocytomatosis oncogene, NFAT1 nuclear factor of activated T cells 1, CCL4 C-C motif chemokine ligand 4, CCL5 C-C motif chemokine ligand 5, LRP1 low-density lipoprotein receptor-related protein 1, NF1 neurofibromin 1, RAS rat sarcoma, CCR5 C-C chemokine receptor type 5, NF-κB nuclear factor kappa-light-chain-enhancer of activated B cells, CD44 cluster of differentiation 44, Akt protein kinase B, GAD1 glutamate decarboxylase 1, GAD67 glutamate decarboxylase 67, GABA γ-aminobutyric acid, GATs GABA transporters, ABAT 4-aminobutyrate aminotransferase, AMPAR α-amino-3-hydroxy-5-methyl-4-isoxazolepropionic acid receptor, TrkB tropomyosin receptor kinase B, CAMKII calmodulin-dependent protein kinase II, CGRP calcitonin gene-related peptide, ESRα estrogen receptor alpha, GC glucocorticoid, ACTH adrenocorticotropic hormone, CEM central medial nucleus of the amygdala, CRH corticotropin-releasing hormone, AP area postrema, TNF-α tumor necrosis factor-α, IL-6 interleukin-6, ARC arcuate nucleus of the hypothalamus, AgRP agouti-related peptide, ES electrical stimulation, TRPV1 transient receptor potential vanilloid 1, DRG dorsal root ganglion, ST36 zusanli, PROKR2^Cre^ prokineticin receptor 2 Cre recombinase, DMV dorsal motor nucleus of the vagus, LPS lipopolysaccharide, GSK3β glycogen synthase kinase 3β

Melanoma has been shown to activate repair-like programs in SCs, while minocycline suppresses tumor growth by inhibiting neurotomy-induced SC priming [135, 147, 148]. However, these findings remain exploratory, and further large-scale, mechanistically refined studies are required to confirm functional involvement and elucidate the underlying regulatory pathways. Neurotrophic factors play a central role in promoting neurogenesis. In preclinical models of cervical and breast cancer, anti-NGF antibodies and the Trk tyrosine kinase inhibitor GNF-5837 have demonstrated promising anti-tumor efficacy [130, 160], though further clinical validation is needed to establish therapeutic relevance. Targeted blockade of neural signaling has emerged as a promising strategy for therapeutic intervention. Inhibition of the NGF/Trk axis has been shown to suppress epithelial proliferation and tumorigenesis in a muscarinic M3R-dependent manner [174]. Silencing M3R using small interfering RNAs (siRNAs) also reduced epithelial cell proliferation and significantly decreased tumor size and number, without impairing normal gastrointestinal development [113]. In thyroid cancer, depletion of neuronal cell adhesion molecule (NrCAM) markedly impaired tumor growth and tumorigenic potential, and high-affinity NrCAM antibodies may offer a novel therapeutic option for NrCAM-positive malignancies [142]. Additionally, inhibition of ADAM10 prevents the release of NLGN3 into the TME, thereby suppressing high-grade glioma xenograft growth by blocking NLGN3-mediated activation of the phosphoinositol 3-kinase (PI3K)/mechanistic target of rapamycin (mTOR) signaling pathway. INCB7839, a selective ADAM10 inhibitor, has been developed for clinical application [168] (Fig. 3a). The repurposing of neuromodulatory agents such as dopamine receptor modulators also shows promise. ONC201 [175] and the second-generation imipridone ONC206, which targets dopamine receptor D2, are currently in phase I and II trials for several tumor types, including endometrial and neuroendocrine cancers [176]. However, larger randomized controlled trials are necessary to determine whether these agents can translate into improved clinical outcomes.

This strategy is particularly relevant for tumor types characterized by high neural embedding density, active neural signaling, or marked dependence on neurotrophic factors, such as PC and PCa, where neuro-mediated growth-promoting mechanisms are well established. In oncology, the concept of drug repurposing presents an appealing therapeutic avenue, offering renewed potential for intervention. Combining pharmacological approaches with surgical denervation has been shown to enhance treatment precision, improve tumor-specific targeting, minimize collateral damage to surrounding healthy tissue, and optimize therapeutic efficacy.

### Counteracting treatment resistance with cellular stress-adaptive mechanism interventions

A hallmark of aggressive cancers is their exceptional adaptive capacity, enabling sustained proliferation under adverse conditions such as nutrient deprivation or systemic therapy. Recent studies implicate neural-derived factors, such as neuropeptide Y (NPY), in facilitating tumor survival in such stressful microenvironments [177–179]. For example, NPY has been proposed to confer protection against radiation-induced apoptosis, potentially contributing to therapeutic resistance and poor clinical outcomes [177]. Highly stress-adaptive tumors, such as those in breast cancer and PC, frequently develop treatment tolerance by activating anti-apoptotic signaling or reprogramming metabolic pathways through neural inputs, particularly under hypoxia, nutrient restriction, metabolic stress, or therapy-induced stress [26–28, 167]. As a result, these neural pathways represent critical adaptive targets for disrupting stress tolerance mechanisms and improving the treatment of drug-resistant cancers.

ER stress, nutrient deprivation, and mechanical stress within tumors can promote the secretion of neurotrophic factors, thereby stimulating nerve growth and enhancing tumor resistance to therapeutic damage [26–28]. ER stress is extensively investigated. ER stress in cancer cells induces X-box binding protein 1, which promotes proBDNF release. The released proBDNF subsequently activates cellular myelocytomatosis oncogene (c-Myc)-regulated Egl-9 family hypoxia inducible factor 3 in neurons, enhancing cancer cell survival and resistance to therapy (Fig. 3b). Therefore, targeting neurotrophic signals such as NGF, BDNF, and their respective receptors offers a promising strategy to disrupt tumor adaptive responses [26–28]. Notably, combination therapy using a low-serine diet and larotrectinib, a highly selective Trk inhibitor, has been shown to counteract tumor adaptation by impairing serine-dependent nerve recruitment [143]. Furthermore, several chemotherapeutic agents, including 5-fluorouracil, are known to induce ER stress in cancer cells [180], a response that can be attenuated by anti-proBDNF antibodies [26].

Nerves also regulate cancer cell behavior under metabolic stress, promoting tumor survival in adverse microenvironmental conditions. NGF promotes neural infiltration in non-small cell lung cancer (NSCLC), while 5-hydroxytryptamine (5-HT), which is secreted by nerves, is markedly increased in tumors exhibiting extensive neural invasion. In NSCLC, cells expressing 5-HT receptor 1D enhance glucose uptake and proliferation via 5-HT-mediated activation of the Warburg effect, thereby driving metabolic reprogramming [167]. Highly glycolytic tumor cells can suppress T cell infiltration and cytotoxic activity within the TME, contributing to resistance against T cell-based immunotherapies [181, 182]. Pharmacological inhibition of this metabolic phenotype using LY294002, a PI3K inhibitor, or rapamycin, a mTOR complex 1 inhibitor, has been shown to impair 5-HT-induced glycolytic responses in NSCLC models [167].

In CNS cancers, ultra-long membranous protrusions, referred to as TMs, have been identified as a key mechanism contributing to treatment resistance in gliomas [183]. The TMs connecting glioma cells or tumor and non-tumor astrocytes form functional networks that enhance intercellular communication and stress resistance, thereby promoting tumor malignancy. Through calcium waves and gap junctions, these TMs increase the tumor’s resistance to radiation and chemotherapy. Neurons form AMPAR synapses on TMs with glioma cells, and neuronal AMPAR input significantly increases the length, number, and branching of TMs [183, 184]. Non-connecting TMs primarily enhance the invasiveness of gliomas, likely related to their role in enhancing cell motility [183]. Knockdown of βIII tubulin (*TUBB3*) in a brain-metastatic breast cancer cell line significantly reduced metastatic capacity in vivo and improved survival in a brain metastasis model [150]. In patients with systemic lung cancer exhibiting TUBB3 overexpression, the anti-microtubule agent vinorelbine has been shown to significantly prolong progression-free survival [161]. Ongoing studies are exploring small-molecule inhibitors targeting growth-associated protein 43, a critical regulator of membrane tube extension. These inhibitors hold promise in disrupting tumor microtubule formation and function, offering a novel therapeutic strategy for glioma treatment [185].

Overcoming tumor drug resistance requires disrupting the adaptive capacity that enables aggressive cancers to thrive under hostile conditions. By targeting neural-tumor interactions or interfering with the metabolic stress responses of tumor cells, their survival mechanisms within adverse microenvironments can be effectively impaired. Accordingly, future therapeutic strategies should shift toward regulating tumor stress responses through the combined modulation of multiple pathways, rather than relying solely on conventional cytotoxic therapies.

### Enhancing anti-tumor immunity by remodeling the immune microenvironment

The tumor immune microenvironment (TIME) is a dynamic ecosystem in which neurons and immune cells engage in bidirectional crosstalk [186]. Targeting these neuro-immune axes offers a promising strategy to overcome immune evasion and reprogram the TIME, opening new avenues for multimodal cancer interventions. This approach is particularly relevant for tumors characterized by pronounced immunosuppression and dense neuro-immune interactions within the TME, such as GBM and PC, which typically exhibit poor responses to immune checkpoint blockade [187, 188].

In the CNS, neuron-immune interactions play a critical role in tumor progression. In optic pathway gliomas, meningeal T cells infiltrate the tumor in an integrin-dependent manner and are activated by midkine secreted from NF1-mutant retinal ganglion cells (neurons), leading to increased production of C-C motif chemokine ligand (CCL) 4 by CD8⁺ T cells [129]. This, in turn, triggers NF-κB-dependent upregulation of CCL5 in microglia, a signaling cascade that promotes glioma growth by inhibiting CSCs apoptosis and enhancing tumor progression [129] (Fig. 3c). Additionally, neuronal expression of PD-L1 in brain tissue adjacent to tumors is positively correlated with GBM patient survival. Neuronal PD-L1 induces caspase-dependent apoptosis in glioma cells, and its expression is further enhanced by interferon-β stimulation [187].

Peripheral neurons are integral components of the TME, and sensory innervation has been shown to suppress effective anti-tumor immune responses [117]. In cutaneous tumors, sensory nerves impair the maturation of high endothelial venules and limit the formation of mature tertiary lymphoid structures, including organized clusters of CD4^+^ T cells and B cells [117]. Ablation of sensory nerves enhances leukocyte infiltration, increases T cell clonality, and expands the intratumoral B cell pool [118]. Additionally, SCs modulate immune activity by upregulating myelin-associated glycoprotein [139], which promotes the chemotactic recruitment of MDSCs. These MDSCs, in turn, induce apoptosis of tumor-infiltrating lymphocytes. Notably, combining FasL-neutralizing antibodies with immune checkpoint blockade enhances therapeutic efficacy. Specifically, dual PD-1 and cytotoxic T-lymphocyte-associated antigen 4 (CTLA-4) inhibition, when administered with a FasL-neutralizing antibody, results in a significantly greater reduction in tumor burden than checkpoint blockade alone [139, 189].

The TME comprises diverse immune cell populations, including T cells, regulatory T cells, macrophages, MDSCs, and dendritic cells [190]. These cells collectively shape an inflammatory milieu that can either promote or suppress tumor progression [9]. A range of signaling pathways and molecular mediators contribute to immune reprogramming, particularly through macrophage modulation. Axon guidance molecules represent key targets in this context. In PDAC, tumor- and nerve-derived Sema3D reprogram macrophages indirectly via *KRAS* mutation-dependent adenosine diphosphate-ribosylation factor 6 (ARF6) signaling in tumor cells. This signaling cascade increases lactate production, which is sensed by G protein-coupled receptors on macrophages, promoting their differentiation into an immunosuppressive phenotype that fosters tumorigenesis [188]. In mouse models, deletion of *Sema4D* significantly inhibits tumor growth and metastasis [191]. Combined treatment with anti-Sema4D antibodies and immune checkpoint inhibitors enhances the recruitment of effector lymphocytes and antigen-presenting cells while reducing immunosuppressive populations, leading to effective TME remodeling and tumor rejection [192]. However, in a neuroendocrine PC model, anti-Sema4D therapy, while inhibiting tumor growth and prolonging survival, unexpectedly promoted tumor invasiveness through retrograde signaling mediated by macrophages [132]. These findings highlight the dual and context-dependent roles of certain immunomodulatory factors in the TME, underscoring the need for carefully tailored therapeutic strategies.

The intricate interplay among signaling molecules, immune cells, and neurons within the TME shapes the mechanisms underlying tumor immune evasion. Advancing personalized cancer therapies, such as combination immunotherapy, neuromodulatory interventions, and targeted approaches against specific cytokines or signaling pathways, holds promise for reprogramming the immune landscape of tumors. Such strategies are expected to enhance therapeutic efficacy and improve patient outcomes by more effectively modulating the TME and overcoming resistance to conventional treatments.

### Targeting metastatic spread

Tumor invasion and metastasis are complex, multi-step processes involving intrinsic changes in tumor cell behavior and dynamic interactions with the surrounding microenvironment. In cancer types with pronounced metastatic potential, such as breast cancer and PCa, neuro-induced epithelial-mesenchymal transition and PNI are frequently observed [8, 193]. In these contexts, metastatic dissemination is closely linked to the activity of local neural remodeling. Tumor-associated innervation plays a critical role in this process, offering novel and promising therapeutic targets for intervention.

During the early stages of metastatic dissemination, tumor cells must acquire resistance to anoikis, apoptosis induced by detachment from the extracellular matrix. NE has been shown to promote sphere formation in fallopian tube epithelial cells under ultra-low adhesion conditions and confer resistance to apoptosis in a β-AR-dependent manner. This effect is primarily mediated by the β2-AR subtype and can be effectively blocked by the β-AR antagonist propranolol [105]. Colony-stimulating factor 2 may act downstream of NE to mediate this anti-anoikis effect [105]. Neurotrophic receptors, particularly TrkB, also play a pivotal role in anoikis resistance, thereby facilitating metastatic spread [163]. These findings provide a rationale for the use of TrkB inhibitors, such as larotrectinib and entrectinib, in locally advanced malignancies to induce tumor cell death before colonization of distant organs.

Enhanced tumor cell motility is a prerequisite for metastatic dissemination. NPY/NPY receptor type 1 (NPY1R) signaling has emerged as a previously unrecognized anti-metastatic target in PC. Both NPY and its receptor, NPY1R, are upregulated in mouse models and human PC tissues. Pancreas-specific and systemic knockout of *NPY1R* significantly reduced liver metastasis in PC mouse models, while treatment with the NPY1R antagonist BIBO3304 markedly impaired cancer cell migration on cell-derived matrices [194]. Similarly, pharmacological antagonism of NPY1R and NPY5R effectively blocked NPY-induced motility and invasion in breast cancer cells [195]. In parallel, NGF promotes activation of the mitogen-activated protein kinase kinase (MEK)/ERK signaling cascade through phosphorylation of MEK [106]. Bioinformatic analyses identified MMP-2 as a direct downstream target of miR-92a-1-5p, which is suppressed by NGF stimulation. In osteosarcoma cells, inhibition of MEK or ERK, via small molecule inhibitors or siRNA, reversed NGF-mediated downregulation of miR-92a-1-5p [106] (Fig. 2e). These findings nominate MEK and ERK inhibitors, lipocalin-2, and the Trk inhibitor larotrectinib as potential agents for targeting NGF-driven metastasis in osteosarcoma.

During local invasion, tumor cells often exploit nerves as physical conduits, a phenomenon known as PNI, to facilitate metastatic spread [196]. In PDAC, neuronal glutamate release activates NMDAR on tumor cells, triggering calcium influx and promoting the transcription of methyltransferase-like 3 (METTL3). *METTL3* mRNA undergoes m^6^A methylation, leading to upregulation of hexokinase 2, thereby enhancing glycolysis and facilitating PNI [114]. Inhibitors targeting METTL3, such as STM2457, suppress tumor cell metabolism by blocking m^6^A modification, significantly reducing invasive and metastatic potential [101]. Similar neuro-metastatic mechanisms have been identified in other cancers. In salivary adenoid cystic carcinoma, C-X-C motif chemokine ligand 12 and C-X-C chemokine receptor type 4 promote epithelial-mesenchymal transition and PNI [122]. In PCa, transforming growth factor-α induces dorsal root ganglion (DRG) neurons to secrete CCL2, which drives paxillin phosphorylation and cytoskeletal remodeling, facilitating tumor migration toward nerves [110]. Regarding therapy, a recent study suggests that inhibition of exosomal lncRNA XIST delivery using GW4869 blocks glial cell line-derived neurotrophic factor (GDNF)-mediated PNI, providing a potential strategy to interfere with neurotropic metastasis [108]. Angiogenesis also plays a supporting role in neuroinvasion, with nerve signaling closely linked to vascular remodeling. In TNBC, inhibition of metabotropic glutamate receptor 1 using BAY36-7620 or riluzole significantly reduces tumor angiogenesis and growth [197]. Additionally, anti-VEGF agents such as sunitinib effectively normalize aberrant tumor vasculature and reduce pathological angiogenesis [198].

The metastatic cascade culminates in the successful colonization of distant organs. In brain metastases, γ-aminobutyric acid (GABA)-induced proliferation of tumor cells is driven by upregulation of glutamate decarboxylase (GAD)67, which converts glutamate to GABA. This metabolic adaptation enables tumor cells to utilize GABA as a nutrient source, producing succinate and nicotinamide adenine dinucleotide for biosynthetic and energetic support [131, 154]. Additionally, metastatic tumor cells in the brain increase expression of 4-aminobutyrate aminotransferase to catabolize neuronal GABA, further contributing to energy generation [115]. The GABA transaminase inhibitor aminocaproic acid has been proposed as a potential therapeutic agent in this context [154]. Beyond metabolic adaptation, cancer cells can also secrete soluble factors and EVs, collectively termed tumor-derived mediators, that act on distant tissues to induce the formation of pre-metastatic niches [199]. This paradigm expands our understanding of metastatic progression and provides additional opportunities for therapeutic intervention and metastasis prevention (Fig. 3d).

The critical role of neural signaling in tumor metastasis is well established. A combined therapeutic strategy targeting neural pathways, angiogenesis, and anti-apoptotic mechanisms holds promise for more effective management of metastatic disease. In parallel, the development of novel diagnostic and therapeutic tools, particularly blood-based biomarkers, offers the potential to predict metastasis at earlier stages. Such advances will enable more personalized treatment approaches and timely interventions, ultimately expand therapeutic opportunities, and improve clinical outcomes for patients.

### Disrupting electrical hyperactivity for dual control of tumor growth

Neuronal electrical activity is essential for normal neurodevelopment. In primary brain tumors such as gliomas, tumor cells can structurally and electrically integrate into existing neural circuits. These tumor-neuron synapses enable glioma cells to exploit neuronal activity as a unique biological driver of malignancy [94, 200]. As a result, therapeutic interventions targeting electrical signaling represent a particularly promising and clinically valuable approach for treating tumors that rely on neural circuit integration.

Tumor cells from both adult and pediatric gliomas have been shown to form direct glutamatergic synapses with neurons [94, 95]. Paracrine glutamate release, via the x(c)(-)cystine-glutamate transporter system and other mechanisms, increases neuronal hyperexcitability, thereby accelerating tumor growth [201]. Calcium-permeable AMPAR-mediated excitatory postsynaptic currents depolarize glioma cells, and optogenetic depolarization directly enhances glioma proliferation [94]. Inhibition of AMPAR function using perampanel (PER), either pharmacologically or genetically, reduces glioma cell proliferation and invasion [94, 95]. BDNF enhances AMPAR trafficking to the glioma cell surface, amplifying glutamate-induced currents and calcium transients [112]. Genetic and pharmacologic inhibition of neurotrophic tyrosine receptor kinase 2 (TrkB) consistently reduces glioma responsiveness to glutamate, impairs synaptic connectivity between neurons and glioma cells, and suppresses glioma proliferation driven by neuronal activity [112] (Fig. 3e).

Glutamate is only one of several factors contributing to glioma-induced neuronal hyperactivity and tumor growth [95]. Recent studies have identified glioma-secreted phosphatidylinositol proteoglycan 3 as a driver of synaptogenesis and neuronal hyperexcitability, alongside evidence that gliomas can suppress inhibitory GABAergic currents in the surrounding electrical microenvironment [59, 152]. Another glioma-derived synaptogenic factor, thrombospondin-1, enhances functional neuronal connectivity between tumor and brain tissue [119]. This effect can be blocked by gabapentin, which inhibits thrombospondin binding to its neuronal receptor, α2δ-1 [202]. Although the precise biological contributions of each source of electrical activity remain incompletely understood, their role in glioma progression is increasingly evident. In neurofibromas originating from precancerous NF1-deficient SC precursors, sensory axon extension to NF1-mutant DRG neurons results in elevated action potential firing relative to wild-type controls, a process mediated by increased expression of collagen 1a2. In this context, pharmacological inhibition of neuronal excitability using tetrodotoxin significantly reduces neurofibroma growth in NF1-mutant mouse models in vivo [121].

The electrical integration of brain tumors into neuronal networks has prompted a re-evaluation of glioma-associated clinical manifestations, particularly seizures. While seizures are often attributed to mass effect or peritumoral edema, they may also arise directly from glutamate-mediated hyperexcitability driven by tumor-associated neural activity [201]. A growing body of clinical evidence shows that seizures are common among glioma patients, and epilepsy-induced neuronal activity may in turn promote glioma progression and malignancy [203–205]. Recent analyses of human glioma samples have identified hybrid cells, exhibiting properties of both oligodendrocyte precursor cells (OPCs) and GABAergic neurons (termed GABA-OPCs), that are capable of generating transient action potentials in isocitrate dehydrogenase (IDH)-mutant gliomas [206, 207]. Notably, up to 75% of patients with IDH-mutant gliomas experience tumor-related epilepsy, more than double the incidence observed in IDH-wild-type gliomas [208, 209]. These findings suggest that AMPAR-inhibiting antiepileptic agents, already approved for clinical use, may hold promise as dual-function therapies targeting both seizures and tumor growth. However, current data remain inconclusive, particularly for agents that modulate postsynaptic signaling. Further preclinical investigations and clinical trials are needed to evaluate the therapeutic potential of such agents in glioma treatment.

Although the mechanisms by which neural electrical activity influences cancer growth remain incompletely understood, emerging evidence supports a paradigm shift: tumors are not solely driven by cell-intrinsic processes but are profoundly shaped by their interactions with the surrounding microenvironment, including electrical networks within the nervous system.

### Harnessing sensory neuronal pathways for neuropathic pain control

Cancer-related neuropathic pain is a common and debilitating symptom that markedly reduces the quality of life in patients with cancer [210]. In malignancies characterized by dense neural infiltration or extensive bone metastasis, such as PC, PCa, and head and neck tumors, pain is frequently associated with pathological alterations in sensory nerves [42, 211, 212]. As such, targeting sensory pathways has emerged as a critical strategy for pain management. This section focuses on 4 major contributors to cancer pain: perineural inflammation, PNI, pain associated with metastatic spread, and chemotherapy-induced peripheral neuropathy (CIPN).

Perineural inflammation has emerged as a key contributor to cancer-related neuropathic pain [213]. Mast cells are found in close spatial proximity to nerve fibers, and bidirectional mast cell-neuron communication involves mast cell activation and degranulation [214, 215]. Notably, mast cells are highly enriched in intrapancreatic nerves, and their presence correlates with neuropathic abdominal pain in patients with PC [157]. Consistent with this, studies have reported increased mast cell-nerve proximity in PC cases exhibiting a higher degree of PNI [157, 216]. Targeting mast cell activity, particularly through inhibition of mast cell degranulation, represents a promising therapeutic strategy for alleviating neuropathic pain in PC.

PNI is closely associated with cancer-related pain. In cutaneous squamous cell carcinoma, tumor cells have been shown to disrupt the myelin sheath and induce degeneration of tumor-associated nerves [217]. In addition to malignant cells, non-malignant components of the TME, including TAMs and fibroblasts, actively contribute to tumor aggressiveness and pain. In sciatic nerve models, blockade of C-X-C motif chemokine ligand 2 and its receptor C-X-C chemokine receptor type 2 effectively reduced macrophage infiltration, mechanical allodynia, and spontaneous pain [118, 218, 219]. Notably, recent studies have uncovered a phenomenon of “macrophage-to-neuron-like cell transformation” [118, 220]. The neuronal transcription factor *Pou4f1* has been identified as a Smad3 target gene in bone marrow-derived macrophages stimulated by transforming growth factor-beta 1 (TGF-β1), implicating the TGF-β1/Smad3 axis in nociceptive signaling in chronic pancreatitis [141]. Inhibitors of Smad3 exhibit robust analgesic effects in animal models, and several downstream long non-coding RNAs regulated by Smad3 have been identified as potential therapeutic targets [118]. Furthermore, activation of SCs TRPA1 channels leads to the release of macrophage colony-stimulating factor, sustaining tissue-resident macrophage expansion and triggering oxidative stress, ultimately generating pain signals via neuronal TRPA1 activation [126].

Bone metastasis in PCa activates TRPV1⁺ sensory neurons within the bone via an acidic microenvironment, triggering the release of CGRP and SP, which mediate pain signaling [134, 145]. Neurotrophins such as NGF further activate TRPV1, leading to sensory neuronal membrane depolarization and enhanced SP/CGRP release [162]. In head and neck squamous cell carcinoma, lactic acid production via plasma membrane monocarboxylate transporter 4 contributes to acidification of the bone microenvironment, exacerbating nociceptive signaling [221]. The vacuolar proton pump inhibitor bafilomycin A1 has been shown to significantly alleviate bone pain by inhibiting extracellular acidification [140]. Similarly, a single injection of the selective TRPV1 antagonist SB366791 reduces sensory nerve excitation in preclinical models [123]. Aberrant osteoclast activation contributes to bone loss and promotes tumor progression and pain by disrupting bone homeostasis [222]. Sodium Danshensu alleviates cancer-induced bone pain by inhibiting osteoclast differentiation and reducing CGRP⁺ nerve sprouting [223]. CGRP antagonists such as CGRP_8-37_ effectively block neuropeptide-receptor interactions and attenuate neuropathic hyperalgesia in animal models [102] (Fig. 3f).

In addition to the neural alterations described above, peripheral nerves may also experience damage or dysfunction as a side effect of chemotherapy, a condition known as CIPN [224]. CIPN leads to persistent sensory disturbances, including neuropathic pain, paresthesia, and somatosensory loss, that severely diminish quality of life. Agents such as paclitaxel induce peripheral neuropathy through upregulation of lysophosphatidylcholine and galectin-3 [225, 226]. The PD-1/PD-L1 signaling axis has been shown to attenuate neuropathic pain in preclinical studies [227, 228]. Clinically, duloxetine is considered the first-line pharmacologic treatment for CIPN, while mirogabalin has demonstrated superior efficacy compared to pregabalin in recent trials [229, 230]. All trans retinoic acid alleviates chemotherapy-induced neuropathy by upregulating NGF and retinoic acid receptor β expressions [231]. In an orthotopic murine model of pancreatic cancer, pressurized regional intravascular delivery of chemotherapeutic agents reduced the required drug dose and mitigated CIPN-like behaviors, highlighting the potential of optimized drug delivery methods to minimize neurotoxicity while preserving anti-tumor efficacy [232].

In conclusion, targeting the sensory nervous system presents multiple promising avenues for managing cancer-related neuropathic pain. However, further research is required to translate preclinical insights into effective clinical interventions and to evaluate the synergistic potential of combined therapeutic strategies for addressing this complex and multifactorial symptom.

### Balancing the stress-HPA axis to regulate tumor cell responsiveness

In cancer research, elucidating the molecular, cellular, and tissue-level mechanisms of neural innervation in tumor initiation and progression remains essential [10]. However, it is equally critical to consider the human body as a complex, integrated system, and to incorporate systemic-level understanding into oncological frameworks. A growing body of evidence has demonstrated a strong correlation between stress-related psychosocial factors, such as stress-prone personality traits, maladaptive coping strategies, negative emotional responses, and diminished quality of life, and both cancer incidence and adverse clinical outcomes [233, 234]. Stress responses have been shown to profoundly influence tumor biology, including growth, metastasis, and therapeutic resistance, through intricate neuroendocrine signaling pathways [235, 236]. In particular, the HPA axis plays a central role in mediating the impact of chronic psychological stress on cancer progression [237].

Stress-related signaling through the HPA axis has been shown to impair DNA repair mechanisms and promote angiogenesis during periods of psychological distress or depression [238]. In murine models, anxiety-like behaviors induced by social defeat are associated with diminished antitumor efficacy, linked to elevated plasma corticosterone levels and upregulation of the glucocorticoid-responsive transcription factor TSC22 domain family member 3 (Tsc22d3) [133]. Glucocorticoids, a central component of the stress response, are key drivers of stress-induced metastasis and immune suppression [138, 146]. The synthetic GC dexamethasone (DEX) promotes breast cancer cell metastasis via activation of the PI3K pathway and upregulation of serum glucocorticoid-induced kinase 1 [144]. Stress also affects neutrophil function in the metastatic microenvironment. Stress-induced neutrophils can suppress T cell-mediated immune surveillance [149, 151], while glucocorticoid receptor (GR)-dependent formation of neutrophil extracellular traps (NETs) facilitates metastasis. NET formation may be attenuated by targeting GR signaling using cyclin-dependent kinase 4/6 (CDK4/6) inhibitors, cathepsin G inhibitors, or agents that suppress reactive oxygen species [107]. In stressed animals, NETs stimulate pulmonary fibroblasts to secrete fibronectin, promoting a prometastatic niche in the lungs [124, 128, 158]. Moreover, DEX induces resistance to chemotherapeutic agents such as doxorubicin and cisplatin in TNBC by upregulating the pro-survival transcription factor Kruppel-like factor 5 (KLF5) in a GR-dependent manner [144]. These adverse effects of stress or GC exposure on treatment response may be reversed using GR antagonists or by genetic ablation of Tsc22d3 in dendritic cells [116] (Fig. 3g). Additionally, stress-induced pro-inflammatory cytokines and neuroendocrine mediators, including catecholamines, histamine, 5-HT, and corticotropin-releasing hormone (CRH), can disrupt the gut-brain axis, leading to intestinal barrier dysfunction and dysbiosis, ultimately compromising antitumor immunity [239].

Emerging evidence indicates that behavioral interventions may exert beneficial effects on patients with cancer. For example, postoperative group-based cognitive behavioral stress management has been shown to reduce depressive symptoms in patients with early-stage breast cancer [240], and to lower both all-cause and breast cancer-specific mortality over 815 years of follow-up [241]. Emotional well-being appears to play a significant role in modulating tumor progression. Strategies aimed at improving patients’ psychological states, such as psychotherapy [242], meditation [243], yoga [244], regular physical activity [245], acupuncture [246], and the use of herbal compounds [247], may potentiate the effectiveness of cancer therapies. Nonetheless, the integration of such interventions into oncologic care requires rigorous multidisciplinary research to assess their feasibility, mechanistic underpinnings, and clinical efficacy.

Stress, a common physiological and psychological state, exerts multifaceted effects on tumor biology through complex neuroendocrine networks, thereby surpassing the limitations of conventional approaches that focus solely on tumor-intrinsic or microenvironmental factors. In tumor types that are particularly sensitive to emotional and hormonal modulation, such as certain breast cancers [248] and neuroendocrine tumors [132], the stress-responsive HPA axis frequently influences tumor evolution and represents a compelling target for systemic intervention. These findings underscore the critical importance of maintaining physiological and psychological homeostasis in efforts to prevent disease onset and enhance therapeutic efficacy.

### Decoding the role of brain-body communication on tumor progression

Cancer cells exploit not only local mechanisms to remodel the TME by co-opting and eliminating non-malignant cells, but also exploit bidirectional brain-body communication systems that normally maintain physiological homeostasis. By subverting these regulatory pathways, tumors facilitate growth, metastasis, and immune evasion [131]. A comprehensive understanding of these cross-system interactions presents a novel framework for developing therapeutic strategies against cancer.

The CNS plays a critical role in coordinating metabolic sensing across peripheral organs. Lateral orexigenic neurons in the hypothalamus have emerged as key regulators of tumor-induced metabolic dysregulation and the progression of cachexia [249, 250]. Recent studies indicate that targeted modulation of the area postrema (AP) neural network, particularly through blockade of pro-inflammatory signaling pathways such as interleukin (IL)-6 and suppression of AP hyperactivation, can restore systemic metabolic and immune balance, thereby mitigating cachexia progression [103, 104].

Bidirectional communication between the brain and the immune system plays a critical role in tumor immunosurveillance [251]. Tumor burden has been shown to elicit anxiety-like behaviors in mice, accompanied by the activation of CRH neurons in the central medial amygdala (CeM^CRH^) [156]. Simultaneously, newly formed sympathetic nerves have been detected within tumor tissues, forming polysynaptic connections with CeM^CRH^ neurons [84]. These neurons, in turn, engage catecholaminergic neurons in the lateral paragigantocellular nucleus (LPGi^CA^) [153, 155]. Activation of the CeM^CRH^-LPGi^CA^ axis significantly exacerbates anxiety-like behavior and promotes tumor growth [109]. Notably, pharmacological inhibition with alprazolam attenuated both neural activation and tumor progression by targeting this circuit [109].

Emerging evidence highlights the critical role of circadian regulatory machinery in tumor progression, mediated through genetic disruption or rhythm desynchronization [252]. Circadian fluctuations influence both tumor immunity and metabolism. Dendritic cells demonstrate diurnal migration into skin lymphatics, peaking during the daytime, thereby orchestrating circadian-dependent CD8^+^ T cell antitumor responses and modulating tumor growth kinetics according to implantation timing [165]. Chemogenetic activation of corticotropin-releasing hormone neurons in the paraventricular nucleus of the hypothalamus (PVN^CRH^) neurons during specific circadian phases restores glucocorticoid rhythmicity, suppresses tumor growth, and enhances intratumoral CD8^+^ T cell infiltration [166]. Additionally, circadian molecular clocks regulate key metabolic pathways, including glycolysis, mitochondrial function, lipogenesis, amino acid metabolism, and nucleotide synthesis. These findings support chronotherapeutic strategies that align anticancer interventions with biological rhythms to maximize therapeutic efficacy (Fig. 3h).

Sensory experience can influence tumor development through dedicated sensory neural circuits [253]. Chen et al. [120] demonstrated that olfactory input directly modulates glioma formation, wherein manipulation of olfactory receptor neuron activity significantly impacts tumor progression. Mechanistically, olfactory stimulation activates mitral/tufted (M/T) cells, which integrate sensory input and release insulin-like growth factor 1 (IGF1) in an activity-dependent manner. Tian et al. [127] further showed that genetic or pharmacological inhibition of the IGF1 receptor (IGF1R) suppresses the growth of a subset of glioma cells in both murine models and patient-derived samples. IGF1R inhibitors such as picropodophyllin (PPP, also known as AXL1717) effectively penetrate the blood-brain barrier and inhibit the growth of transplanted OPC-like tumors in vivo.

Such intervention strategies are particularly critical in malignancies characterized by both central regulation and peripheral immune-metabolic dysregulation. For example, cachexia syndrome associated with gastrointestinal tumors and cognitive decline observed in patients with brain metastases highlights the role of the brain-body axis not only in facilitating tumor adaptation, but also in driving systemic deterioration of the host [254]. These observations suggest that the nervous system may serve as a determinant of disease trajectory beyond the tumor itself. Accordingly, future cancer therapies may move beyond localized tumor eradication toward integrative, long-term strategies aimed at restoring physiological homeostasis and reinforcing systemic immune surveillance.

### Pioneering bioelectronic neuro-immunology for cancer treatment

Bioelectrical neuroimmunotherapy is an emerging therapeutic paradigm that seeks to modulate neuro-immune interactions through targeted bioelectrical stimulation, such as electric fields or pulsed currents, to enhance antitumor immune responses. This approach represents a promising frontier in precision oncology, offering a non-pharmacological strategy to reprogram the TME and augment immune-mediated tumor control [255].

Bioelectrical neuroimmunotherapy regulates immune responses by modulating neurotransmitter release (e.g., NE, ACh, etc.) through electrical stimulation (ES) of specific neural pathways [256]. Electroacupuncture (EA), particularly at acupoints adjacent to peripheral nerves such as ST36 (Zusanli), exhibits intensity-dependent immunomodulatory effects. High-intensity EA activates the sympathetic-splenic axis via NE release, leading to suppression of systemic inflammation, whereas low-intensity EA engages the vagus-adrenal axis to elicit anti-inflammatory effects [164]. The use of TRPV1 agonists such as capsaicin desensitizes TRPV1-expressing sensory neurons, thereby preventing vagus-adrenal axis activation by EA at ST36 [164]. A recent study has identified PROKR2^Cre^ sensory neurons, which innervate the deep fascia (periosteum) of the hindlimb, as essential mediators of EA-induced adrenal catecholamine release and the ensuing immunosuppressive response [125]. These adrenal catecholamines (NE and epinephrine) suppress systemic inflammation by inhibiting the release of pro-inflammatory cytokines [125]. Additionally, NE stimulates splenic lymphocytes to produce ACh, which acts on spleen-resident macrophages to attenuate their inflammatory activity [125] (Fig. 3i). VN stimulation also enhances T cell immunity by reducing IL-6, TNF-α, and IL-1β production via α7 nicotinic ACh receptor signaling [257]. Low-intensity stimulation of the Sanli point on the forelimb has likewise been shown to activate the vagus-adrenal pathway and confer anti-inflammatory benefits. However, high-intensity EA at ST36 in lipopolysaccharide-stimulated mouse models was associated with exacerbated inflammation, highlighting the importance of both stimulation intensity and physiological context in determining therapeutic outcomes [258, 259]. Furthermore, direct ES of the sciatic nerve has been shown to activate natural killer cells and inhibit tumor growth, synergizing with anti-PD-1 immunotherapy via interferon-γ-driven PD-L1 upregulation [260].

Bioelectrical stimulation also modulates immunosuppressive pathways within the TME. Trefoil factor 2 (TFF2), an anti-inflammatory peptide predominantly secreted by the spleen, has been shown to inhibit the proliferation and differentiation of myeloid progenitor cells into MDSCs, thereby mitigating immunosuppression [261]. Notably, elevated TFF2 expression plays a critical role in restraining tumor progression. VN stimulation enhances splenic TFF2 expression, contributing to the suppression of MDSC accumulation. In contrast, bilateral subdiaphragmatic vagotomy abolishes this response, resulting in increased MDSC levels and enhanced colonic tumorigenesis [111]. Supporting this neuro-immune axis, Erin et al. [159] demonstrated that CNI-1493 (Semapimod), a small-molecule inhibitor known to activate vagal signaling, significantly reduced breast cancer metastasis in preclinical models. These findings underscore the therapeutic potential of neuromodulation in reshaping the immunosuppressive landscape of the TME and attenuating tumor progression.

This strategy is particularly well-suited for malignancies that retain immunomodulatory potential and are amenable to neuroregulatory intervention, such as subsets of colorectal and breast cancers. In these tumor types, the neuro-immune network within the TME remains sufficiently plastic, offering a therapeutic window for bioelectrical modulation [262]. Importantly, the various elements of bioelectrical neuro-immunity form a highly interconnected and dynamic system. A comprehensive analysis of this network not only deepens our understanding of the molecular mechanisms driving disease progression but also lays a solid theoretical foundation for the development of novel diagnostic tools, therapeutic approaches, and preventive strategies in cancer care.

Despite mounting evidence implicating neural-tumor interactions in cancer progression [84, 85, 251], the efficacy of neuro-targeted strategies remains highly context-dependent. This heterogeneity largely reflects the variable extent of neural infiltration and functional innervation across cancer types. Tumors such as gliomas [58, 59], PDAC [18], and PCa [78] exhibit pronounced PNI and neural integration, rendering them particularly susceptible to neuromodulatory interventions. By contrast, malignancies characterized by low nerve density or limited neuro-immune crosstalk, such as certain hematologic cancers [263], are less likely to benefit from such approaches, underscoring the need for tumor-type-specific strategies.

While the pervasive influence of the nervous system on cancer hallmarks offers a unifying framework for neuro-targeted therapies, no universally effective paradigm has emerged. Tumor heterogeneity remains a central obstacle to the development of broadly applicable strategies [264]. Distinct malignancies exhibit divergent biological traits, microenvironmental architectures, and neural engagement patterns. For instance, sensory denervation suppresses PC growth but paradoxically accelerates melanoma progression [172, 173]; synaptic integration and neuronal hyperexcitability are prominent in IDH-mutant gliomas yet rarely observed in extracranial solid tumors [208, 209]; and interventions targeting the stress-hypothalamic axis [156] or the gut-brain axis [265] yield variable outcomes depending on host physiology and tumor burden. The therapeutic efficacy of agents such as β-AR blockers [266], AMPAR antagonists [94], and bioelectrical stimulation [164] hinges on the alignment between a tumor’s neural dependency and its anatomical and functional context.

Tumor heterogeneity also shapes clinical priorities, with each cancer type presenting distinct therapeutic imperatives driven by its dominant biological features. In high-grade gliomas, the clinical focus lies in curbing rapid proliferation and mitigating mass effect [94]. While in PC and bone metastases, alleviating neuropathic pain is essential to improving patient quality of life [145, 157]. Thus, the rational design of neuro-targeted interventions must align with both the tumor’s biological behavior and its associated clinical manifestations, enabling precision strategies tailored to context-specific needs.

In light of these considerations, future efforts in the field should emphasize the development of stratified intervention frameworks over universal therapeutic models. While targeting tumor innervation offers a compelling therapeutic avenue, its clinical utility is inherently context-dependent. The success of such strategies will rely on robust patient stratification, precise elucidation of tumor-specific neural dependencies, and their rational integration with existing modalities, including immunotherapy, molecularly targeted agents, and systemic treatments. Ultimately, a tailored, mechanistically informed approach is essential to address the diverse neurobiological architectures across tumor types.

## Strategic approaches to treatment

At this critical juncture in cancer prevention and control, the targeted disruption of tumor innervation has emerged as a strategically promising and potentially transformative therapeutic avenue. The following sections systematically delineate a range of neuro-targeted intervention strategies, encompassing, but not limited to, innovative translational applications via drug repurposing, nanotechnology-enabled delivery systems, gene-editing-based precision interventions, and synergistic combination regimens designed to enhance clinical efficacy through complementary mechanisms.

### Drug repurposing

Our previous studies have highlighted the growing appeal of drug repurposing in oncology, owing to several key advantages: 1) reduced development time and cost; (2) lower risk of clinical failure due to established safety, toxicity, and pharmacokinetic profiles; and 3) expedited clinical translation once antitumor efficacy is confirmed [267, 268]. In the present work, we focus on the therapeutic application of neuroactive agents, including antipsychotics, antidepressants or anxiolytic drugs, antiepileptics, β-AR blockers, and neurodegenerative disease medications, in cancer treatment. Specifically, we summarize how repurposed neuroactive compounds can mitigate chronic stress, restore circadian rhythm, modulate tumor neuroelectric activity, and target the neuro-immune-tumor axis.

#### Antipsychotic pharmaceutical drugs: olanzapine (OLZ)

**Fig. 4:**
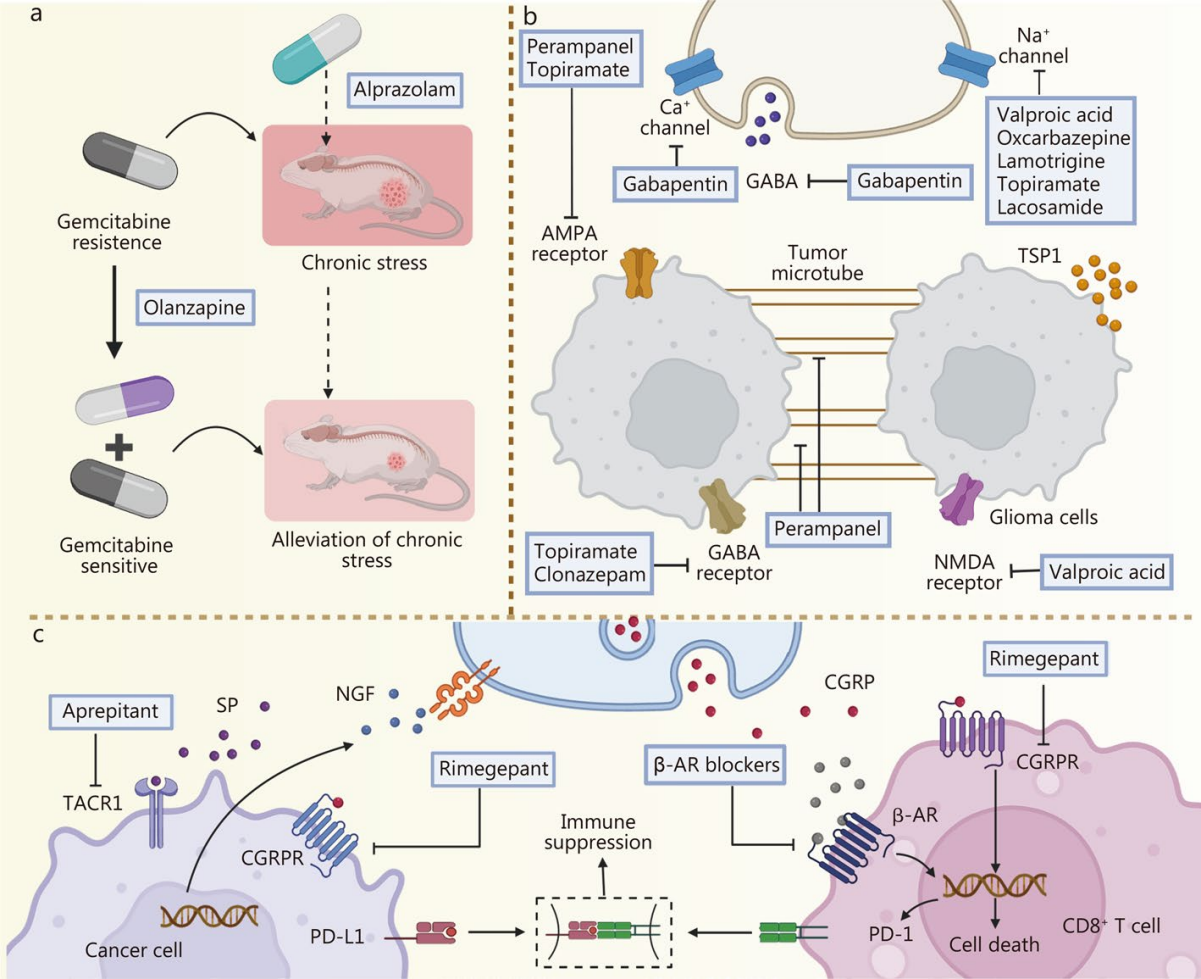
Drug repurposing to target tumor innervation.** a** Systemic regulation of chronic stress and circadian rhythm, by directly or indirectly alleviating chronic stress and regulating circadian rhythm, reversing tumor drug resistance, and restoring chemotherapy sensitivity. **b** Target tumor nerve electrical activity, inhibit tumor invasion driven by abnormal electrical signals by antagonizing GABA and glutamate signals, and regulating ion channels. **c** Coordinated intervention of neuro-immune-tumor axis to overcome immunosuppression by blocking T cell depletion and functional inhibition mediated by NGF, NE, and CGRP. This figure was created using BioRender (https://biorender.com/). GABA γ-aminobutyric acid, NGF nerve growth factor, CGRP calcitonin gene-related peptide, CGRPR calcitonin gene-related peptide receptor, AMPA α-amino-3-hydroxy-5-methyl-4-isoxazolepropionic acid, TSP1 thrombospondin 1, NMDA N-methyl-d-aspartic acid, TACR1 tachykinin receptor 1, SP substance P, PD-L1 programmed death-ligand 1, β-AR β-adrenergic receptor, PD-1 programmed death-1

OLZ, a widely used antipsychotic agent, has been shown to modulate neural activity in brain regions implicated in arousal and reward processing [269]. Specifically, OLZ increases c-Fos immunoreactivity in the medial prefrontal cortex and nucleus accumbens shell, while concurrently reducing c-Fos expression in other regions, leading to attenuated NE release [270]. This modulation of NE signaling has been linked to suppression of circadian locomotor output cycles, kaput expression, and downregulation of circadian rhythm pathways [271], thereby reversing chronic stress-induced chemoresistance to gemcitabine [272]. OLZ also sensitizes lung and pancreatic cancer stem-like cells to chemotherapeutics such as 5-fluorouracil, gemcitabine, and cisplatin by downregulating survivin, a key mediator of multidrug resistance [273]. Moreover, OLZ induces autophagy in glioma cells by inhibiting NF-κB activation through suppression of p65 nuclear translocation, thereby enhancing autophagic flux and autophagosome formation [274] (Fig. 4a).

#### Antidepressant drugs: vortioxetine, phenelzine, and ketamine

Vortioxetine, traditionally prescribed as an antidepressant, has recently garnered attention for its potential anti-tumor effects in GBM [275]. In vitro studies have demonstrated that vortioxetine treatment significantly downregulates proliferation-associated markers, arrests cell cycle progression, and upregulates apoptosis-related proteins in GBM cells [200, 275, 276]. Histopathological analyses of tumor tissues further revealed reduced tumor cell density, altered cellular morphology, and inhibited tumor angiogenesis in the vortioxetine-treated group [277, 278]. Mechanistically, vortioxetine has been shown to upregulate genes such as activator protein 1 (*AP-1*) and B-cell translocation gene (*BTG*), which are involved in apoptosis induction, cell cycle regulation, and tumor suppression [279, 280]. Although these findings are promising, the application of vortioxetine in GBM remains at the preclinical research stage. In addition to vortioxetine, other neuroactive agents also exhibit potential antitumor effects. Phenelzine, a monoamine oxidase A inhibitor used in depression, has been found to enhance anti-tumor immunity and synergize with anti-PD-1 therapy in wild-type mice. Mechanistically, monoamine oxidase A suppresses T cell function by disrupting serotonin-mediated autocrine signaling in tumor-infiltrating T cells [281]. Furthermore, ketamine, a dissociative anesthetic known for its rapid antidepressant effects, has been shown to elevate BDNF levels and enhance synaptic plasticity in preclinical depression models [282, 283]. Ketamine also activates the α7 nicotinic ACh receptor, inhibits pro-inflammatory signaling cascades, and reduces neuroinflammation and apoptosis [284], suggesting potential utility in managing cancer-related neuropathic pain.

#### Antianxiety drugs: alprazolam

The anxiolytic agent alprazolam also exerts significant regulatory effects on tumor progression through its neuromodulatory properties. Notably, alprazolam has been shown to suppress the activity of CeM^CRH^ and LPGi^CA^, thereby alleviating cancer-induced anxiety, inhibiting sympathetic nervous system activity, and enhancing anti-tumor immune responses [109]. Activation of intratumoral sympathetic nerves has been associated with impaired M1 macrophage polarization and a decreased M1/M2 macrophage ratio, contributing to a more immunosuppressive TME [109, 285, 286]. Treatment of tumor-bearing mice with alprazolam led to a marked reduction in anxiety-like behaviors, significant tumor growth inhibition, decreased tumor weight, reduced bioluminescent (luciferase) signals, and lower intratumoral NE levels [109]. Moreover, alprazolam treatment resulted in enhanced immune cell infiltration within the TME. This included an increase in CD45⁺ leukocytes, CD4^+^ T cells, and CD8^+^ cytotoxic T lymphocytes, alongside a notable reduction in regulatory Tregs, as well as exhausted CD4⁺PD-1⁺ and CD8⁺PD-1⁺ T cell populations [287]. These immunomodulatory effects collectively underscore alprazolam’s potential in mitigating stress-induced tumor immune evasion and offer mechanistic insight into its adjunctive therapeutic utility in oncology [109] (Fig. 4a).

#### Antiepileptic drugs (AEDs): levetiracetam (LEV), lamotrigine (LTG), and PER

The repurposing of AEDs for oncological applications has shown emerging potential but remains limited by key translational challenges. LEV has been observed to reduce GABAergic postsynaptic currents in diffuse intrinsic pontine glioma (DIPG) cells and suppress tumor proliferation in patient-derived DIPG xenograft models [288]. While retrospective clinical studies have suggested a survival benefit with LEV use, particularly among patients with O6-methylguanine-DNA methyltransferase promoter methylation, this effect has not been consistently corroborated by prospective clinical trials, underscoring the need for further investigation into the relationship between LEV administration and survival outcomes [289, 290]. Similarly, in neurofibromatosis type 1 models, LTG has demonstrated the capacity to decelerate optic pathway glioma growth by targeting hyperpolarization-activated cyclic nucleotide-gated channels, thereby restoring neuronal excitability and attenuating tumor proliferation and progression in vivo [121]. PER, a selective AMPAR antagonist, has shown robust anti-proliferative effects in glioma-bearing mice, extending survival and inhibiting tumor invasion. These effects were linked to PER’s ability to disrupt neuronal activity-induced TMs branching and elongation, which are critical for glioma network integration and progression [112, 276]. Other AEDs, including topiramate, clonazepam, and oxcarbazepine, have also exhibited preliminary antitumor properties in preclinical settings. However, their precise mechanisms of action and therapeutic efficacy require further elucidation in well-controlled studies [290] (Fig. 4b).

#### β-AR blockers: propranolol

Propranolol, a non-selective β-AR blocker widely prescribed for cardiovascular diseases, has demonstrated multifaceted antitumor effects in infantile hemangiomas and breast cancer [291]. By antagonizing β2-AR, propranolol induces vasoconstriction and reduces tumor blood flow, thereby alleviating IH symptoms [292]. In a randomized phase II trial, Hiller et al. [293] reported that oral propranolol administration 1 week before surgery significantly lowered biomarkers associated with breast cancer metastasis, alongside marked reductions in pro-tumor transcription factors such as NF-κB and AP-1. Furthermore, propranolol mitigates stress-induced distant metastasis in orthotopic breast cancer xenografts by inhibiting TAM infiltration [116]. In CRC models, propranolol activates CD8^+^ T cells and suppresses tumor progression through inhibition of the protein kinase B/MAPK signaling pathway [294]. Additionally, nebivolol, a selective β1-AR blocker, impairs cancer cell oxidative phosphorylation by simultaneously inhibiting mitochondrial complex I and ATP synthase, while also suppressing tumor angiogenesis via inhibition of endothelial cell proliferation [266] (Fig. 4c).

#### Central antiemetic drugs: aprepitant

SP interacts with the tumor acinar peptide receptor 1 (TACR1), inducing apoptosis in a subset of TACR1-overexpressing cancer cells [295]. The release of single-stranded RNA (ssRNA) from these dying cells subsequently activates adjacent tumor-expressed Toll-like receptor 7 (TLR7), triggering an atypical pro-metastatic gene expression program involving the PI3K-Akt signaling pathway [295]. Notably, this SP/ssRNA-TLR7-induced gene signature correlates with poorer survival outcomes in breast cancer patients [295]. The TACR1 antagonist aprepitant effectively inhibits breast cancer cell invasiveness and metastatic progression, while also modulating the TME by disrupting neuro-cancer cell interactions [295]. These findings highlight aprepitant’s potential as an adjuvant therapy, warranting further clinical investigation, particularly in combination with established treatment regimens to improve therapeutic efficacy (Fig. 4c).

#### Antimigraine drugs: rimegepant

Rimegepant, a clinically approved treatment for acute migraines, has shown promise in disrupting tumor-nerve crosstalk in oral squamous cell carcinoma, which is characterized by glucose deprivation [296, 297]. Nutrient scarcity triggers ROS-mediated activation of c-Jun in cancer cells, leading to secretion of NGF that primes nociceptive nerves for CGRP production [298, 299]. CGRP, in turn, induces cytoprotective autophagy in cancer cells and promotes exhaustion of CD8^+^ T cells, thereby impairing their antitumor cytotoxicity against melanoma cells [99]. Importantly, CGRP-induced autophagy can be attenuated by the CGRP receptor antagonist rimegepant [300]. When combined with agents such as lonidamine, anlotinib, or bevacizumab to disrupt this feedback loop, rimegepant enhances antitumor efficacy at doses otherwise limited in monotherapy [300]. These findings warrant further clinical investigation to optimize combination regimens targeting neurogenic pathways in cancer (Fig. 4c).

Despite the promising potential of neuroactive drug repurposing in oncology, certain regulatory agents targeting the nervous system may paradoxically accelerate tumor progression. For instance, morphine-induced upregulation of the μ-opioid receptor has been shown to promote proliferation in CRC [301] and NSCLC cells [302]. Opioids also enhance angiogenesis by elevating intracellular VEGF levels [303]. Several preclinical studies (Table 2) [109, 112, 121, 266, 271, 276, 279–281, 288, 293–300] and clinical trials (Table 3) of drug repurposing targeting the innervated TME have been conducted or are currently underway.

**Table 2:** Preclinical trials of drug repurposing to target the innervated tumor microenvironment

Representative drugs	Tumor type	Therapeutic avenue	Mechanism and target	Main research achievements	Reference
Olanzapine (OLZ)	Lung cancer	Chronic stress	NE inhibitor	OLZ suppresses the mPFC-NE-CLOCK axis, thereby ameliorating tumorigenesis and chemoresistance	[271]
Vortioxetine	Melanoma	Chronic stress	Upregulate the expression of genes such as *AP-1* and *BTG*	Vortexinostat treatment reduced tumor cell density, altered cell morphology, and inhibited tumor angiogenesis	[279, 280]
Phenelzine	Glioblastoma	Chronic stress	5-HT inhibitor	MAOI treatment significantly inhibits tumor growth in preclinical mouse syngeneic and human xenograft models in a T cell-dependent manner	[281]
Alprazolam	Breast cancer	Chronic stress	NE inhibitor	Alprazolam significantly reduced CeM^CRH^ and LPGi^CA^ neuron activity, cancer-induced anxiety, and slowed tumor progression in tumor-bearing mice	[109]
Levetiracetam (LEV)	Glioma	Tumor neuroelectric activity	GABA inhibitor	LEV reduces GABAergic postsynaptic currents in DIPG cells and attenuates tumor proliferation in patient-derived xenografts	[288]
Lamotrigine (LTG)	Optic glioma	Tumor neuroelectric activity	Hyperpolarization-activated HCN channel	LTG reverses neuronal hyperexcitability, reduces optic nerve proliferation, and slows optic glioma progression in vivo	[121]
Perampanel (PER)	Glioma	Tumor neuroelectric activity	AMPAR antagonist	PER treatment conferred significant antiproliferative effects and survival benefits in glioma-xenografted mice and inhibited tumor invasion in vivo	[112, 276]
Propranolol	Breast cancer	Neuro-immune-tumor axis	β-AR blocker	Propranolol can reduce distant metastasis induced by stress in orthotopic xenograft tumors by inhibiting tumor-associated macrophage infiltration in breast cancer	[116, 293]
	Colon cancer	Neuro-immune-tumor axis	β-AR blocker	Propranolol downregulates p-Akt/p-ERK/p-MEK expression in tumor tissue and significantly increases the frequency of tumor-infiltrating CD8^+^ T cells	[294]
Nebivolol	Colon cancer and breast cancer	Neuro-immune-tumor axis	β-AR blocker	Nebivolol specifically hinders oxidative phosphorylation in cancer cells by inhibiting complex I and ATP synthase activities and arrests tumor angiogenesis	[266]
Aripitant	Breast cancer	Neuro-immune-tumor axis	SP/TACR1 antagonist	Aprepitant inhibits breast cancer cell invasiveness and metastatic progression and regulates the TME in both 3D culture and mouse models	[295]
Rimegepant	Squamous cell carcinoma	Neuro-immune-tumor axis	CGRP receptor antagonist	Cancer cells secrete NGF via the ROS/c-Jun pathway in glucose-deficient environments, activating pain nerves to release CGRP. CGRP induces protective autophagy in cancer cells, promoting survival, which can be blocked by the CGRP receptor antagonist Rimegepant	[296–300]

*5-HT* serotonin (5-Hydroxytryptamine), *AP-1* activator protein 1, *BTG* B-cell translocation gene, *CeMRH* central medial region of the right hypothalamus, *c-Jun* cyclin D-dependent kinase 4 inhibitor, *CLOCK* circadian locomotor output cycles kaput, *DIPG* diffuse intrinsic pontine glioma, *LPGiA* lateral paragigantocellular nucleus of the amygdala, *HCN* hyperpolarization-activated cyclic nucleotide-gated, *MAOI* monoamine oxidase inhibitor, *mPFC* medial prefrontal cortex, *ROS* reactive oxygen species, *SP/TACR1* substance p/neurokinin-1 receptor, *TME* tumor microenvironment, *β-AR* β-adrenergic receptor

**Table 3:** Clinical trials of drug repurposing to target the innervated tumor microenvironment

Cancer type	Therapy	Target	Trial start /end times	Phase	Actual or target accrual	Primary endpoint	Status	Outcome	Clinical trial number
Glioblastoma	Gabapentin sulfasalazine memantine	Glutamate inhibitors	2023-01**/**ongoing	Phase Ib/II	120	PFS-6	Ongoing	Not reported	NCT05664464
Glioblastoma	Perampanel	AMPA	2023-10**/**ongoing	Phase II	66	Neuron-tumor synaptic connectivity in glioblastoma tissue and changes in tumor growth rate	Ongoing	Not reported	EUCT2023-503938-52-00 30.11.2023
CIPN	ATX01 (topical amitriptyline hydrochloride)	Reuptake of norepinephrine and serotonin in the CNS	2022-07**/**ongoing	Phase II	240	Change from baseline to week 12 in the weekly mean of the daily NPRS score assessing average pain intensity in target study extremities related to CIPN in the past 24 h	Ongoing	Not reported	EUCTR2022-000435-23-CZ
Bladder cancer	Propranolol, pembrolizumab	β-adrenergic antagonist	2021-05**/**ongoing	Phase II	25	Objective response rate	Ongoing	Not reported	NCT04848519
Melanoma	Propranolol, pembrolizumab	β-adrenergic antagonist	2018-01**/**ongoing	Phase I	9	Safety	Ongoing	Not reported	NCT03384836
Colorectal cancer	Propranolol, COX2 inhibitor	β-adrenergic antagonist	2010-01**/**2017-01	Phase III	34	3-year recurrence	Completed	No difference in recurrence	NCT00888797
Infantile hemangioma	Nadolol	β-adrenergic antagonist	2015-09**/**2020-06	Phase II	74	Safety and noninferiority	Completed	No difference in efficacy or safety	NCT02505971
Infantile hemangioma	Propranolol, COX2 inhibitor	β-adrenergic antagonist	2010-01**/**2013-11	Phase II/III	456	Long-term efficacy and safety	Completed	Higher frequency of successful treatment than with a placebo	NCT01056341
Breast cancer	Propranolol, COX2 inhibitor	β-adrenergic antagonist	2014-06**/**2016-01	Phase II	38	Pro-metastatic cell markers	Completed	Decreased pro-metastatic and invasive markers	NCT00502684

*ATPX01* amantadine hydrochloride, *CIPN* chemotherapy-induced peripheral neuropathy, *CNS* central nervous system, *COX2* cyclooxygenase-2, *EUCTR* European clinical trials register, *NCT* national clinical trial, *NPRS* numeric pain rating scale, *PFS-6* progression-free survival at 6 months, *EGLN3* Egl-9 family hypoxia inducible factor 3, *CeM*^*CRH*^ CRH neurons in the central medial amygdala, *LPGi*^*CA*^ catecholaminergic neurons in the lateral paragigantocellular nucleus, *PVN*^*CRH*^ corticotropin-releasing hormone neurons in the paraventricular nucleus of the hypothalamus, *Akt* protein kinase B, *GSK3β* glycogen synthase kinase 3β, *CREB* cAMP response element-binding protein

Nevertheless, the clinical translation of drug repurposing faces several significant challenges. Firstly, while some small-scale retrospective and prospective studies report favorable outcomes, these are often limited by small sample sizes, heterogeneous experimental designs, and a lack of robust evidence from large randomized controlled trials, constraining their broader applicability. Secondly, drug resistance remains a major obstacle; tumors initially responsive to repurposed agents frequently develop adaptive resistance over time. Finally, the pharmacokinetic profiles of existing drugs may not optimally suit new oncologic indications, necessitating thorough re-evaluation of dosing regimens and delivery methods [304]. Therefore, despite offering a novel therapeutic avenue, the clinical efficacy and feasibility of drug repurposing require further rigorous validation and optimization.

### Targeted therapy

#### Innervated-nanomedicine

**Fig. 5:**
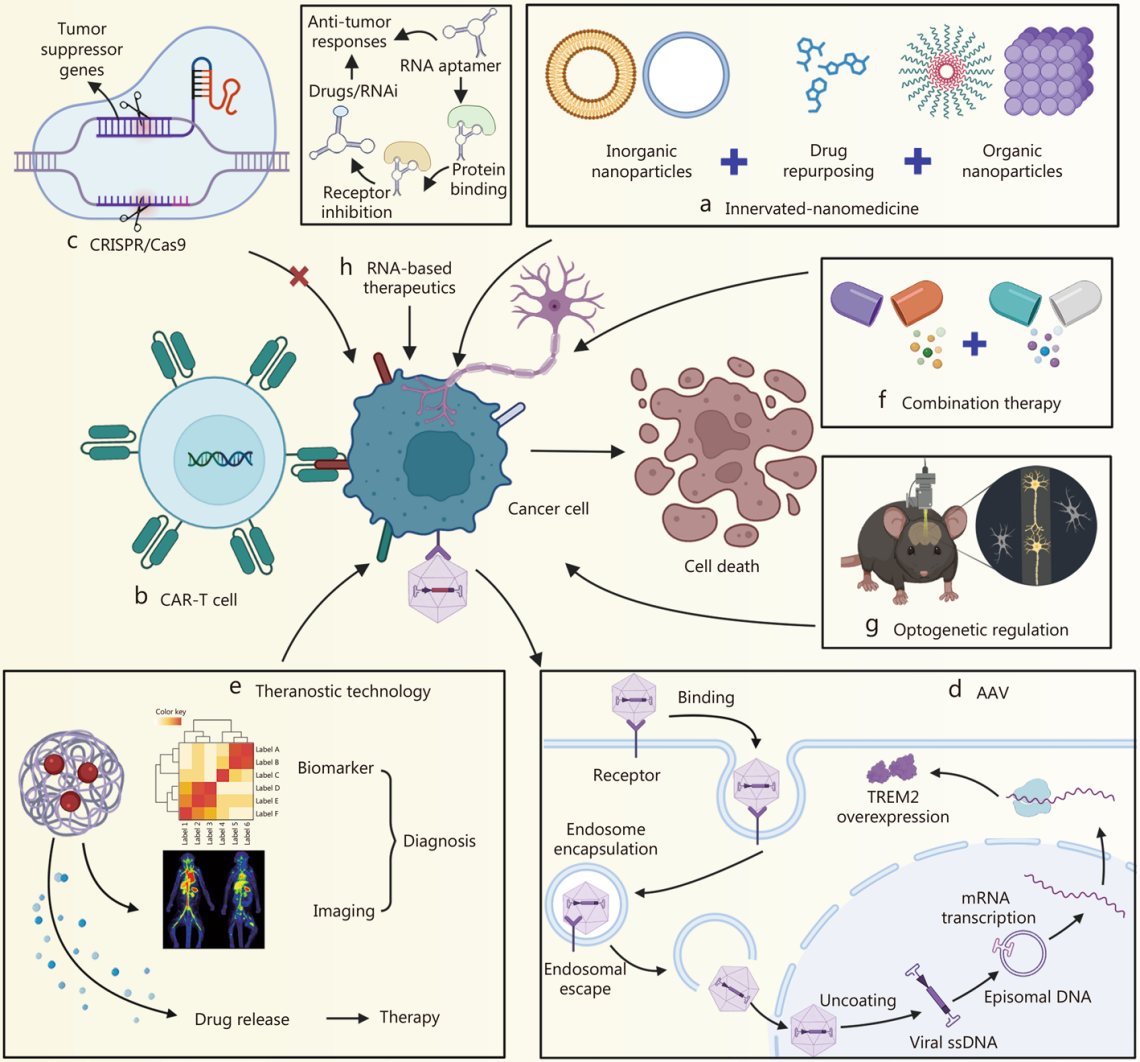
Strategic approaches to treatment. **a** Neuro-targeting nanomedicine delivers therapeutic drugs through the blood-nerve barrier through functional modification. **b** CAR-T cell therapy engineered T cells to target neuro-associated antigens. **c** CRISPR/Cas9 gene editing accurately knocked out the gene promoting nerve infiltration. **d** AAV therapy delivers anti-tumor growth genes or drugs. **e** Integrated diagnosis and treatment technology integrates imaging and treatment, with real-time monitoring of neuro-tumor interaction. **f** Combination therapy with nerve blockers and immune checkpoint inhibitors to enhance the efficacy. **g** Optogenetic regulation uses photosensitive proteins to precisely manipulate the activity of tumor-associated neurons. **h** RNA therapies (such as mRNA vaccines or drugs) regulate neural signaling pathways. This figure was created using BioRender (https://biorender.com/). CAR chimeric antigen receptor, CRISPR clustered regularly interspaced short palindromic repeats, Cas9 CRISPR-associated protein 9, AAV adeno-associated virus, TREM2 triggering receptor expressed on myeloid cells 2, ssDNA single-stranded DNA

Significant advances have been achieved in applying nanotechnology to targeted cancer therapy. The rapid evolution of nanomedicine has expanded the repertoire of materials available for NP design, with an increasing diversity and number of NPs under investigation. These primarily encompass inorganic and organic NPs, each offering unique properties for drug delivery and therapeutic applications [305] (Fig. 5a).

Ferritin nanoparticles (FtNPs) have emerged as a versatile tumor-targeted delivery platform due to their inherent biocompatibility and safety profile. For example, local administration of FtNPs loaded with atropine effectively blocks muscarinic signaling within the TME, suppressing pancreatic tumor growth and reducing neurogenesis [306]. Similarly, iron oxide NPs conjugated with polyethylene glycol and dual-targeting ligands, scFvCD44v6 and scAbNMDAR2B, simultaneously engage CD44 variant isoform 6 and the NMDAR subunit 2B, demonstrating potent inhibition of PNI in PDAC cells [114]. Mg/Al layered double hydroxide (Mg/Al-LDH) nanoshells have been engineered to alleviate cancer-induced nociceptive abnormalities by scavenging pain-inducing protons (H⁺), facilitated by internal loading of the AZ-23 antagonist and surface decoration with alendronate. This system also inhibits neurogenesis via blockade of the NGF/TrkA pathway [307]. Sun et al. [308] developed a composite nanoplatform comprising manganese dioxide (MnO₂), bovine serum albumin, and polydopamine, which reduces inflammatory pain by ablating tumor-innervated sensory nerve fibers through TRPV1 channels. Moreover, NP-encapsulated AZ3451 (PAMAM-Chol-AZ NPs) efficiently inhibits protease-activated receptor 2 activation, a key driver of nociceptor sensitization in the TME, at both plasma membrane and endosomal sites, substantially mitigating nociceptive responses compared to free drug administration [309]. In bone regeneration and cancer therapy, bioceramic tricalcium phosphate scaffolds encapsulated with polylactic acid/germanium selenium (PLA/GeSe) nanofiber membranes promote early innervation, bone repair, and reversal of osteosarcoma-induced bone defects [310]. An engineered H-MnCa/3MA-ALD NP system further suppresses tumor growth and cancer-induced bone pain by downregulating nerve pain mediators such as NGF [311].

Outer membrane vesicles (OMVs) derived from *Escherichia coli* Nissle 1917 have been engineered to bind the neurobinding peptide NP41 and loaded with the Trk inhibitor larotinib, forming Lar@NP-OMVs [312]. This platform effectively disrupts neurotrophic factor-Trk signaling, reducing neurite outgrowth and inducing repolarization of TAMs from an M2- to an M1-like phenotype, which contributes to nerve damage and tumor inhibition [312]. Liang et al. [313] developed a neurotransmitter-mimicking nanovesicle (PMVS-P), based on platelet membrane-derived vesicles, loaded with the glioma stem cell-targeting agent salinomycin and coated with polydopamine. PMVS-P specifically targets surgical incision sites by recognizing the dopamine D2 receptor, thereby inhibiting GBM recurrence. The P@P emulsion, designed as an siRNA delivery system, enhances tumor penetration and significantly suppresses PC progression via targeted knockdown of NGF expression [314]. Polyethylene glycol-modified liposomal bupivacaine improves drug uptake following intravenous administration and selectively accumulates in tumor-associated neurons to inhibit tumor growth [315, 316]. He et al. [317] introduced a biomimetic NP tumor innervation disruptor consisting of hollow MnO₂ nanocarriers cloaked with tumor cell membranes, encapsulating lidocaine to achieve effective antitumor activity. Furthermore, tumor membrane-encapsulated biomimetic nanovaccines co-delivering β-AR receptor inhibitors (e.g., propranolol) and immune adjuvants (e.g., CpG) promote dendritic cell maturation, activate CD8^+^ T cell effector responses, and markedly reduce immunosuppressive cell populations within the TME, thereby enhancing antitumor immunity [318, 319].

The application of nanotechnology in tumor therapy, particularly for targeted delivery and modulation of tumor innervation, holds substantial promise. Future research will prioritize not only improving the biocompatibility and therapeutic efficacy of nanocarriers but also optimizing multifunctional platforms capable of delivering precise, tailored treatments across diverse tumor types and clinical scenarios.

#### Chimeric antigen receptor T (CAR-T) cell

CAR-T, engineered as living therapeutics, employ synthetic receptors to directly recognize tumor surface antigens, enabling targeted cytotoxicity independent of traditional antigen presentation pathways [320]. Conventional CAR-T designs utilize single-chain variable fragments (scFvs) as antigen recognition domains. Recent advances have demonstrated that CAR-T cells engineered to secrete NGF-specific scFvs induce sympathetic denervation within the TME, concurrently reprogramming macrophages toward an immunostimulatory phenotype with enhanced phagocytic activity [321]. This strategy also effectively inhibits terminal exhaustion of tumor-infiltrating T cells, mitigating immunosuppression within the TME and potentiating CAR-T cell antitumor efficacy [321].

Alternatively, natural receptor ligands can serve as substitutes for scFvs in CAR-T cell design, offering enhanced specificity and sensitivity with minimal immunogenicity and reduced off-target toxicity [322, 323]. BDNF and neurotrophin 4 (NTF4) are endogenous ligands for TrkB, whose binding activates downstream signaling pathways implicated in diverse tumorigenic processes [324]. Liang et al. [325] engineered TrkB-targeted CAR-T cells utilizing BDNF and NTF4 as recognition domains, demonstrating that both BDNF-CAR-T and NTF4-CAR-T effectively target and suppress TrkB-positive solid tumors.

Protein tyrosine phosphatase 4A2 (PTP4A2) has been identified as a critical regulator of self-renewal, proliferation, and tumorigenicity in recurrent GBM. Genetic ablation or pharmacological inhibition of PTP4A2 suppresses tumor growth by promoting dephosphorylation of the roundabout guidance receptor 1 (ROBO1) and its downstream effectors [326]. Building on this, Chokshi et al. [326] engineered second-generation CAR-T cells targeting ROBO1, demonstrating significant extension of median survival in recurrent GBM xenograft models. Moreover, in cell-derived xenograft models of adult lung-to-brain metastases and pediatric recurrent medulloblastoma, ROBO1 CAR-T therapy achieved clearance of 50–100% of tumor cells (Fig. 5b).

Despite promising advances, the clinical application of CAR-T cells in solid tumors faces significant challenges, notably limited tumor infiltration and antigenic heterogeneity. Future research must focus on optimizing cell manufacturing and infusion protocols to enhance CAR-T cell persistence, trafficking, and efficacy within the complex TME.

#### Clusters of regularly interspaced short palindromic repeats (CRISPR)/CRISPR-associated protein (Cas)9 gene editing

CRISPR technology has revolutionized molecular biology by enabling precise DNA and RNA editing [327]. A CRISPR/Cas9-based approach has been developed to identify novel targets and pharmacological inhibitors of cancer innervation by endogenously tagging the neuronal marker β3-tubulin, thereby creating a fluorescent reporter system for high-throughput analysis of tumor-induced neural remodeling [328]. Beyond target discovery, CRISPR holds significant promise in correcting epigenomic alterations that drive tumorigenesis. By repairing mutations or deletions in key tumor suppressors such as tumor protein P53 *(TP53),* breast cancer gene *(BRCA1),* retinoblastoma gene *(RB1)*, and phosphatase and tensin homolog *(PTEN)'*, CRISPR/Cas9 restores tumor suppressive functions and impedes cancer progression [329, 330]. In the context of cancer-associated cachexia, antibody-mediated blockade of IL-6 reduces hyperactivity in the AP neural network and prolongs survival [329]. Similarly, CRISPR interference targeting IL-6 binding receptor, encoding the IL-6 receptor in AP neurons, achieves comparable therapeutic effects. Moreover, silencing Gfral-expressing AP neurons ameliorates malignant phenotypes and normalizes AP network hyperactivity, underscoring the potential of CRISPR-based neuromodulation in cancer therapy [329] (Fig. 5c).

Despite its transformative potential, the clinical translation of CRISPR technology faces significant challenges. Foremost among these is the development of efficient and specific delivery systems capable of transporting Cas proteins and guide RNAs to target cells and tissues while minimizing off-target effects. Current vectors, including adeno-associated viruses (AAVs), lentiviruses, and lipid NPs, exhibit limitations in delivery efficiency and safety, particularly for systemic administration and metastatic cancer targeting. Furthermore, a comprehensive understanding of the long-term biological consequences of CRISPR interventions is essential to assess their therapeutic viability and patient safety [331, 332].

#### AAV

The use of recombinant viral vectors has facilitated precise transgene delivery to specific neural cell populations throughout the nervous system [333]. Notably, AAV-mediated overexpression of triggering receptor expressed on myeloid cells 2 (TREM2) suppresses GBM growth and synergizes with immune checkpoint blockade [334]. Smith et al. [335] engineered a hybrid AAV and phage (AAVP) vector displaying biologically active octreotide (Oct) on its surface to enable ligand-directed delivery and internalization of a *TNF* transgene specifically to neuroendocrine tumors. This Oct-AAVP-TNF vector significantly reduced tumor metabolism and insulin secretion, diminished tumor size, and improved survival in murine models (Fig. 5d). Despite these advances, localized neural engineering using AAV vectors faces challenges. A recent study has demonstrated the efficacy of AAV vectors in treating breast cancer; however, their applicability to other types of tumors remains to be established [333]. Additionally, delivery efficiency in certain solid tumors, such as bone cancer, is limited, and clinical data supporting viral vector efficacy are sparse. Future efforts should focus on optimizing vector delivery systems to overcome these barriers.

In the emerging era of precision and individualized tumor therapy, approaches targeting tumor innervation have demonstrated considerable promise and clinical value. These strategies transcend the limitations of traditional monodimensional treatments by enabling systemic modulation of the TME through intervention in complex neuro-immune-tumor networks. Leveraging the multifunctionality of existing pharmacological agents facilitates rapid clinical translation, while cutting-edge technologies such as gene editing and nanodelivery platforms enable molecularly precise interventions. Nonetheless, the field faces significant hurdles, the so-called “last mile” challenges, including clinical translation barriers, potential off-target effects, and prohibitive treatment costs. Advancing these therapies will require sustained multidisciplinary collaboration focused on optimizing efficacy, safety, and accessibility to realize their full therapeutic potential.

### Theranostic technology

Recent advances in tumor innervation research have driven significant progress in tumor diagnosis and therapy, with emerging innovative technologies demonstrating great potential to integrate diagnostic and therapeutic modalities (Fig. 5e).

As our understanding of tumor-nervous system interactions deepens, neural features have emerged as promising biomarkers for disease prognosis and therapeutic response. Tumors induce structural and functional alterations in normal neuronal architecture, positioning the nervous system as a novel focus in biomarker research [336]. Currently, PNI is the sole neural-associated biomarker routinely integrated into clinical staging systems for cancers such as head and neck squamous cell carcinoma and penile cancer, where it is recorded as a binary variable (present or absent) [337]. Beyond PNI, the spatial proximity between nerves and tumor cells carries significant prognostic value. Patients lacking overt PNI but exhibiting shorter nerve-tumor distances experience worse outcomes, including reduced disease-specific and overall survival [338, 339]. Similarly, elevated nerve fiber density within the TME correlates with adverse prognosis, paralleling the established prognostic significance of immune infiltration [340, 341]. In PCa, increased nerve density associates with extracapsular extension, diminished recurrence-free survival, and heightened tumor proliferation [342, 343]. Oral cancers with higher standardized nerve density, adjusted for anatomical site, exhibit greater PNI incidence and poorer disease-specific survival [338], even in the absence of histological PNI, underscoring the independent prognostic relevance of nerve density. Moreover, distinct neural phenotypes may serve as predictive markers. For instance, heightened sympathetic nerve density, identified by tyrosine hydroxylase expression, correlates with biochemical recurrence in PCa and inferior survival in head and neck cancers [344]. Regenerating neurons expressing growth-associated protein 43 also portend poorer prognosis in PCa, suggesting that nerve repair responses to tumor-induced injury hold prognostic implications [345].

Bioinformatics and experimental approaches are increasingly integral to identifying precise cancer targets. Advances in single-cell and bulk RNA sequencing technologies enable comprehensive characterization of cellular heterogeneity and gene expression within tumors [346]. For example, bioinformatic analyses have linked glia maturation factor beta expression with prognosis in kidney renal clear cell carcinoma, where glia maturation factor beta modulates macrophage and resting mast cell infiltration, establishing it as an independent prognostic biomarker and therapeutic candidate [347]. Similarly, Zhang et al. [348] identified a 4-gene neurotransmitter receptor signature, CHRNA3, GABRD, GRIK3, and GRIK5, that predicts prognosis and treatment response in CRC patients. Liquid biopsy of cerebrospinal fluid has emerged as a minimally invasive method to detect circulating biomarkers, predominantly in CNS tumors [349]. MicroRNAs such as miR-15a, miR-16, and miR-21 have been proposed as diagnostic and prognostic biomarkers for gliomas [350]. Nonetheless, rigorous clinical validation remains essential to establish their clinical utility.

Wen Liang Lu et al. [351] have developed an advanced near-infrared fluorescence probe exhibiting high specificity for targeted delivery. This probe selectively binds growth-associated protein 43-positive neuronal cells in an in vitro model of PNI and enables effective visualization of PNI lesions in preclinical pancreatic cancer models. Peptide-receptor radionuclide therapy (PRRT) represents another promising therapeutic modality[352]. Radiolabeled neuropeptides, such as somatostatin and vasoactive intestinal peptide analogs, facilitate tumor imaging and targeted radiotherapy by exploiting the overexpression of cognate receptors on tumor cells, which internalize and accumulate the radiopharmaceuticals [353, 354]. While somatostatin receptor-based radiotherapy has been extensively studied [355], clinical application of vasoactive intestinal peptide receptor-targeted radiotherapy remains limited due to insufficient receptor specificity [355, 356].

In PCa diagnosis and treatment, propranolol-conjugated superparamagnetic iron oxide nanoparticles (PSN NPs) integrated with dual-modality imaging, magnetic resonance imaging, and magnetic particle imaging, enable accurate assessment of tumor nerve density and aggressiveness, while facilitating targeted neuropharmacological delivery [357]. The T-SMPDC system, engineered for neuroendocrine PCa, combines positron emission tomography imaging and chemotherapy by harnessing ^68^Ga labeling and octreopeptide receptor targeting for precise tumor localization, alongside delivery of the sphingosine kinase 1 inhibitor FTY720 to circumvent drug resistance [358]. Utilizing enzyme-responsive linkers to control intracellular drug release, this platform exemplifies a novel paradigm in precision therapy for neuroendocrine PCa [359].

These advances across diverse tumor types have significantly propelled the integration of diagnostic and therapeutic strategies targeting tumor innervation, offering novel insights and technological platforms for precision oncology. Such progress is poised to drive further improvements in the accurate diagnosis and effective treatment of cancers.

### Combination therapy

Recent years have witnessed a paradigm shift in cancer therapy towards multidimensional and combination approaches, exemplified by the integrated use of chemotherapy, immunotherapy, and targeted therapies (Fig. 5f). Combining chemotherapy with immunotherapy can reduce treatment-related neurotoxicity. In breast cancer models, epidermal growth factor receptor activation in sensory neurons induces Dickkopf-related protein 1, leading to peripheral neuropathy. Combined administration of an anti-Dickkopf-related protein 1 antibody and paclitaxel not only enhanced antitumor efficacy but also alleviated neurotoxic effects, suggesting a promising low-toxicity chemo-immunotherapy strategy for breast cancer [359].

The combination of radiation therapy with neuroactive agents has shown promise in augmenting antitumor efficacy. Taylor P. Uccello et al. [360] demonstrated that short-course radiation therapy induces the expression of neurogenesis-related genes, elevates local NE secretion, and upregulates β-AR expression in a murine model. These adverse effects were effectively mitigated by β-AR antagonism using propranolol. Furthermore, administration of the AMPAR antagonist PER significantly attenuated radiation-induced synaptic activity within tumor-innervating neurons and disrupted tumor-neuron network connectivity [361]. This combinatorial approach reduced neuronal action potential frequency and suppressed synaptic electrical activity in the TME. Importantly, the combined treatment resulted in a nearly 40% greater reduction in tumor volume and substantially prolonged survival in treated mice [361].

The synergistic potential of combining denervation with chemotherapeutic agents warrants particular attention. In mouse models of melanoma and colon cancer, co-administration of PD-1 immune checkpoint inhibitors with botulinum toxin type A1 significantly enhanced anti-tumor responses [362]. Additionally, prolonged tamoxifen treatment has been shown to upregulate prolactin receptor (PRLR) transcription and increase PRLR protein accumulation in breast cancer cells by alkalinizing lysosomes [363]. Activation of the prolactin-PRLR axis substantially diminishes tamoxifen sensitivity in estrogen receptor α-positive breast cancer cells. Notably, the PRLR-targeted immunotoxin N8-PE24 improved tamoxifen efficacy by enhancing intracellular drug retention via intronic peptide constructs, resulting in markedly increased chemotherapeutic potency in PRLR-positive TNBC and xenograft models [363]. In summary, combination therapies hold great promise for enhancing the efficacy of current treatment modalities, reducing adverse effects, and enabling more precise, personalized interventions. By deeply analyzing and targeting the TME, cancer treatment is evolving beyond single-agent approaches toward multifaceted regimens that synergistically suppress tumor growth, metastasis, and recurrence.

### Optogenetic tools

Optogenetics represents a highly precise approach for targeted neuromodulation. Fang et al. [364] employed optogenetic tools to elucidate neuro-immune interactions within the skeletal system, revealing a bidirectional link between nociceptors and immune cells. In freely moving mice, light activation of channelrhodopsin-2-expressing nociceptors elicited nociceptive behaviors indicative of ascending pain signaling, which subsequently potentiated bone immune responses in the absence of inflammation and amplified inflammatory reactions following infection, engaging peripheral nociceptor terminals in feedback regulation. Another study demonstrated that optogenetic stimulation of dopaminergic neurons in the ventral tegmental area or their projections to the nucleus accumbens transiently alleviated neuropathic pain in mouse models without altering thermal sensitivity thresholds in control animals [365]. Moreover, ventral tegmental area dopaminergic terminal activation within the medial prefrontal cortex mitigated chronic stress-induced anxiety-like behaviors and significantly suppressed breast tumor progression [366]. Integration of bioelectronics with optogenetics offers a minimally invasive or non-invasive platform to interrogate brain-organ neural circuits, enabling real-time physiological recording and modulation while circumventing the need for traditional invasive blue light implants [367] (Fig. 5g).

### RNA-based therapeutics

The intricate interplay between cancer cells and the nervous system presents a formidable challenge for therapeutic intervention. RNA-based therapeutic drugs have many advantages, including target accessibility, permeability of their organization, stable quality control, and low production costs, making them particularly promising for modulating tumor-associated neural activity[368]. The clinical approval of mRNA-based drugs and vaccines underscores the translational potential of RNA therapeutics [369–371]. Hematopoietic stem cells (HSCs), which contribute to hematologic malignancies, are innervated by adrenergic nerves [372]. Recent advances [373, 374] demonstrate that lipid NP systems targeting p53-responsive receptors on HSCs enable selective depletion, facilitating durable genome editing and bone marrow transplantation without genotoxicity. Circular RNA vaccines have also been shown to elicit cytotoxic T cell-mediated tumor clearance in melanoma models [375]. These approaches leverage mRNA delivery to regulate neuronal activity, a process intimately linked to tumor growth and metastasis, thus offering a novel axis for cancer control.

However, research into RNA-encoded neuromodulators for cancer therapy remains nascent, warranting further exploration. RNA-based therapeutics, encompassing antisense oligonucleotides (ASOs), RNA interference, CRISPR/Cas systems, and RNA aptamers, modulate gene expression, silencing, and protein synthesis by targeting specific RNA sequences, representing a versatile toolkit for cancer intervention [376–378] (Fig. 5h). Zhang et al. [379] demonstrated that inhaled let-7b microRNA significantly inhibits tumor growth in both benzo[a]pyrene (B[a]P)-induced and syngeneic lung cancer models, highlighting its potential for lung cancer prevention. Similarly, treatment with miR-10b ASOs suppresses target gene expression and impedes GBM progression [380]. siRNAs nanotherapeutics have garnered considerable attention for their ability to cross the blood-brain barrier, achieve sustained plasma circulation, and enable responsive intracellular release, resulting in potent inhibition of GBM growth [381–383].

However, scalable and cost-effective manufacturing remains a critical challenge. Establishing a comprehensive production pipeline for mRNA-based therapeutics, with optimized in vitro transcription processes to maximize RNA yield and minimize by-products, is imperative to meet preclinical and clinical demand. Technological advancements continue to position mRNA as a promising modality in cancer therapy, and leveraging RNA-based regulation of neural signaling holds significant potential to transform future oncological interventions.

## Future directions

A critical hurdle in translating neural-tumor interaction research into clinical practice is identifying patients most likely to benefit from neuro-targeted therapies. Considerable heterogeneity exists in neural infiltration levels within the TME, nerve subtype composition (e.g., sympathetic, parasympathetic, and sensory), and the activity of neurotrophic signaling pathways such as NGF, BDNF, and GDNF. Additionally, neural regulators variably influence immune infiltration, nociception, and metastatic propensity across individuals. Current stratification approaches, predominantly based on tumor genetics or broad histopathological criteria, fail to capture the functional complexity and dynamic nature of the neural niche. There is an urgent need for integrative stratification frameworks combining neurogenomic profiling, tumor molecular subtyping, clinical phenotyping, multi-omics data, and neural-associated biomarkers, including advanced imaging modalities. Such models would enable precise identification of patient subsets most amenable to neuro-targeted interventions [384]. Ongoing clinical trials leveraging patient-derived organoids to delineate individualized neural molecular signatures hold significant promise for advancing personalized neuro-oncological therapies [5].

In recent years, organoids have emerged as physiologically relevant three-dimensional in vitro models that preserve tumor heterogeneity and microenvironmental complexity, faithfully recapitulating genetic lineages, spatial architecture, and dynamic processes such as neural innervation [385]. Lu et al. [386] generated human DRG organoids by precise activation of endogenous signaling pathways, revealing functional neurotrophic tropomyosin receptor kinase 3 positive (NTRK3⁺)/DCC⁺ (DCC netrin-1 receptor) nociceptors. In CRC, loss of N‐Myc downstream‐regulated gene 4 (Ndrg4) function in organoid models triggered neuronal secretion of tumor-promoting factors, including nidogen 1 and fibulin 2, identifying novel therapeutic targets within neurogenic signaling pathways [387]. A major advantage of organoids in drug discovery lies in their amenability to high-throughput screening and personalized therapeutic testing. By co-culturing patient-derived GBM tumor-like cells with human-induced pluripotent stem cell-derived cerebral organoids, researchers established a GBM tumor-brain organoid assembly that preserves intratumoral heterogeneity while partially recapitulating normal brain tissue architecture [388]. Despite their promise, organoid platforms face challenges including limited model complexity, standardization and scalability constraints, and a paucity of clinical validation [389]. Integration of artificial intelligence (AI)-driven analytics, spatial transcriptomics, and microfluidic technologies promises to deepen insights into tumor neurobiology and accelerate the translation of organoid-based models from bench to bedside [390].

Cancer therapies have the potential to disrupt the neural homeostasis and plasticity mechanisms that tumors exploit for growth. However, these interventions frequently induce off-target effects on normal neural functions, manifesting as a debilitating cognitive impairment syndrome characterized by deficits in attention, memory, processing speed, multitasking, and executive function [391]. Additionally, they may provoke neuropathies affecting sensory, motor, and autonomic peripheral nerves [392], including peripheral sensory abnormalities and focal mononeuropathies associated with anti-NGF antibodies, alongside joint-related adverse events [393]. BDNF and its receptor TrkB are critical regulators of mood, with inhibition of BDNF signaling linked to anxiety and depression [393]. Given that many neural-tumor interaction pathways targeted therapeutically also underpin normal nervous system functions, it is imperative to elucidate the mechanisms and consequences of treatment-induced neurotoxicity. This entails defining therapeutic windows that optimize antitumor efficacy while minimizing neural damage, and restricting interventions such as denervation to extracranial tumor sites when appropriate. Rigorous monitoring of CNS and PNS adverse effects is essential throughout treatment [5]. Ongoing research into the molecular and cellular bases of therapy-related neurotoxicity is uncovering novel neuroprotective and neuroregenerative strategies, offering hope for mitigating these debilitating side effects [394, 395].

The systemic nature of cancer necessitates a therapeutic paradigm that extends beyond localized tumor control to address the complex TME and the dynamic interactions among multiple organ systems. Comorbidities and physiological cascades intricately influence disease progression and therapeutic resilience. Cancer patients often present with a spectrum of comorbid conditions, notably cardiovascular diseases, which may be aggravated by the physiological stress of cancer and its treatment [396]. Psychological distress associated with diagnosis and therapy further contributes to psychiatric disorders such as depression and anxiety [397]. Organ-specific comorbidities also complicate management; for example, chronic obstructive pulmonary disease frequently co-occurs with lung cancer, while breast cancer patients may develop treatment-related lymphedema [396]. These comorbidities add layers of complexity to cancer care, as exemplified by patients with pre-existing cardiac dysfunction who may be unable to tolerate intensive chemotherapy regimens [396]. To overcome these challenges, several strategies are under active exploration. First, personalized medicine leveraging advanced genomic and proteomic technologies offers insights into molecular characteristics of both tumors and comorbidities, guiding tailored treatment approaches [398]. Second, multidisciplinary collaboration is imperative, requiring oncologists to coordinate care with specialists in cardiology, endocrinology, psychiatry, and other relevant fields to deliver comprehensive, patient-centered management [399]. Effective integration of comorbidity management into oncology care promises to enhance survival outcomes and foster a more humane, precise approach to cancer treatment.

Cancer treatment increasingly depends not only on pharmaceutical agents but also on interdisciplinary innovations and technological integration, which offer novel solutions at molecular and systemic levels. Advances in optogenetics and chemical genetics have made precise modulation of specific neural pathways and tumor cells increasingly feasible. Chemical genetics enables direct control over tumor growth, metastasis, and immune responses by engineering drugs to selectively activate defined receptors or neural circuits [400–402]. Specifically, genetically engineered cells can express designer receptors exclusively activated by designer drugs (DREADDs), allowing selective modulation of neuronal activity: Gq-coupled DREADDs enhance neuronal firing, whereas Gi/o-coupled DREADDs suppress it [403–405]. Compared to ES, magnetic stimulation offers superior tissue penetration and a non-invasive profile, while magnetic NP-mediated electromagnetic stimulation of the VN addresses the spatial precision limitations of conventional magnetic techniques [111]. External electromagnetic field interventions can mimic the effects of pharmacological or other stimulations on nerve activity, potentially avoiding the adverse effects and addiction risks associated with drug therapies [406]. Patch-seq technology, which integrates high-resolution imaging with computational analysis, enables precise screening of compounds capable of crossing the blood-brain barrier and targeting cancer cells [206]. Similarly, high-throughput drug screening platforms, such as pharmacoscopy, allow rapid evaluation of hundreds of candidate drugs in living cells, expediting tumor therapeutic discovery [275]. These advanced screening methodologies are poised to accelerate the identification and clinical translation of innovative neuro-targeted cancer therapies.

The selection and optimization of drug delivery systems are paramount for achieving precise targeting of tumor-associated neural structures, which directly impacts therapeutic efficacy, toxicity profiles, and clinical feasibility. Cell reprogramming technologies have advanced considerably, with successful applications in disease modeling, cell transplantation, autologous therapies, and drug and toxicity screening. Nonetheless, despite improvements in viral and non-viral delivery platforms driven by enhanced reprogramming efficiencies, the clinical translation of cell reprogramming remains constrained by technical challenges [407]. Rationally designed nanomaterials offer promising avenues to overcome these limitations. Engineered nanocarriers can be programmed for targeted delivery, controlled release, and site-specific therapeutic action. By integrating organic and inorganic components, these systems capitalize on complementary advantages, such as the positive surface charge of organic NPs to promote cellular uptake, alongside the biocompatibility and structural stability of inorganic materials, while mitigating issues like immunogenicity, cytotoxicity, and limited biodegradability [407]. Similarly, synthetic nanocarriers such as liposomes, benefit from well-established manufacturing processes and long-standing clinical use, and EVs have emerged as a promising alternative for site-specific delivery owing to their endogenous origin and inherent biocompatibility. Optimal selection of EV subtypes and meticulous preservation of their intrinsic properties, achieved by minimizing damage during isolation, drug loading, and labeling, alongside controlled infusion rates, can reduce rapid immune clearance [408–410]. However, substantial challenges remain for clinical translation, including the heterogeneity of EVs isolation techniques and inconsistent enrichment efficiencies, which hinder standardization and scalability [411].

## Conclusions

The dynamic interplay between the nervous system and cancer has catalyzed a paradigm shift in oncology, positioning cancer neuroscience at the forefront of transformative therapeutic innovations. We present a comprehensive integration of the multidimensional mechanisms underpinning tumor innervation across tumorigenesis, immune evasion, metastasis, and therapeutic resistance, and propose a conceptual framework for targeting neural-tumor interactions to disrupt oncogenic networks. Cutting-edge interdisciplinary technologies, including nanomedicine delivery platforms, CRISPR/Cas9-mediated genome editing, and RNA-based therapeutics have revolutionized strategies for modulating the TME. Among these, engineered cell therapies such as CAR-T cells bearing neuro-targeted scFvs exemplify the convergence of synthetic biology and oncology to enhance treatment specificity [412]. Concurrently, drug repurposing approaches disrupt tumor-neurosymbiosis by redeploying established neuropharmacological agents, thereby expediting clinical translation.

Nonetheless, a fundamental challenge persists: whether neuro-targeted strategies can achieve sufficient specificity, robustness, and consistent therapeutic benefit across diverse tumor types. Current evidence reveals pronounced heterogeneity in tumor innervation among malignancies, with cancers such as gliomas, PC, and PCa exhibiting greater dependence on neural crosstalk [413]. Furthermore, variations in dominant biological features across tumor types critically influence therapeutic priorities. In addition, the effects of neural ablation are highly dependent on both nerve subtype and tumor context, reinforcing the need to develop tailored strategies that target specific neural components within the TME. Consequently, these neuro-targeted approaches are not universally applicable, and no single strategy can be regarded as a pan-cancer solution.

How can we achieve tumor-neural axis targeting with sufficient specificity to deliver broad clinical benefit? Addressing this challenge requires a deeper understanding of the underlying pathological mechanisms. Integrative multi-omics approaches, combining genomics, proteomics, transcriptomics, and bioinformatics, enable us to transcend traditional mechanistic views and elucidate the spatiotemporal dynamics of tumor-neural communication. Given that many neural-tumor interactions engage signaling pathways also active in normal tissue development and function, alongside the paradoxical effects of neuro-immune mediators and the intricate crosstalk of systemic neuroendocrine regulation, these multifaceted interactions are inherently complex. Consequently, defining precise therapeutic windows for tumor-specific interventions is critical. Early-phase clinical trials should prioritize identification of suitable patient cohorts by considering disease stage, combination regimens, and quantifiable therapeutic targets. The integration of serum, cerebrospinal fluid, and tumor tissue biomarkers, together with advanced imaging modalities, may facilitate the stratification of patients most likely to respond to neuro-targeted therapies. Adaptive trial designs will be essential to optimize dosing and timing strategies, thereby maximizing therapeutic efficacy. The advent of AI-driven drug screening, intelligent diagnostic and therapeutic platforms, and advances in bioelectronics and neuromodulation technologies, particularly the precision afforded by engineered cellular delivery systems such as exosome-coated RNA interference and optogenetically controlled drug release, offers unprecedented opportunities to overcome current limitations in targeted innervation therapies. By minimizing off-target effects while enhancing therapeutic specificity, these strategies promise to shift cancer treatment paradigms from aggressive cytotoxicity toward systemic regulation. This evolution holds the potential to extend patient survival and improve quality of life by fostering more durable, finely tuned interventions.

## Data Availability

Not applicable.

## Abbreviations

β-AR: β-Adrenergic receptor
AAV: Adeno-associated virus
ADAM10: A disintegrin and metalloproteinase domain-containing protein 10
ACh: Acetylcholine
AEDs: Antiepileptic drugs
AMPAR: α-Amino-3-hydroxy-5-methyl-4-isoxazolepropionic acid receptor
AP: Area postrema
AR: Adrenergic receptors
BDNF: Brain-derived neurotrophic factor
B2BM: Breast-to-brain metastases
CAR-T: Chimeric antigen receptor T cell
Cas: CRISPR-associated protein
CCL: C-C motif chemokine ligand
CeMC: Central medial amygdala cortex
CeM^CRH^: CRH neurons in the central medial amygdala
CGRP: Calcitonin gene-related peptide
CIPN: Chemotherapy-induced peripheral neuropathy
CNS: Central nervous system
CRC: Colorectal cancer
CRISPR: Clusters of regularly interspaced short palindromic repeats
CRH: Corticotropin-releasing hormone
CSCs: Cancer stem cells
DRG: Dorsal root ganglion
DREADDs: Designer receptors exclusively activated by designer drugs
EA: Electroacupuncture
ER: Endoplasmic reticulum
ERK: Extracellular signal-regulated kinase
ES: Electrical stimulation
EVs: Extracellular vesicles
GABA: γ-aminobutyric acid
GBM: Glioblastoma
GR: Glucocorticoid receptors
HPA: Hypothalamic-pituitary-adrenal
IDH: Isocitrate dehydrogenase
IL: Interleukin
KP: Kisspeptin
LEV: Levetiracetam
LPGi^CA^: Catecholaminergic neurons in the lateral paragigantocellular nucleus
M3R: M3 muscarinic acetylcholine receptor
MDSCs: Myeloid-derived suppressor cells
MEK: Mitogen-activated protein kinase kinase
METTL3: Methyltransferase-like 3
MMP: Matrix metalloproteinases
NE: Norepinephrine
NGF: Nerve growth factor
NLGN3: Neuroligin-3
NMDAR: N-methyl-D-aspartate receptor
NPs: Nanoparticles
NPY: Neuropeptide Y
NPY1R: Neuropeptide Y receptor type 1
NrCAM: Neuronal cell adhesion molecule
NSCLC: Non-small cell lung cancer
NTF4: Neurotrophin 4
OLZ: Olanzapine
OPCs: Oligodendrocyte precursor cells
PC: Pancreatic cancer
PCa: Prostate cancer
PDAC: Pancreatic ductal adenocarcinoma
PD-1: Programmed death-1
PD-L1: Programmed cell death-ligand 1
PER: Perampanel
PI3K: Phosphoinositol 3-kinase
PNS: Peripheral nervous system
proBDNF: Precursor for brain-derived neurotrophic factor
PNI: Perineural invasion
PRLR: Prolactin receptor
ROBO1: Roundabout guidance receptor 1
SCs: Schwann cells
Sema4D: Semaphorin 4D
scFvs: Single-chain variable fragments
siRNAs: Small interfering RNAs
SP: Substance P
TACR1: Tumor acinar peptide receptor 1
TAMs: Tumor-associated macrophages
TFF2: Trefoil factor 2
TME: Tumor microenvironment
TMs: Tumor microtubes
TNBC: Triple-negative breast cancer
TNF: Tumor necrosis factor
Trk: Tropomyosin receptor kinase
TRPA1: Transient receptor potential ankyrin 1
TRPV1: Transient receptor potential vanilloid 1
VEGF: Vascular endothelial growth factor
VN: Vagus nerve
5-HT: 5-hydroxytryptamine

## References

[1] Sung H, Ferlay J, Siegel RL, Laversanne M, Soerjomataram I, Jemal A, et al. Global cancer statistics 2020: globocan estimates of incidence and mortality worldwide for 36 cancers in 185 countries. CA Cancer J Clin. 2021;71(3):209–49. 33538338 10.3322/caac.21660

[2] Boilly B, Faulkner S, Jobling P, Hondermarck H. Nerve dependence: from regeneration to cancer. Cancer Cell. 2017;31(3):342–54. 28292437 10.1016/j.ccell.2017.02.005

[3] Johnston APW, Miller FD. The contribution of innervation to tissue repair and regeneration. Cold Spring Harb Perspect Biol. 2022;14(9):a041233. 35667791 10.1101/cshperspect.a041233 PMC9438784

[4] Magnon C, Hondermarck H. The neural addiction of cancer. Nat Rev Cancer. 2023;23(5):317–34. 37041409 10.1038/s41568-023-00556-8

[5] Winkler F, Venkatesh HS, Amit M, Batchelor T, Demir IE, Deneen B, et al. Cancer neuroscience: state of the field, emerging directions. Cell. 2023;186(8):1689–707. 37059069 10.1016/j.cell.2023.02.002 PMC10107403

[6] Monje M, Borniger JC, D’Silva NJ, Deneen B, Dirks PB, Fattahi F, et al. Roadmap for the emerging field of cancer neuroscience. Cell. 2020;181(2):219–22. 32302564 10.1016/j.cell.2020.03.034 PMC7286095

[7] Griffin N, Faulkner S, Jobling P, Hondermarck H. Targeting neurotrophin signaling in cancer: the renaissance. Pharmacol Res. 2018;135:12–7. 30031169 10.1016/j.phrs.2018.07.019

[8] Bahmad HF, Wegner C, Nuraj J, Avellan R, Gonzalez J, Mendez T, et al. Perineural invasion in breast cancer: a comprehensive review. Cancers (Basel). 2025;17(12):1900. 40563551 10.3390/cancers17121900 PMC12190579

[9] Gysler SM, Drapkin R. Tumor innervation: peripheral nerves take control of the tumor microenvironment. J Clin Invest. 2021;131(11):e147276. 34060481 10.1172/JCI147276 PMC8159682

[10] Zahalka AH, Frenette PS. Nerves in cancer. Nat Rev Cancer. 2020;20(3):143–57. 31974491 10.1038/s41568-019-0237-2 PMC7709871

[11] Chen H, Liu D, Guo L, Cheng X, Guo N, Shi M. Chronic psychological stress promotes lung metastatic colonization of circulating breast cancer cells by decorating a pre-metastatic niche through activating β-adrenergic signaling. J Pathol. 2018;244(1):49–60. 28940209 10.1002/path.4988

[12] Ceyhan GO, Bergmann F, Kadihasanoglu M, Erkan M, Park W, Hinz U, et al. The neurotrophic factor artemin influences the extent of neural damage and growth in chronic pancreatitis. Gut. 2007;56(4):534–44. 17047099 10.1136/gut.2006.105528 PMC1856869

[13] Flores ER, Sawyer WG. Engineering cancer’s end: an interdisciplinary approach to confront the complexities of cancer. Cancer Cell. 2024;42(7):1133–7. 38848721 10.1016/j.ccell.2024.05.017

[14] Hernandez S, Serrano AG, Solis Soto LM. The role of nerve fibers in the tumor immune microenvironment of solid tumors. Adv Biol. 2022;6(9):e2200046. 10.1002/adbi.202200046 35751462

[15] Knox SM, Lombaert IM, Reed X, Vitale-Cross L, Gutkind JS, Hoffman MP. Parasympathetic innervation maintains epithelial progenitor cells during salivary organogenesis. Science. 2010;329(5999):1645–7. 20929848 10.1126/science.1192046 PMC3376907

[16] Knox SM, Lombaert IM, Haddox CL, Abrams SR, Cotrim A, Wilson AJ, et al. Parasympathetic stimulation improves epithelial organ regeneration. Nat Commun. 2013;4:1494. 23422662 10.1038/ncomms2493 PMC3582394

[17] Carmeliet P. Angiogenesis in health and disease. Nat Med. 2003;9(6):653–60. 12778163 10.1038/nm0603-653

[18] Saloman JL, Albers KM, Li D, Hartman DJ, Crawford HC, Muha EA, et al. Ablation of sensory neurons in a genetic model of pancreatic ductal adenocarcinoma slows initiation and progression of cancer. Proc Natl Acad Sci U S A. 2016;113(11):3078–83. 26929329 10.1073/pnas.1512603113 PMC4801275

[19] Silva D, Quintas C, Gonçalves J, Fresco P. Contribution of adrenergic mechanisms for the stress-induced breast cancer carcinogenesis. J Cell Physiol. 2022;237(4):2107–27. 35243626 10.1002/jcp.30707

[20] Albo D, Akay CL, Marshall CL, Wilks JA, Verstovsek G, Liu H, et al. Neurogenesis in colorectal cancer is a marker of aggressive tumor behavior and poor outcomes. Cancer. 2011;117(21):4834–45. 21480205 10.1002/cncr.26117

[21] Allen JK, Armaiz-Pena GN, Nagaraja AS, Sadaoui NC, Ortiz T, Dood R, et al. Sustained adrenergic signaling promotes intratumoral innervation through bdnf induction. Cancer Res. 2018;78(12):3233–42. 29661830 10.1158/0008-5472.CAN-16-1701 PMC6004256

[22] Partecke LI, Speerforck S, Käding A, Seubert F, Kühn S, Lorenz E, et al. Chronic stress increases experimental pancreatic cancer growth, reduces survival and can be antagonised by beta-adrenergic receptor blockade. Pancreatology. 2016;16(3):423–33. 27083074 10.1016/j.pan.2016.03.005

[23] Schonkeren SL, Thijssen MS, Vaes N, Boesmans W, Melotte V. The emerging role of nerves and glia in colorectal cancer. Cancers (Basel). 2021;13(1):152. 33466373 10.3390/cancers13010152 PMC7796331

[24] Le TT, Oudin MJ. Understanding and modeling nerve-cancer interactions. Dis Model Mech. 2023;16(1):dmm049729. 36621886 10.1242/dmm.049729 PMC9844229

[25] Jurcak NR, Rucki AA, Muth S, Thompson E, Sharma R, Ding D, et al. Axon guidance molecules promote perineural invasion and metastasis of orthotopic pancreatic tumors in mice. Gastroenterology. 2019;157(3):838–50.e6. 31163177 10.1053/j.gastro.2019.05.065 PMC6707836

[26] Jiang CC, Marsland M, Wang Y, Dowdell A, Eden E, Gao F, et al. Tumor innervation is triggered by endoplasmic reticulum stress. Oncogene. 2022;41(4):586–99. 34785777 10.1038/s41388-021-02108-6

[27] Banh RS, Biancur DE, Yamamoto K, Sohn ASW, Walters B, Kuljanin M, et al. Neurons release serine to support mrna translation in pancreatic cancer. Cell. 2020;183(5):1202–18.e25. 33142117 10.1016/j.cell.2020.10.016 PMC8100789

[28] Jiang S-H, Zhang S, Cai Z, Yu M-H, Li H, Jiang L, et al. Mechanical cues of extracellular matrix determine tumor innervation. bioRxiv. 2024:2024.03.25.586535.

[29] Madeo M, Colbert PL, Vermeer DW, Lucido CT, Cain JT, Vichaya EG, et al. Cancer exosomes induce tumor innervation. Nat Commun. 2018;9(1):4284. 30327461 10.1038/s41467-018-06640-0 PMC6191452

[30] Vermeer PD. Exosomal induction of tumor innervation. Cancer Res. 2019;79(14):3529–35. 31088834 10.1158/0008-5472.CAN-18-3995 PMC6635078

[31] Hoffman MT, Kemp SB, Salas-Escabillas DJ, Zhang Y, Steele NG, The S, et al. The gustatory sensory g-protein gnat3 suppresses pancreatic cancer progression in mice. Cell Mol Gastroenterol Hepatol. 2021;11(2):349–69. 32882403 10.1016/j.jcmgh.2020.08.011 PMC7779788

[32] Ge Y, Liu H, Zhang Y, Liu J, Yan R, Xiao Z, et al. Inhibition of DCLK1 kinase reverses epithelial-mesenchymal transition and restores T-cell activity in pancreatic ductal adenocarcinoma. Transl Oncol. 2022;17:101317. 34998236 10.1016/j.tranon.2021.101317 PMC8739467

[33] Ma Z, Lytle NK, Chen B, Jyotsana N, Novak SW, Cho CJ, et al. Single-cell transcriptomics reveals a conserved metaplasia program in pancreatic injury. Gastroenterology. 2022;162(2):604–20.e20. 34695382 10.1053/j.gastro.2021.10.027 PMC8792222

[34] DelGiorno KE, Chung CY, Vavinskaya V, Maurer HC, Novak SW, Lytle NK, et al. Tuft cells inhibit pancreatic tumorigenesis in mice by producing prostaglandin d_2_. Gastroenterology. 2020;159(5):1866–81.e8. 32717220 10.1053/j.gastro.2020.07.037 PMC7680354

[35] Salas-Escabillas DJ, Hoffman MT, Brender SM, Moore JS, Wen HJ, Benitz S, et al. Tuft cells transdifferentiate to neural-like progenitor cells in the progression of pancreatic cancer. Dev Cell. 2025;60(6):837–52.e3. 39721583 10.1016/j.devcel.2024.12.003 PMC12225690

[36] Amit M, Takahashi H, Dragomir MP, Lindemann A, Gleber-Netto FO, Pickering CR, et al. Loss of p53 drives neuron reprogramming in head and neck cancer. Nature. 2020;578(7795):449–54. 32051587 10.1038/s41586-020-1996-3 PMC9723538

[37] Ayanlaja AA, Ji G, Wang J, Gao Y, Cheng B, Kanwore K, et al. Doublecortin undergo nucleocytoplasmic transport via the rangtpase signaling to promote glioma progression. Cell Commun Signal. 2020;18(1):24. 32050972 10.1186/s12964-019-0485-5 PMC7017634

[38] Szymańska-Chabowska A, Świątkowski F, Jankowska-Polańska B, Mazur G, Chabowski M. Nestin expression as a diagnostic and prognostic marker in colorectal cancer and other tumors. Clin Med Insights Oncol. 2021;15:11795549211038256. 34421318 10.1177/11795549211038256 PMC8377314

[39] Mauffrey P, Tchitchek N, Barroca V, Bemelmans AP, Firlej V, Allory Y, et al. Progenitors from the central nervous system drive neurogenesis in cancer. Nature. 2019;569(7758):672–8. 31092925 10.1038/s41586-019-1219-y

[40] Silverman DA, Martinez VK, Dougherty PM, Myers JN, Calin GA, Amit M. Cancer-associated neurogenesis and nerve-cancer cross-talk. Cancer Res. 2021;81(6):1431–40. 33334813 10.1158/0008-5472.CAN-20-2793 PMC7969424

[41] Lu R, Fan C, Shangguan W, Liu Y, Li Y, Shang Y, et al. Neurons generated from carcinoma stem cells support cancer progression. Signal Transduct Target Ther. 2017;2:16036. 29263908 10.1038/sigtrans.2016.36 PMC5657421

[42] Wang J, Chen Y, Li X, Zou X. Perineural invasion and associated pain transmission in pancreatic cancer. Cancers (Basel). 2021;13(18):4594. 34572820 10.3390/cancers13184594 PMC8467801

[43] Cai Z, Yao H, Chen J, Ahmed AA, Li C, Hu X, et al. Schwann cells in pancreatic cancer: unraveling their multifaceted roles in tumorigenesis and neural interactions. Cancer Lett. 2024;587:216689. 38367898 10.1016/j.canlet.2024.216689

[44] Ni B, Yin Y, Li Z, Wang J, Wang X, Wang K. Crosstalk between peripheral innervation and pancreatic ductal adenocarcinoma. Neurosci Bull. 2023;39(11):1717–31. 37347365 10.1007/s12264-023-01082-1 PMC10603023

[45] Li J, Kang R, Tang D. Cellular and molecular mechanisms of perineural invasion of pancreatic ductal adenocarcinoma. Cancer Commun. 2021;41(8):642–60. 10.1002/cac2.12188 PMC8360640 34264020

[46] Ayala G. Neuroepithelial interactions in cancer. Annu Rev Pathol. 2023;18:493–514. 36323005 10.1146/annurev-pathmechdis-031521-023248

[47] Zorick TS, Lemke G. Schwann cell differentiation. Curr Opin Cell Biol. 1996;8(6):870–6. 8939676 10.1016/s0955-0674(96)80090-1

[48] Deborde S, Wong RJ. How schwann cells facilitate cancer progression in nerves. Cell Mol Life Sci. 2017;74(24):4405–20. 28631007 10.1007/s00018-017-2578-x PMC5665723

[49] Bosch-Queralt M, Fledrich R, Stassart RM. Schwann cell functions in peripheral nerve development and repair. Neurobiol Dis. 2023;176:105952. 36493976 10.1016/j.nbd.2022.105952

[50] Jessen KR, Mirsky R, Lloyd AC. Schwann cells: development and role in nerve repair. Cold Spring Harb Perspect Biol. 2015;7(7):a020487. 25957303 10.1101/cshperspect.a020487 PMC4484967

[51] Chen Z, Fang Y, Jiang W. Important cells and factors from tumor microenvironment participated in perineural invasion. Cancers (Basel). 2023;15(5):1360. 36900158 10.3390/cancers15051360 PMC10000249

[52] Rangel-Sosa MM, Mann F, Chauvet S. Pancreatic schwann cell reprogramming supports cancer-associated neuronal remodeling. Glia. 2024;72(10):1840–61. 38961612 10.1002/glia.24586

[53] Dong Z, Wang Y, Jin W. The neuroscience of cancer: focus on neuropeptidergic systems. Acta Pharm Sin B. 2025;15(5):2323–50. 40487638 10.1016/j.apsb.2025.03.025 PMC12144987

[54] Moody TW, Chan D, Fahrenkrug J, Jensen RT. Neuropeptides as autocrine growth factors in cancer cells. Curr Pharm Des. 2003;9(6):495–509. 12570813 10.2174/1381612033391621

[55] Deganutti G, Atanasio S, Rujan RM, Sexton PM, Wootten D, Reynolds CA. Exploring ligand binding to calcitonin gene-related peptide receptors. Front Mol Biosci. 2021;8:720561. 34513925 10.3389/fmolb.2021.720561 PMC8427520

[56] Barwell J, Wootten D, Simms J, Hay DL, Poyner DR. Ramps and cgrp receptors. Adv Exp Med Biol. 2012;744:13–24. 22434104 10.1007/978-1-4614-2364-5_2

[57] Zhu W, Sheng D, Shao Y, Zhang Q, Peng Y. Neuronal calcitonin gene-related peptide promotes prostate tumor growth in the bone microenvironment. Peptides. 2021;135:170423. 33086087 10.1016/j.peptides.2020.170423

[58] Venkatesh HS, Johung TB, Caretti V, Noll A, Tang Y, Nagaraja S, et al. Neuronal activity promotes glioma growth through neuroligin-3 secretion. Cell. 2015;161(4):803–16. 25913192 10.1016/j.cell.2015.04.012 PMC4447122

[59] Venkatesh HS, Tam LT, Woo PJ, Lennon J, Nagaraja S, Gillespie SM, et al. Targeting neuronal activity-regulated neuroligin-3 dependency in high-grade glioma. Nature. 2017;549(7673):533–7. 28959975 10.1038/nature24014 PMC5891832

[60] Khanmammadova N, Islam S, Sharma P, Amit M. Neuro-immune interactions and immuno-oncology. Trends Cancer. 2023;9(8):636–49. 37258398 10.1016/j.trecan.2023.05.002 PMC10524972

[61] Jin H, Li M, Jeong E, Castro-Martinez F, Zuker CS. A body-brain circuit that regulates body inflammatory responses. Nature. 2024;630(8017):695–703. 38692285 10.1038/s41586-024-07469-y PMC11186780

[62] Elenkov IJ, Wilder RL, Chrousos GP, Vizi ES. The sympathetic nerve–an integrative interface between two supersystems: the brain and the immune system. Pharmacol Rev. 2000;52(4):595–638. 11121511

[63] Huang S, Ziegler CGK, Austin J, Mannoun N, Vukovic M, Ordovas-Montanes J, et al. Lymph nodes are innervated by a unique population of sensory neurons with immunomodulatory potential. Cell. 2021;184(2):441–59.e25. 33333021 10.1016/j.cell.2020.11.028 PMC9612289

[64] Cole SW, Nagaraja AS, Lutgendorf SK, Green PA, Sood AK. Sympathetic nervous system regulation of the tumor microenvironment. Nat Rev Cancer. 2015;15(9):563–72. 26299593 10.1038/nrc3978 PMC4828959

[65] Thapa S, Cao X. Nervous regulation: beta-2-adrenergic signaling in immune homeostasis, cancer immunotherapy, and autoimmune diseases. Cancer Immunol Immunother. 2023;72(8):2549–56. 37060364 10.1007/s00262-023-03445-z PMC10693916

[66] Faulkner S, Jobling P, March B, Jiang CC, Hondermarck H. Tumor neurobiology and the war of nerves in cancer. Cancer Discov. 2019;9(6):702–10. 30944117 10.1158/2159-8290.CD-18-1398

[67] Dong Z, Hou L, Luo W, Pan LH, Li X, Tan HP, et al. Myocardial infarction drives trained immunity of monocytes, accelerating atherosclerosis. Eur Heart J. 2024;45(9):669–84. 38085922 10.1093/eurheartj/ehad787

[68] Godiyal Y, Maheshwari D, Taniguchi H, Zinzuwadia SS, Morera-Díaz Y, Tewari D, et al. Role of PD-1/PD-L1 signaling axis in oncogenesis and its targeting by bioactive natural compounds for cancer immunotherapy. Mil Med Res. 2024;11(1):82. 39690423 10.1186/s40779-024-00586-9 PMC11654217

[69] Dutta P, Courties G, Wei Y, Leuschner F, Gorbatov R, Robbins CS, et al. Myocardial infarction accelerates atherosclerosis. Nature. 2012;487(7407):325–9. 22763456 10.1038/nature11260 PMC3401326

[70] Campbell JP, Karolak MR, Ma Y, Perrien DS, Masood-Campbell SK, Penner NL, et al. Stimulation of host bone marrow stromal cells by sympathetic nerves promotes breast cancer bone metastasis in mice. PLoS Biol. 2012;10(7):e1001363. 22815651 10.1371/journal.pbio.1001363 PMC3398959

[71] Partecke LI, Käding A, Trung DN, Diedrich S, Sendler M, Weiss F, et al. Subdiaphragmatic vagotomy promotes tumor growth and reduces survival via TNFα in a murine pancreatic cancer model. Oncotarget. 2017;8(14):22501–12. 28160574 10.18632/oncotarget.15019 PMC5410240

[72] Renz BW, Tanaka T, Sunagawa M, Takahashi R, Jiang Z, Macchini M, et al. Cholinergic signaling via muscarinic receptors directly and indirectly suppresses pancreatic tumorigenesis and cancer stemness. Cancer Discov. 2018;8(11):1458–73. 30185628 10.1158/2159-8290.CD-18-0046 PMC6214763

[73] Renz BW, Takahashi R, Tanaka T, Macchini M, Hayakawa Y, Dantes Z, et al. Β2 adrenergic-neurotrophin feedforward loop promotes pancreatic cancer. Cancer Cell. 2018;33(1):75–90.e7. 29249692 10.1016/j.ccell.2017.11.007 PMC5760435

[74] Stopczynski RE, Normolle DP, Hartman DJ, Ying H, DeBerry JJ, Bielefeldt K, et al. Neuroplastic changes occur early in the development of pancreatic ductal adenocarcinoma. Cancer Res. 2014;74(6):1718–27. 24448244 10.1158/0008-5472.CAN-13-2050 PMC4036226

[75] Schwartz ES, La JH, Scheff NN, Davis BM, Albers KM, Gebhart GF. TRPV1 and TRPA1 antagonists prevent the transition of acute to chronic inflammation and pain in chronic pancreatitis. J Neurosci. 2013;33(13):5603–11. 23536075 10.1523/JNEUROSCI.1806-12.2013 PMC3690366

[76] James JM, Mukouyama YS. Neuronal action on the developing blood vessel pattern. Semin Cell Dev Biol. 2011;22(9):1019–27. 21978864 10.1016/j.semcdb.2011.09.010 PMC3230733

[77] Shalabi S, Belayachi A, Larrivée B. Involvement of neuronal factors in tumor angiogenesis and the shaping of the cancer microenvironment. Front Immunol. 2024;15:1284629. 38375479 10.3389/fimmu.2024.1284629 PMC10875004

[78] Zahalka AH, Arnal-Estapé A, Maryanovich M, Nakahara F, Cruz CD, Finley LWS, et al. Adrenergic nerves activate an angio-metabolic switch in prostate cancer. Science. 2017;358(6361):321–6. 29051371 10.1126/science.aah5072 PMC5783182

[79] Thaker PH, Han LY, Kamat AA, Arevalo JM, Takahashi R, Lu C, et al. Author correction: chronic stress promotes tumor growth and angiogenesis in a mouse model of ovarian carcinoma. Nat Med. 2021;27(12):2246. 34799731 10.1038/s41591-021-01566-5

[80] Li Y, Liu F, Cai Q, Deng L, Ouyang Q, Zhang XH, et al. Invasion and metastasis in cancer: molecular insights and therapeutic targets. Signal Transduct Target Ther. 2025;10(1):57. 39979279 10.1038/s41392-025-02148-4 PMC11842613

[81] Ly T, Harihar S, Welch DR. KISS1 in metastatic cancer research and treatment: potential and paradoxes. Cancer Metastasis Rev. 2020;39(3):739–54. 32152912 10.1007/s10555-020-09868-9 PMC7483209

[82] Kalpana G, Figy C, Yeung M, Yeung KC. Reduced rhoa expression enhances breast cancer metastasis with a concomitant increase in CCR5 and CXCR4 chemokines signaling. Sci Rep. 2019;9(1):16351. 31705019 10.1038/s41598-019-52746-w PMC6841971

[83] Goertzen CG, Dragan M, Turley E, Babwah AV, Bhattacharya M. *KISS1R* signaling promotes invadopodia formation in human breast cancer cell via β-arrestin2/ERK. Cell Signal. 2016;28(3):165–76. 26721186 10.1016/j.cellsig.2015.12.010

[84] Magnon C, Hall SJ, Lin J, Xue X, Gerber L, Freedland SJ, et al. Autonomic nerve development contributes to prostate cancer progression. Science. 2013;341(6142):1236361. 23846904 10.1126/science.1236361

[85] Zhao J, Cheng L, Yang J, Xu F, Qi W, Liao K, et al. Tumor-nerve interactions in cancer regulation and progression. Cancer Lett. 2025;612:217483. 39842496 10.1016/j.canlet.2025.217483

[86] Erin N, Zhao W, Bylander J, Chase G, Clawson G. Capsaicin-induced inactivation of sensory neurons promotes a more aggressive gene expression phenotype in breast cancer cells. Breast Cancer Res Treat. 2006;99(3):351–64. 16583263 10.1007/s10549-006-9219-7

[87] Yang DQ, Freund DM, Harris BR, Wang D, Cleary MP, Hegeman AD. Measuring relative utilization of aerobic glycolysis in breast cancer cells by positional isotopic discrimination. FEBS Lett. 2016;590(18):3179–87. 27531463 10.1002/1873-3468.12360 PMC5039094

[88] Caragher SP, Shireman JM, Huang M, Miska J, Atashi F, Baisiwala S, et al. Activation of dopamine receptor 2 prompts transcriptomic and metabolic plasticity in glioblastoma. J Neurosci. 2019;39(11):1982–93. 30651332 10.1523/JNEUROSCI.1589-18.2018 PMC6507082

[89] Pavlova NN, Zhu J, Thompson CB. The hallmarks of cancer metabolism: still emerging. Cell Metab. 2022;34(3):355–77. 35123658 10.1016/j.cmet.2022.01.007 PMC8891094

[90] Fitzgerald PJ. Norepinephrine release may play a critical role in the Warburg effect: an integrative model of tumorigenesis. Neoplasma. 2020;67(5):947–57. 32453599 10.4149/neo_2020_200422N432

[91] Qiao G, Chen M, Mohammadpour H, MacDonald CR, Bucsek MJ, Hylander BL, et al. Chronic adrenergic stress contributes to metabolic dysfunction and an exhausted phenotype in T cells in the tumor microenvironment. Cancer Immunol Res. 2021;9(6):651–64. 33762351 10.1158/2326-6066.CIR-20-0445 PMC8355045

[92] Lamboy-Caraballo R, Ortiz-Sanchez C, Acevedo-Santiago A, Matta J, Monteiro A, Armaiz-Pena G. Norepinephrine-induced DNA damage in ovarian cancer cells. Int J Mol Sci. 2020;21(6):2250. 32213975 10.3390/ijms21062250 PMC7139728

[93] Krishna S, Hervey-Jumper SL. Neural regulation of cancer: cancer-induced remodeling of the central nervous system. Adv Biol. 2022;6(9):e2200047. 10.1002/adbi.202200047 PMC10182823 35802914

[94] Venkatesh HS, Morishita W, Geraghty AC, Silverbush D, Gillespie SM, Arzt M, et al. Electrical and synaptic integration of glioma into neural circuits. Nature. 2019;573(7775):539–45. 31534222 10.1038/s41586-019-1563-y PMC7038898

[95] Venkataramani V, Tanev DI, Strahle C, Studier-Fischer A, Fankhauser L, Kessler T, et al. Glutamatergic synaptic input to glioma cells drives brain tumour progression. Nature. 2019;573(7775):532–8. 31534219 10.1038/s41586-019-1564-x

[96] Folkman J. Angiogenesis: an organizing principle for drug discovery?. Nat Rev Drug Discov. 2007;6(4):273–86. 17396134 10.1038/nrd2115

[97] Zeng Q, Michael IP, Zhang P, Saghafinia S, Knott G, Jiao W, et al. Synaptic proximity enables NMDAR signalling to promote brain metastasis. Nature. 2019;573(7775):526–31. 31534217 10.1038/s41586-019-1576-6 PMC6837873

[98] Tan R, Wang F, Zhou Y, Huang Z, An Z, Xu Y. Neural functions in cancer: data analyses and database construction. Front Genet. 2023;14:1062052. 36861131 10.3389/fgene.2023.1062052 PMC9968960

[99] Balood M, Ahmadi M, Eichwald T, Ahmadi A, Majdoubi A, Roversi K, et al. Nociceptor neurons affect cancer immunosurveillance. Nature. 2022;611(7935):405–12. 36323780 10.1038/s41586-022-05374-w PMC9646485

[100] Kamiya A, Hiyama T, Fujimura A, Yoshikawa S. Sympathetic and parasympathetic innervation in cancer: therapeutic implications. Clin Auton Res. 2021;31(2):165–78. 32926324 10.1007/s10286-020-00724-y

[101] Yu Y, Hai Y, Zhou H, Bao W, Hu X, Gao Y, et al. Mettl3 inhibition suppresses cell growth and survival in colorectal cancer via ASNS downregulation. J Cancer. 2024;15(15):4853–65. 39132158 10.7150/jca.96760 PMC11310885

[102] Wang W, Gong Z, Wang K, Tian M, Zhang Y, Li X, et al. Activation of the BMP2-SMAD1-CGRP pathway in dorsal root ganglia contributes to bone cancer pain in a rat model. Heliyon. 2024;10(6):e27350. 38496903 10.1016/j.heliyon.2024.e27350 PMC10944225

[103] van de Lisdonk D, Li B. The area postrema: a critical mediator of brain-body interactions. Genes Dev. 2024;38(17–20):793–7. 39362783 10.1101/gad.352276.124 PMC11535157

[104] Sun Q, van de Lisdonk D, Ferrer M, Gegenhuber B, Wu M, Park Y, et al. Area postrema neurons mediate interleukin-6 function in cancer cachexia. Nat Commun. 2024;15(1):4682. 38824130 10.1038/s41467-024-48971-1 PMC11144211

[105] Reavis HD, Gysler SM, McKenney GB, Knarr M, Lusk HJ, Rawat P, et al. Norepinephrine induces anoikis resistance in high-grade serous ovarian cancer precursor cells. JCI Insight. 2024;9(5):e170961. 38271085 10.1172/jci.insight.170961 PMC10972597

[106] Hou CH, Chen WL, Lin CY. Targeting nerve growth factor-mediated osteosarcoma metastasis: mechanistic insights and therapeutic opportunities using larotrectinib. Cell Death Dis. 2024;15(5):381. 38816365 10.1038/s41419-024-06752-0 PMC11139949

[107] He XY, Gao Y, Ng D, Michalopoulou E, George S, Adrover JM, et al. Chronic stress increases metastasis via neutrophil-mediated changes to the microenvironment. Cancer Cell. 2024;42(3):474–86.e12. 38402610 10.1016/j.ccell.2024.01.013 PMC11300849

[108] Cheng K, Pan J, Liu Q, Ji Y, Liu L, Guo X, et al. Exosomal lncRNA XIST promotes perineural invasion of pancreatic cancer cells via miR-211-5p/GDNF. Oncogene. 2024;43(18):1341–52. 38454138 10.1038/s41388-024-02994-6

[109] Xiong SY, Wen HZ, Dai LM, Lou YX, Wang ZQ, Yi YL, et al. A brain-tumor neural circuit controls breast cancer progression in mice. J Clin Invest. 2023;133(24):e167725. 37847562 10.1172/JCI167725 PMC10721160

[110] Wang X, Istvanffy R, Ye L, Teller S, Laschinger M, Diakopoulos KN, et al. Phenotype screens of murine pancreatic cancer identify a Tgf-α-CCL2-paxillin axis driving human-like neural invasion. J Clin Invest. 2023;133(21):e166333. 37607005 10.1172/JCI166333 PMC10617783

[111] Wang L, Duan Y, Lu S, Sun J. Magnetic nanomaterials mediate electromagnetic stimulations of nerves for applications in stem cell and cancer treatments. J Funct Biomater. 2023;14(2):58. 36826857 10.3390/jfb14020058 PMC9960824

[112] Taylor KR, Barron T, Hui A, Spitzer A, Yalçin B, Ivec AE, et al. Glioma synapses recruit mechanisms of adaptive plasticity. Nature. 2023;623(7986):366–74. 37914930 10.1038/s41586-023-06678-1 PMC10632140

[113] Li YT, Yuan WZ, Jin WL. Vagus innervation in the gastrointestinal tumor: current understanding and challenges. Biochimica et Biophysica Acta (BBA). 2023;1878(3):188884. 10.1016/j.bbcan.2023.188884 36990250

[114] Li F, He C, Yao H, Zhao Y, Ye X, Zhou S, et al. Glutamate from nerve cells promotes perineural invasion in pancreatic cancer by regulating tumor glycolysis through HK2 mRNA-m6A modification. Pharmacol Res. 2023;187:106555. 36403721 10.1016/j.phrs.2022.106555

[115] Huang D, Alexander PB, Li QJ, Wang XF. GABAergic signaling beyond synapses: an emerging target for cancer therapy. Trends Cell Biol. 2023;33(5):403–12. 36114091 10.1016/j.tcb.2022.08.004 PMC10008753

[116] Cui Q, Jiang D, Zhang Y, Chen C. The tumor-nerve circuit in breast cancer. Cancer Metastasis Rev. 2023;42(2):543–74. 36997828 10.1007/s10555-023-10095-1 PMC10349033

[117] Vats K, Kruglov O, Sahoo B, Soman V, Zhang J, Shurin GV, et al. Sensory nerves impede the formation of tertiary lymphoid structures and development of protective antimelanoma immune responses. Cancer Immunol Res. 2022;10(9):1141–54. 35834791 10.1158/2326-6066.CIR-22-0110 PMC10314799

[118] Tang PC, Chung JY, Liao J, Chan MK, Chan AS, Cheng G, et al. Single-cell RNA sequencing uncovers a neuron-like macrophage subset associated with cancer pain. Sci Adv. 2022;8(40):eabn5535. 36206343 10.1126/sciadv.abn5535 PMC9544324

[119] Numan T, Breedt LC, Maciel B, Kulik SD, Derks J, Schoonheim MM, et al. Regional healthy brain activity, glioma occurrence, and symptomatology. Brain. 2022;145(10):3654–65. 36130310 10.1093/brain/awac180 PMC9586543

[120] Chen P, Wang W, Liu R, Lyu J, Zhang L, Li B, et al. Olfactory sensory experience regulates gliomagenesis via neuronal IGF1. Nature. 2022;606(7914):550–6. 35545672 10.1038/s41586-022-04719-9

[121] Anastasaki C, Mo J, Chen JK, Chatterjee J, Pan Y, Scheaffer SM, et al. Neuronal hyperexcitability drives central and peripheral nervous system tumor progression in models of neurofibromatosis-1. Nat Commun. 2022;13(1):2785. 35589737 10.1038/s41467-022-30466-6 PMC9120229

[122] Zhang M, Zheng M, Dai L, Zhang WL, Fan HY, Yu XH, et al. CXCL12/CXCR4 facilitates perineural invasion via induction of the Twist/S100A4 axis in salivary adenoid cystic carcinoma. J Cell Mol Med. 2021;25(16):7901–12. 34170080 10.1111/jcmm.16713 PMC8358865

[123] Yoneda T, Hiasa M, Okui T, Hata K. Sensory nerves: a driver of the vicious cycle in bone metastasis?. J Bone Oncol. 2021;30:100387. 34504741 10.1016/j.jbo.2021.100387 PMC8411232

[124] Ren J, He J, Zhang H, Xia Y, Hu Z, Loughran P, et al. Platelet TLR4-ERK5 axis facilitates net-mediated capturing of circulating tumor cells and distant metastasis after surgical stress. Cancer Res. 2021;81(9):2373–85. 33687949 10.1158/0008-5472.CAN-20-3222 PMC8137664

[125] Liu S, Wang Z, Su Y, Qi L, Yang W, Fu M, et al. A neuroanatomical basis for electroacupuncture to drive the vagal-adrenal axis. Nature. 2021;598(7882):641–5. 34646018 10.1038/s41586-021-04001-4 PMC9178665

[126] De Logu F, Marini M, Landini L, Souza Monteiro de Araujo D, Bartalucci N, Trevisan G, et al. Peripheral nerve resident macrophages and schwann cells mediate cancer-induced pain. Cancer Res. 2021;81(12):3387–401. 33771895 10.1158/0008-5472.CAN-20-3326 PMC8260461

[127] Tian A, Kang B, Li B, Qiu B, Jiang W, Shao F, et al. Oncogenic state and cell identity combinatorially dictate the susceptibility of cells within glioma development hierarchy to IGF1R targeting. Adv Sci. 2020;7(21):2001724. 10.1002/advs.202001724 PMC7610337 33173731

[128] Middleton EA, He XY, Denorme F, Campbell RA, Ng D, Salvatore SP, et al. Neutrophil extracellular traps contribute to immunothrombosis in COVID-19 acute respiratory distress syndrome. Blood. 2020;136(10):1169–79. 32597954 10.1182/blood.2020007008 PMC7472714

[129] Guo X, Pan Y, Xiong M, Sanapala S, Anastasaki C, Cobb O, et al. Midkine activation of CD8^+^ T cells establishes a neuron-immune-cancer axis responsible for low-grade glioma growth. Nat Commun. 2020;11(1):2177. 32358581 10.1038/s41467-020-15770-3 PMC7195398

[130] Faulkner S, Griffin N, Rowe CW, Jobling P, Lombard JM, Oliveira SM, et al. Nerve growth factor and its receptor tyrosine kinase trka are overexpressed in cervical squamous cell carcinoma. FASEB Bioadv. 2020;2(7):398–408. 32676580 10.1096/fba.2020-00016 PMC7354692

[131] Cervantes-Villagrana RD, Albores-García D, Cervantes-Villagrana AR, García-Acevez SJ. Tumor-induced neurogenesis and immune evasion as targets of innovative anti-cancer therapies. Signal Transduct Target Ther. 2020;5(1):99. 32555170 10.1038/s41392-020-0205-z PMC7303203

[132] Zuazo-Gaztelu I, Pàez-Ribes M, Carrasco P, Martín L, Soler A, Martínez-Lozano M, et al. Antitumor effects of anti-semaphorin 4D antibody unravel a novel proinvasive mechanism of vascular-targeting agents. Cancer Res. 2019;79(20):5328–41. 31239269 10.1158/0008-5472.CAN-18-3436 PMC7611261

[133] Yang H, Xia L, Chen J, Zhang S, Martin V, Li Q, et al. Stress-glucocorticoid-TSC22D3 axis compromises therapy-induced antitumor immunity. Nat Med. 2019;25(9):1428–41. 31501614 10.1038/s41591-019-0566-4

[134] Wong SK, Mohamad NV, Giaze TR, Chin KY, Mohamed N, Ima-Nirwana S. Prostate cancer and bone metastases: the underlying mechanisms. Int J Mol Sci. 2019;20(10):2587. 31137764 10.3390/ijms20102587 PMC6567184

[135] Shurin GV, Kruglov O, Ding F, Lin Y, Hao X, Keskinov AA, et al. Melanoma-induced reprogramming of schwann cell signaling aids tumor growth. Cancer Res. 2019;79(10):2736–47. 30914431 10.1158/0008-5472.CAN-18-3872 PMC6522315

[136] Shuboni-Mulligan DD, Breton G, Smart D, Gilbert M, Armstrong TS. Radiation chronotherapy-clinical impact of treatment time-of-day: a systematic review. J Neurooncol. 2019;145(3):415–27. 31729636 10.1007/s11060-019-03332-7 PMC8130840

[137] Reinke H, Asher G. Crosstalk between metabolism and circadian clocks. Nat Rev Mol Cell Biol. 2019;20(4):227–41. 30635659 10.1038/s41580-018-0096-9

[138] Obradović MMS, Hamelin B, Manevski N, Couto JP, Sethi A, Coissieux MM, et al. Glucocorticoids promote breast cancer metastasis. Nature. 2019;567(7749):540–4. 30867597 10.1038/s41586-019-1019-4

[139] Martyn GV, Shurin GV, Keskinov AA, Bunimovich YL, Shurin MR. Schwann cells shape the neuro-immune environs and control cancer progression. Cancer Immunol Immunother. 2019;68(11):1819–29. 30607548 10.1007/s00262-018-02296-3 PMC11028256

[140] Wakabayashi H, Wakisaka S, Hiraga T, Hata K, Nishimura R, Tominaga M, et al. Decreased sensory nerve excitation and bone pain associated with mouse Lewis lung cancer in TRPV1-deficient mice. J Bone Miner Metab. 2018;36(3):274–85. 28516219 10.1007/s00774-017-0842-7

[141] Liu L, Zhu Y, Noë M, Li Q, Pasricha PJ. Neuronal transforming growth factor beta signaling via SMAD3 contributes to pain in animal models of chronic pancreatitis. Gastroenterology. 2018;154(8):2252–65.e2. 29505748 10.1053/j.gastro.2018.02.030 PMC5985212

[142] Zhang Y, Sui F, Ma J, Ren X, Guan H, Yang Q, et al. Positive feedback loops between NRCAM and major signaling pathways contribute to thyroid tumorigenesis. J Clin Endocrinol Metab. 2017;102(2):613–24. 27732334 10.1210/jc.2016-1677

[143] Maddocks ODK, Athineos D, Cheung EC, Lee P, Zhang T, van den Broek NJF, et al. Modulating the therapeutic response of tumours to dietary serine and glycine starvation. Nature. 2017;544(7650):372–6. 28425994 10.1038/nature22056

[144] Li Z, Dong J, Zou T, Du C, Li S, Chen C, et al. Dexamethasone induces docetaxel and cisplatin resistance partially through up-regulating Krüppel-like factor 5 in triple-negative breast cancer. Oncotarget. 2017;8(7):11555–65. 28030791 10.18632/oncotarget.14135 PMC5355285

[145] Hegemann M, Maas M, Rausch S, Walz S, Bedke J, Stenzl A, et al. Current concepts and trends in the treatment of bone metastases in patients with advanced prostate cancer. Asian J Androl. 2017;21(1):12–8. 29286005 10.4103/aja.aja_59_17 PMC6337943

[146] Cain DW, Cidlowski JA. Immune regulation by glucocorticoids. Nat Rev Immunol. 2017;17(4):233–47. 28192415 10.1038/nri.2017.1 PMC9761406

[147] Boerboom A, Dion V, Chariot A, Franzen R. Molecular mechanisms involved in schwann cell plasticity. Front Mol Neurosci. 2017;10:38. 28261057 10.3389/fnmol.2017.00038 PMC5314106

[148] Jessen KR, Mirsky R. The repair schwann cell and its function in regenerating nerves. J Physiol. 2016;594(13):3521–31. 26864683 10.1113/JP270874 PMC4929314

[149] Wculek SK, Malanchi I. Neutrophils support lung colonization of metastasis-initiating breast cancer cells. Nature. 2015;528(7582):413–7. 26649828 10.1038/nature16140 PMC4700594

[150] Kanojia D, Morshed RA, Zhang L, Miska JM, Qiao J, Kim JW, et al. Βiii-tubulin regulates breast cancer metastases to the brain. Mol Cancer Ther. 2015;14(5):1152–61. 25724666 10.1158/1535-7163.MCT-14-0950 PMC4425587

[151] Casbon AJ, Reynaud D, Park C, Khuc E, Gan DD, Schepers K, et al. Invasive breast cancer reprograms early myeloid differentiation in the bone marrow to generate immunosuppressive neutrophils. Proc Natl Acad Sci U S A. 2015;112(6):E566–75. 25624500 10.1073/pnas.1424927112 PMC4330753

[152] Campbell SL, Robel S, Cuddapah VA, Robert S, Buckingham SC, Kahle KT, et al. GABAergic disinhibition and impaired KCC2 cotransporter activity underlie tumor-associated epilepsy. Glia. 2015;63(1):23–36. 25066727 10.1002/glia.22730 PMC4237714

[153] Xiang HB, Liu C, Liu TT, Xiong J. Central circuits regulating the sympathetic outflow to lumbar muscles in spinally transected mice by retrograde transsynaptic transport. Int J Clin Exp Pathol. 2014;7(6):2987–97. 25031717 PMC4097212

[154] Neman J, Termini J, Wilczynski S, Vaidehi N, Choy C, Kowolik CM, et al. Human breast cancer metastases to the brain display GABAergic properties in the neural niche. Proc Natl Acad Sci U S A. 2014;111(3):984–9. 24395782 10.1073/pnas.1322098111 PMC3903266

[155] Marik PE, Bellomo R. Stress hyperglycemia: an essential survival response! Crit Care Med. 2013;41(6):e93–4. 23685597 10.1097/CCM.0b013e318283d124

[156] Flandreau EI, Bourke CH, Ressler KJ, Vale WW, Nemeroff CB, Owens MJ. Escitalopram alters gene expression and HPA axis reactivity in rats following chronic overexpression of corticotropin-releasing factor from the central amygdala. Psychoneuroendocrinology. 2013;38(8):1349–61. 23267723 10.1016/j.psyneuen.2012.11.020 PMC3749072

[157] Demir IE, Schorn S, Schremmer-Danninger E, Wang K, Kehl T, Giese NA, et al. Perineural mast cells are specifically enriched in pancreatic neuritis and neuropathic pain in pancreatic cancer and chronic pancreatitis. PLoS ONE. 2013;8(3):e60529. 23555989 10.1371/journal.pone.0060529 PMC3610867

[158] Cools-Lartigue J, Spicer J, McDonald B, Gowing S, Chow S, Giannias B, et al. Neutrophil extracellular traps sequester circulating tumor cells and promote metastasis. J Clin Invest. 2013;123(8):3446–58. 23863628 10.1172/JCI67484 PMC3726160

[159] Erin N, Duymuş O, Oztürk S, Demir N. Activation of vagus nerve by semapimod alters substance P levels and decreases breast cancer metastasis. Regul Pept. 2012;179(1–3):101–8. 22982142 10.1016/j.regpep.2012.08.001

[160] Adriaenssens E, Vanhecke E, Saule P, Mougel A, Page A, Romon R, et al. Nerve growth factor is a potential therapeutic target in breast cancer. Cancer Res. 2008;68(2):346–51. 18199526 10.1158/0008-5472.CAN-07-1183

[161] Sève P, Lai R, Ding K, Winton T, Butts C, Mackey J, et al. Class III beta-tubulin expression and benefit from adjuvant cisplatin/vinorelbine chemotherapy in operable non-small cell lung cancer: analysis of NCIC JBR.10. Clin Cancer Res. 2007;13(3):994–9. 17289895 10.1158/1078-0432.CCR-06-1503

[162] Anand U, Otto WR, Casula MA, Day NC, Davis JB, Bountra C, et al. The effect of neurotrophic factors on morphology, TRPV1 expression, and capsaicin responses of cultured human DRG sensory neurons. Neurosci Lett. 2006;399(1–2):51–6. 16481104 10.1016/j.neulet.2006.01.046

[163] Douma S, Van Laar T, Zevenhoven J, Meuwissen R, Van Garderen E, Peeper DS. Suppression of anoikis and induction of metastasis by the neurotrophic receptor TRKB. Nature. 2004;430(7003):1034–9. 15329723 10.1038/nature02765

[164] Ulloa L. Bioelectronic neuro-immunology: neuronal networks for sympathetic-splenic and vagal-adrenal control. Neuron. 2023;111(1):10–4. 36202096 10.1016/j.neuron.2022.09.015

[165] Holtkamp SJ, Ince LM, Barnoud C, Schmitt MT, Sinturel F, Pilorz V, et al. Circadian clocks guide dendritic cells into skin lymphatics. Nat Immunol. 2021;22(11):1375–81. 34663979 10.1038/s41590-021-01040-x PMC8553624

[166] Gomez A, Wu Y, Zhang C, Boyd L, Wee TL, Gewolb J, et al. A brain-body feedback loop driving HPA-axis dysfunction in breast cancer. bioRxiv. 2024.

[167] Zheng Y, Li L, Shen Z, Wang L, Niu X, Wei Y, et al. Mechanisms of neural infiltration-mediated tumor metabolic reprogramming impacting immunotherapy efficacy in non-small cell lung cancer. J Exp Clin Cancer Res. 2024;43(1):284. 39385213 10.1186/s13046-024-03202-9 PMC11465581

[168] Infante J, Burris H, Lewis N, Donehower R, Redman J, Friedman S, et al. A multicenter phase Ib study of the safety, pharmacokinetics, biological activity, and clinical efficacy of INCB7839, a potent and selective inhibitor of ADAM10 and ADAM17. Breast Cancer Res Treat. 2007;106.S269.

[169] Huang Q, Hu B, Zhang P, Yuan Y, Yue S, Chen X, et al. Neuroscience of cancer: unraveling the complex interplay between the nervous system, the tumor, and the tumor immune microenvironment. Mol Cancer. 2025;24(1):24. 39825376 10.1186/s12943-024-02219-0 PMC11740516

[170] Ye Y, Xie T, Amit M. Targeting the nerve-cancer circuit. Cancer Res. 2023;83(15):2445–7. 37470842 10.1158/0008-5472.CAN-23-1754

[171] He D, Manzoni A, Florentin D, Fisher W, Ding Y, Lee M, et al. Biologic effect of neurogenesis in pancreatic cancer. Hum Pathol. 2016;52:182–9. 26980040 10.1016/j.humpath.2016.02.001

[172] Lillemoe KD, Cameron JL, Kaufman HS, Yeo CJ, Pitt HA, Sauter PK. Chemical splanchnicectomy in patients with unresectable pancreatic cancer. A prospective randomized trial. Ann Surg. 1993;217(5):447–55. 7683868 10.1097/00000658-199305010-00004 PMC1242819

[173] Prazeres P, Leonel C, Silva WN, Rocha BGS, Santos GSP, Costa AC, et al. Ablation of sensory nerves favours melanoma progression. J Cell Mol Med. 2020;24(17):9574–89. 32691511 10.1111/jcmm.15381 PMC7520271

[174] Hayakawa Y, Sakitani K, Konishi M, Asfaha S, Niikura R, Tomita H, et al. Nerve growth factor promotes gastric tumorigenesis through aberrant cholinergic signaling. Cancer Cell. 2017;31(1):21–34. 27989802 10.1016/j.ccell.2016.11.005 PMC5225031

[175] Kline CL, Van den Heuvel AP, Allen JE, Prabhu VV, Dicker DT, El-Deiry WS. ONC201 kills solid tumor cells by triggering an integrated stress response dependent on ATF4 activation by specific eIF2α kinases. Sci Signal. 2016;9(415):ra18. 26884600 10.1126/scisignal.aac4374 PMC4968406

[176] Shi DD, Guo JA, Hoffman HI, Su J, Mino-Kenudson M, Barth JL, et al. Therapeutic avenues for cancer neuroscience: translational frontiers and clinical opportunities. Lancet Oncol. 2022;23(2):e62–74. 35114133 10.1016/S1470-2045(21)00596-9 PMC9516432

[177] Ding Y, Lee M, Gao Y, Bu P, Coarfa C, Miles B, et al. Neuropeptide Y nerve paracrine regulation of prostate cancer oncogenesis and therapy resistance. Prostate. 2021;81(1):58–71. 33022812 10.1002/pros.24081 PMC7756863

[178] Jung J, Zhang Y, Celiku O, Zhang W, Song H, Williams BJ, et al. Mitochondrial NIX promotes tumor survival in the hypoxic niche of glioblastoma. Cancer Res. 2019;79(20):5218–32. 31488423 10.1158/0008-5472.CAN-19-0198 PMC6801103

[179] Afridi S, Muzzammil M, Ali I, Shahi MH. Neuropeptide signaling in glioblastoma: a comprehensive review of the current state and future direction. Neuromolecular Med. 2025;27(1):27. 40227382 10.1007/s12017-025-08849-x

[180] Hetz C, Chevet E, Harding HP. Targeting the unfolded protein response in disease. Nat Rev Drug Discov. 2013;12(9):703–19. 23989796 10.1038/nrd3976

[181] Cascone T, McKenzie JA, Mbofung RM, Punt S, Wang Z, Xu C, et al. Increased tumor glycolysis characterizes immune resistance to adoptive T cell therapy. Cell Metab. 2018;27(5):977–87.e4. 29628419 10.1016/j.cmet.2018.02.024 PMC5932208

[182] Chang CH, Qiu J, O’Sullivan D, Buck MD, Noguchi T, Curtis JD, et al. Metabolic competition in the tumor microenvironment is a driver of cancer progression. Cell. 2015;162(6):1229–41. 26321679 10.1016/j.cell.2015.08.016 PMC4864363

[183] Guo Y, Li Y, Su P, Yan M, Wang M, Li S, et al. Tumor microtubes: a new potential therapeutic target for high-grade gliomas. J Neuropathol Exp Neurol. 2025;84(2):93–103. 39560360 10.1093/jnen/nlae119

[184] Osswald M, Jung E, Sahm F, Solecki G, Venkataramani V, Blaes J, et al. Brain tumour cells interconnect to a functional and resistant network. Nature. 2015;528(7580):93–8. 26536111 10.1038/nature16071

[185] Watson DC, Bayik D, Storevik S, Moreino SS, Sprowls SA, Han J, et al. GAP43-dependent mitochondria transfer from astrocytes enhances glioblastoma tumorigenicity. Nat Cancer. 2023;4(5):648–64. 37169842 10.1038/s43018-023-00556-5 PMC10212766

[186] Yang J, Wu Y, Lv X, Liu S, Yuan Z, Chen Y, et al. Neurotransmitters: an emerging target for therapeutic resistance to tumor immune checkpoint inhibitors. Mol Cancer. 2025;24(1):216. 40790592 10.1186/s12943-025-02413-8 PMC12337524

[187] Liu Y, Carlsson R, Ambjørn M, Hasan M, Badn W, Darabi A, et al. PD-L1 expression by neurons nearby tumors indicates better prognosis in glioblastoma patients. J Neurosci. 2013;33(35):14231–45. 23986257 10.1523/JNEUROSCI.5812-12.2013 PMC6618508

[188] Thielman NRJ, Funes V, Davuluri S, Ibanez HE, Sun WC, Fu J, et al. Semaphorin 3D promotes pancreatic ductal adenocarcinoma progression and metastasis through macrophage reprogramming. Sci Adv. 2024;10(42):eadp0684. 39413197 10.1126/sciadv.adp0684 PMC11801256

[189] Zhu J, Powis de Tenbossche CG, Cané S, Colau D, van Baren N, Lurquin C, et al. Resistance to cancer immunotherapy mediated by apoptosis of tumor-infiltrating lymphocytes. Nat Commun. 2017;8(1):1404. 29123081 10.1038/s41467-017-00784-1 PMC5680273

[190] Xiong D, Zhang L, Sun ZJ. Targeting the epigenome to reinvigorate T cells for cancer immunotherapy. Mil Med Res. 2023;10(1):59. 38044445 10.1186/s40779-023-00496-2 PMC10694991

[191] Sierra JR, Corso S, Caione L, Cepero V, Conrotto P, Cignetti A, et al. Tumor angiogenesis and progression are enhanced by sema4D produced by tumor-associated macrophages. J Exp Med. 2008;205(7):1673–85. 18559453 10.1084/jem.20072602 PMC2442644

[192] Tamagnone L, Franzolin G. Targeting semaphorin 4D in cancer: a look from different perspectives. Cancer Res. 2019;79(20):5146–8. 31615809 10.1158/0008-5472.CAN-19-2387

[193] Zhang B, Wang S, Fu Z, Gao Q, Yang L, Lei Z, et al. Single-cell RNA sequencing reveals intratumoral heterogeneity and potential mechanisms of malignant progression in prostate cancer with perineural invasion. Front Genet. 2022;13:1073232. 36712886 10.3389/fgene.2022.1073232 PMC9875799

[194] Chambers CR, Watakul S, Schofield P, Howell AE, Zhu J, Tran AMH, et al. Targeting the NPY/NPY1R signaling axis in mutant p53-dependent pancreatic cancer impairs metastasis. Sci Adv. 2025;11(11):eadq4416. 40073121 10.1126/sciadv.adq4416 PMC11900870

[195] Pascetta SA, Kirsh SM, Cameron M, Uniacke J. Pharmacological inhibition of neuropeptide Y receptors Y1 and Y5 reduces hypoxic breast cancer migration, proliferation, and signaling. BMC Cancer. 2023;23(1):494. 37264315 10.1186/s12885-023-10993-1 PMC10234023

[196] Shaikh M, Shirodkar S, Doshi G. Unraveling the role of perineural invasion in cancer progression across multiple tumor types. Med Oncol. 2025;42(8):283. 40569482 10.1007/s12032-025-02855-6

[197] Speyer CL, Hachem AH, Assi AA, Johnson JS, DeVries JA, Gorski DH. Metabotropic glutamate receptor-1 as a novel target for the antiangiogenic treatment of breast cancer. PLoS ONE. 2014;9(3):e88830. 24633367 10.1371/journal.pone.0088830 PMC3954556

[198] Hatipoglu G, Hock SW, Weiss R, Fan Z, Sehm T, Ghoochani A, et al. Sunitinib impedes brain tumor progression and reduces tumor-induced neurodegeneration in the microenvironment. Cancer Sci. 2015;106(2):160–70. 25458015 10.1111/cas.12580 PMC4399021

[199] Rabas N, Ferreira RMM, Di Blasio S, Malanchi I. Cancer-induced systemic pre-conditioning of distant organs: building a niche for metastatic cells. Nat Rev Cancer. 2024;24(12):829–49. 39390247 10.1038/s41568-024-00752-0

[200] Krishna S, Choudhury A, Keough MB, Seo K, Ni L, Kakaizada S, et al. Glioblastoma remodelling of human neural circuits decreases survival. Nature. 2023;617(7961):599–607. 37138086 10.1038/s41586-023-06036-1 PMC10191851

[201] Buckingham SC, Campbell SL, Haas BR, Montana V, Robel S, Ogunrinu T, et al. Glutamate release by primary brain tumors induces epileptic activity. Nat Med. 2011;17(10):1269–74. 21909104 10.1038/nm.2453 PMC3192231

[202] Calandre EP, Rico-Villademoros F, Slim M. Alpha_2_delta ligands, gabapentin, pregabalin, and mirogabalin: a review of their clinical pharmacology and therapeutic use. Expert Rev Neurother. 2016;16(11):1263–77. 27345098 10.1080/14737175.2016.1202764

[203] Weller M, Stupp R, Wick W. Epilepsy meets cancer: when, why, and what to do about it?. Lancet Oncol. 2012;13(9):e375–82. 22935237 10.1016/S1470-2045(12)70266-8

[204] Körber V, Yang J, Barah P, Wu Y, Stichel D, Gu Z, et al. Evolutionary trajectories of IDH^wt^ glioblastomas reveal a common path of early tumorigenesis instigated years ahead of initial diagnosis. Cancer Cell. 2019;35(4):692–704.e12. 30905762 10.1016/j.ccell.2019.02.007

[205] Huberfeld G, Vecht CJ. Seizures and gliomas–towards a single therapeutic approach. Nat Rev Neurol. 2016;12(4):204–16. 26965673 10.1038/nrneurol.2016.26

[206] Curry RN, Ma Q, McDonald MF, Ko Y, Srivastava S, Chin PS, et al. Integrated electrophysiological and genomic profiles of single cells reveal spiking tumor cells in human glioma. Cancer Cell. 2024;42(10):1713–28.e6. 39241781 10.1016/j.ccell.2024.08.009 PMC11479845

[207] Stevens NA, Drewa N, Venkataramani V. Spark in the darkness: discovering action potentials in brain tumors. Cancer Cell. 2024;42(10):1645–7. 39366373 10.1016/j.ccell.2024.09.004

[208] Kerkhof M, Benit C, Duran-Pena A, Vecht CJ. Seizures in oligodendroglial tumors. CNS Oncol. 2015;4(5):347–56. 26478444 10.2217/cns.15.29 PMC6082346

[209] Correia CE, Umemura Y, Flynn JR, Reiner AS, Avila EK. Pharmacoresistant seizures and IDH mutation in low-grade gliomas. Neurooncol Adv. 2021;3(1):vdab146. 34729486 10.1093/noajnl/vdab146 PMC8557671

[210] Bennett MI, Rayment C, Hjermstad M, Aass N, Caraceni A, Kaasa S. Prevalence and aetiology of neuropathic pain in cancer patients: a systematic review. Pain. 2012;153(2):359–65. 22115921 10.1016/j.pain.2011.10.028

[211] Schmitd LB, Scanlon CS, D’Silva NJ. Perineural invasion in head and neck cancer. J Dent Res. 2018;97(7):742–50. 29443582 10.1177/0022034518756297 PMC6728584

[212] Zajączkowska R, Kocot-Kępska M, Leppert W, Wordliczek J. Bone pain in cancer patients: mechanisms and current treatment. Int J Mol Sci. 2019;20(23):6047. 31801267 10.3390/ijms20236047 PMC6928918

[213] Shi RJ, Ke BW, Tang YL, Liang XH. Perineural invasion: a potential driver of cancer-induced pain. Biochem Pharmacol. 2023;215:115692. 37481133 10.1016/j.bcp.2023.115692

[214] Mittal A, Sagi V, Gupta M, Gupta K. Mast cell neural interactions in health and disease. Front Cell Neurosci. 2019;13:110. 30949032 10.3389/fncel.2019.00110 PMC6435484

[215] Gupta K, Harvima IT. Mast cell-neural interactions contribute to pain and itch. Immunol Rev. 2018;282(1):168–87. 29431216 10.1111/imr.12622 PMC5812374

[216] Jiang SH, Li RK, Liu DJ, Xue JL, Yu MH, Zhang S, et al. The genomic, transcriptomic, and immunological profiles of perineural invasion in pancreatic ductal adenocarcinoma. Sci China Life Sci. 2023;66(1):183–6. 35804220 10.1007/s11427-022-2146-5

[217] Baruch EN, Nagarajan P, Gleber-Netto FO, Rao X, Xie T, Akhter S, et al. Inflammation induced by tumor-associated nerves promotes resistance to anti-PD-1 therapy in cancer patients and is targetable by interleukin-6 blockade. Res Sq. 2023:rs.3.rs-3161761.

[218] Zhang Y, Sang R, Bao J, Jiang Z, Qian D, Zhou Y, et al. Schwann cell-derived CXCL2 contributes to cancer pain by modulating macrophage infiltration in a mouse breast cancer model. Brain Behav Immun. 2023;109:308–20. 36754246 10.1016/j.bbi.2023.02.004

[219] Zhang Z, Lv ZG, Lu M, Li H, Zhou J. Nerve-tumor crosstalk in tumor microenvironment: from tumor initiation and progression to clinical implications. Biochimica Biophysica Acta (BBA). 2024;1879(4):189121. 10.1016/j.bbcan.2024.189121 38796026

[220] Li H, Zhao L, Li J, Zhang K, Bai W, Chen Y. Neuron-like macrophage differentiation via the APOE-TREM2 axis contributes to chronic pain in nasopharyngeal carcinoma. Cell Biol Toxicol. 2025;41(1):86. 40392335 10.1007/s10565-025-10035-5 PMC12092482

[221] Hasegawa K, Okui T, Shimo T, Ibaragi S, Kawai H, Ryumon S, et al. Lactate transporter monocarboxylate transporter 4 induces bone pain in head and neck squamous cell carcinoma. Int J Mol Sci. 2018;19(11):3317. 30366393 10.3390/ijms19113317 PMC6274991

[222] Gao YM, Pei Y, Zhao FF, Wang L. Osteoclasts in osteosarcoma: mechanisms, interactions, and therapeutic prospects. Cancer Manag Res. 2023;15:1323–37. 38027241 10.2147/CMAR.S431213 PMC10661907

[223] Zhang X, Yuan X, Li X, Yu H, Wang T, Zhang C, et al. Sodium danshensu alleviates bone cancer pain by inhibiting the osteoclast differentiation and CGRP expression. Eur J Pharmacol. 2025;992:177296. 39900329 10.1016/j.ejphar.2025.177296

[224] Chen X, Gan Y, Au NPB, Ma CHE. Current understanding of the molecular mechanisms of chemotherapy-induced peripheral neuropathy. Front Mol Neurosci. 2024;17:1345811. 38660386 10.3389/fnmol.2024.1345811 PMC11039947

[225] Koyanagi M, Imai S, Matsumoto M, Iguma Y, Kawaguchi-Sakita N, Kotake T, et al. Pronociceptive roles of schwann cell-derived galectin-3 in taxane-induced peripheral neuropathy. Cancer Res. 2021;81(8):2207–19. 33608316 10.1158/0008-5472.CAN-20-2799

[226] Sadler KE, Moehring F, Shiers SI, Laskowski LJ, Mikesell AR, Plautz ZR, et al. Transient receptor potential canonical 5 mediates inflammatory mechanical and spontaneous pain in mice. Sci Transl Med. 2021;13(595):eabd7702. 34039739 10.1126/scitranslmed.abd7702 PMC8923002

[227] Meerschaert KA, Edwards BS, Epouhe AY, Jefferson B, Friedman R, Babyok OL, et al. Neuronally expressed PDL1, not PD1, suppresses acute nociception. Brain Behav Immun. 2022;106:233–46. 36089217 10.1016/j.bbi.2022.09.001 PMC10343937

[228] Wanderley CWS, Maganin AGM, Adjafre B, Mendes AS, Silva CEA, Quadros AU, et al. PD-1/PD-L1 inhibition enhances chemotherapy-induced neuropathic pain by suppressing neuroimmune antinociceptive signaling. Cancer Immunol Res. 2022;10(11):1299–308. 36083496 10.1158/2326-6066.CIR-22-0003

[229] Loprinzi CL, Lacchetti C, Bleeker J, Cavaletti G, Chauhan C, Hertz DL, et al. Prevention and management of chemotherapy-induced peripheral neuropathy in survivors of adult cancers: ASCO guideline update. J Clin Oncol. 2020;38(28):3325–48. 32663120 10.1200/JCO.20.01399

[230] Sugimoto M, Takagi T, Suzuki R, Konno N, Asama H, Sato Y, et al. Mirogabalin vs pregabalin for chemotherapy-induced peripheral neuropathy in pancreatic cancer patients. BMC Cancer. 2021;21(1):1319. 34886831 10.1186/s12885-021-09069-9 PMC8656082

[231] Gu I, Gregory E, Atwood C, Lee SO, Song YH. Exploring the role of metabolites in cancer and the associated nerve crosstalk. Nutrients. 2022;14(9):1722. 35565690 10.3390/nu14091722 PMC9103817

[232] Shankara Narayanan JSN, Frizzi K, Erdem S, Ray P, Jaroch D, Cox B, et al. Oxaliplatin-induced peripheral neuropathy can be minimized by pressurized regional intravascular delivery in an orthotopic murine pancreatic cancer model. Discov Oncol. 2022;13(1):21. 35384564 10.1007/s12672-022-00483-4 PMC8986945

[233] Chida Y, Hamer M, Wardle J, Steptoe A. Do stress-related psychosocial factors contribute to cancer incidence and survival?. Nat Clin Pract Oncol. 2008;5(8):466–75. 18493231 10.1038/ncponc1134

[234] Marzorati C, Voskanyan V, Sala D, Grasso R, Borgogni F, Pietrobon R, et al. Psychosocial factors associated with quality of life in cancer patients undergoing treatment: an umbrella review. Health Qual Life Outcomes. 2025;23(1):31. 40188134 10.1186/s12955-025-02357-z PMC11971742

[235] Yan J, Chen Y, Luo M, Hu X, Li H, Liu Q, et al. Chronic stress in solid tumor development: from mechanisms to interventions. J Biomed Sci. 2023;30(1):8. 36707854 10.1186/s12929-023-00903-9 PMC9883141

[236] Li J, Che M, Zhang B, Zhao K, Wan C, Yang K. The association between the neuroendocrine system and the tumor immune microenvironment: emerging directions for cancer immunotherapy. Biochimica Biophysica Acta (BBA). 2023;1878(6):189007. 10.1016/j.bbcan.2023.189007 37907132

[237] Khan A, Song M, Dong Z. Chronic stress: a fourth etiology in tumorigenesis?. Mol Cancer. 2025;24(1):196. 40676620 10.1186/s12943-025-02402-x PMC12269309

[238] Pundavela J, Roselli S, Faulkner S, Attia J, Scott RJ, Thorne RF, et al. Nerve fibers infiltrate the tumor microenvironment and are associated with nerve growth factor production and lymph node invasion in breast cancer. Mol Oncol. 2015;9(8):1626–35. 26009480 10.1016/j.molonc.2015.05.001 PMC5528785

[239] Ma Y, Kroemer G. The cancer-immune dialogue in the context of stress. Nat Rev Immunol. 2024;24(4):264–81. 37833492 10.1038/s41577-023-00949-8

[240] Stagl JM, Bouchard LC, Lechner SC, Blomberg BB, Gudenkauf LM, Jutagir DR, et al. Long-term psychological benefits of cognitive-behavioral stress management for women with breast cancer: 11-year follow-up of a randomized controlled trial. Cancer. 2015;121(11):1873–81. 25809235 10.1002/cncr.29076 PMC4441540

[241] Stagl JM, Lechner SC, Carver CS, Bouchard LC, Gudenkauf LM, Jutagir DR, et al. A randomized controlled trial of cognitive-behavioral stress management in breast cancer: survival and recurrence at 11-year follow-up. Breast Cancer Res Treat. 2015;154(2):319–28. 26518021 10.1007/s10549-015-3626-6 PMC5752103

[242] Anghel T, Melania BL, Costea I, Albai O, Marinca A, Levai CM, et al. Review of psychological interventions in oncology: current trends and future directions. Medicina (Kaunas). 2025;61(2):279. 40005396 10.3390/medicina61020279 PMC11857804

[243] Bower JE, Crosswell AD, Stanton AL, Crespi CM, Winston D, Arevalo J, et al. Mindfulness meditation for younger breast cancer survivors: a randomized controlled trial. Cancer. 2015;121(8):1231–40. 25537522 10.1002/cncr.29194 PMC4393338

[244] Cramer H, Lauche R, Klose P, Lange S, Langhorst J, Dobos GJ. Yoga for improving health-related quality of life, mental health and cancer-related symptoms in women diagnosed with breast cancer. Cochrane Database Syst Rev. 2017;1(1):Cd010802. 28045199 10.1002/14651858.CD010802.pub2 PMC6465041

[245] Schmid D, Leitzmann MF. Association between physical activity and mortality among breast cancer and colorectal cancer survivors: a systematic review and meta-analysis. Ann Oncol. 2014;25(7):1293–311. 24644304 10.1093/annonc/mdu012

[246] Zhang Y, Sun Y, Li D, Liu X, Fang C, Yang C, et al. Acupuncture for breast cancer: a systematic review and meta-analysis of patient-reported outcomes. Front Oncol. 2021;11:646315. 34178633 10.3389/fonc.2021.646315 PMC8222976

[247] Bozorgi A, Khazaei S, Khademi A, Khazaei M. Natural and herbal compounds targeting breast cancer, a review based on cancer stem cells. Iran J Basic Med Sci. 2020;23(8):970–83. 32952942 10.22038/ijbms.2020.43745.10270 PMC7478260

[248] Satpathi S, Gaurkar SS, Potdukhe A, Wanjari MB. Unveiling the role of hormonal imbalance in breast cancer development: a comprehensive review. Cureus. 2023;15(7):e41737. 37575755 10.7759/cureus.41737 PMC10415229

[249] Borniger JC, Walker Ii WH, Surbhi, Emmer KM, Zhang N, Zalenski AA, et al. A role for hypocretin/orexin in metabolic and sleep abnormalities in a mouse model of non-metastatic breast cancer. Cell Metab. 2018;28(1):118–295. 29805100 10.1016/j.cmet.2018.04.021 PMC6031468

[250] Wang YF, Dong ZK, Jin WL. Hijacking homeostasis: the brain-body neural circuitry in tumor pathogenesis and emerging therapeutic frontiers. Mol Cancer. 2025;24(1):206. 40713672 10.1186/s12943-025-02396-6 PMC12291512

[251] Pu T, Sun J, Ren G, Li H. Neuro-immune crosstalk in cancer: mechanisms and therapeutic implications. Signal Transduct Target Ther. 2025;10(1):176. 40456735 10.1038/s41392-025-02241-8 PMC12130251

[252] Papagiannakopoulos T, Bauer MR, Davidson SM, Heimann M, Subbaraj L, Bhutkar A, et al. Circadian rhythm disruption promotes lung tumorigenesis. Cell Metab. 2016;24(2):324–31. 27476975 10.1016/j.cmet.2016.07.001 PMC5367626

[253] Chen X, Geng Y, Wei G, He D, Lv J, Wen W, et al. Neural circuitries between the brain and peripheral solid tumors. Cancer Res. 2024;84(21):3509–21. 39226520 10.1158/0008-5472.CAN-24-1779 PMC11532784

[254] Wang Y, Dong Z, An Z, Jin W. Cancer cachexia: focus on cachexia factors and inter-organ communication. Chin Med J (Engl). 2024;137(1):44–62. 37968131 10.1097/CM9.0000000000002846 PMC10766315

[255] Jing H, Gao Y, Sun Z, Liu S. Recent advances in novel tumor immunotherapy strategies based on regulating the tumor microenvironment and immune checkpoints. Front Immunol. 2025;16:1529403. 40607438 10.3389/fimmu.2025.1529403 PMC12213813

[256] Seicol BJ, Guo Z, Garrity K, Xie R. Potential uses of auditory nerve stimulation to modulate immune responses in the inner ear and auditory brainstem. Front Integr Neurosci. 2023;17:1294525. 38162822 10.3389/fnint.2023.1294525 PMC10755874

[257] Brem S. Vagus nerve stimulation: novel concept for the treatment of glioblastoma and solid cancers by cytokine (interleukin-6) reduction, attenuating the sasp, enhancing tumor immunity. Brain Behav Immun. 2024;42:100859. 10.1016/j.bbih.2024.100859 PMC11541944 39512605

[258] Liu S, Wang ZF, Su YS, Ray RS, Jing XH, Wang YQ, et al. Somatotopic organization and intensity dependence in driving distinct NPY-expressing sympathetic pathways by electroacupuncture. Neuron. 2020;108(3):436–50.e7. 32791039 10.1016/j.neuron.2020.07.015 PMC7666081

[259] Torres-Rosas R, Yehia G, Peña G, Mishra P, del Rocio Thompson-Bonilla M, Moreno-Eutimio MA, et al. Dopamine mediates vagal modulation of the immune system by electroacupuncture. Nat Med. 2014;20(3):291–5. 24562381 10.1038/nm.3479 PMC3949155

[260] Li G, Jiang Y, Tong H, Liu J, Jiang Z, Zhao Y, et al. Sciatic nerve stimulation enhances NK cell cytotoxicity through dopamine signaling and synergizes immunotherapy in triple-negative breast cancer. Drug Resist Updat. 2025;79:101212. 39951881 10.1016/j.drup.2025.101212

[261] Dubeykovskaya Z, Si Y, Chen X, Worthley DL, Renz BW, Urbanska AM, et al. Neural innervation stimulates splenic TFF2 to arrest myeloid cell expansion and cancer. Nat Commun. 2016;7:10517. 26841680 10.1038/ncomms10517 PMC4742920

[262] Stolk RF, van der Pasch E, Naumann F, Schouwstra J, Bressers S, van Herwaarden AE, et al. Norepinephrine dysregulates the immune response and compromises host defense during sepsis. Am J Respir Crit Care Med. 2020;202(6):830–42. 32520577 10.1164/rccm.202002-0339OC

[263] Takashima Y, Hamano M, Yoshii K, Hayano A, Fukai J, Iwadate Y, et al. Reciprocal expression of the immune response genes CXCR3 and IFI44L as module hubs are associated with patient survivals in primary central nervous system lymphoma. Int J Clin Oncol. 2023;28(3):468–81. 36607476 10.1007/s10147-022-02285-8

[264] El-Sayes N, Vito A, Mossman K. Tumor heterogeneity: a great barrier in the age of cancer immunotherapy. Cancers (Basel). 2021;13(4):806. 33671881 10.3390/cancers13040806 PMC7918981

[265] Mayer EA, Nance K, Chen S. The gut-brain axis. Annu Rev Med. 2022;73:439–53. 34669431 10.1146/annurev-med-042320-014032

[266] Nuevo-Tapioles C, Santacatterina F, Stamatakis K, Núñez de Arenas C, Gómez de Cedrón M, Formentini L, et al. Coordinate β-adrenergic inhibition of mitochondrial activity and angiogenesis arrest tumor growth. Nat Commun. 2020;11(1):3606. 32681016 10.1038/s41467-020-17384-1 PMC7368041

[267] Jin MZ, Jin WL. The updated landscape of tumor microenvironment and drug repurposing. Signal Transduct Target Ther. 2020;5(1):166. 32843638 10.1038/s41392-020-00280-x PMC7447642

[268] Dong ZK, Wang YF, Li WP, Jin WL. Neurobiology of cancer: adrenergic signaling and drug repurposing. Pharmacol Ther. 2024;264:108750. 39527999 10.1016/j.pharmthera.2024.108750

[269] Mathews J, Newcomer JW, Mathews JR, Fales CL, Pierce KJ, Akers BK, et al. Neural correlates of weight gain with olanzapine. Arch Gen Psychiatry. 2012;69(12):1226–37. 22868896 10.1001/archgenpsychiatry.2012.934

[270] Zhao C, Li M. Neuroanatomical substrates of the disruptive effect of olanzapine on rat maternal behavior as revealed by c-Fos immunoreactivity. Pharmacol Biochem Behav. 2012;103(2):174–80. 22960130 10.1016/j.pbb.2012.08.021 PMC3494802

[271] Chen P, Hsu WH, Chang A, Tan Z, Lan Z, Zhou A, et al. Circadian regulator clock recruits immune-suppressive microglia into the GBM tumor microenvironment. Cancer Discov. 2020;10(3):371–81. 31919052 10.1158/2159-8290.CD-19-0400 PMC7058515

[272] Lu J, Zhang X, Su K, Luo H, Liu C, Yang Y, et al. Olanzapine suppresses MPFC activity-norepinephrine releasing to alleviate clock-enhanced cancer stemness under chronic stress. Cell Commun Signal. 2024;22(1):375. 39054537 10.1186/s12964-024-01747-y PMC11270788

[273] Sanomachi T, Suzuki S, Kuramoto K, Takeda H, Sakaki H, Togashi K, et al. Olanzapine, an atypical antipsychotic, inhibits survivin expression and sensitizes cancer cells to chemotherapeutic agents. Anticancer Res. 2017;37(11):6177–88. 29061799 10.21873/anticanres.12067

[274] Zhu Y, Zhao YF, Liu RS, Xiong YJ, Shen X, Wang Y, et al. Olanzapine induced autophagy through suppression of NF-κB activation in human glioma cells. CNS Neurosci Ther. 2019;25(9):911–21. 30955240 10.1111/cns.13127 PMC6698966

[275] Lee S, Weiss T, Bühler M, Mena J, Lottenbach Z, Wegmann R, et al. High-throughput identification of repurposable neuroactive drugs with potent anti-glioblastoma activity. Nat Med. 2024;30(11):3196–208. 39304781 10.1038/s41591-024-03224-y PMC11564103

[276] Venkataramani V, Yang Y, Schubert MC, Reyhan E, Tetzlaff SK, Wißmann N, et al. Glioblastoma hijacks neuronal mechanisms for brain invasion. Cell. 2022;185(16):2899-917.e31. 35914528 10.1016/j.cell.2022.06.054

[277] Weiss T, Schneider H, Silginer M, Steinle A, Pruschy M, Polić B, et al. NKG2D-dependent antitumor effects of chemotherapy and radiotherapy against glioblastoma. Clin Cancer Res. 2018;24(4):882–95. 29162646 10.1158/1078-0432.CCR-17-1766

[278] Langford DJ, Bailey AL, Chanda ML, Clarke SE, Drummond TE, Echols S, et al. Coding of facial expressions of pain in the laboratory mouse. Nat Methods. 2010;7(6):447–9. 20453868 10.1038/nmeth.1455

[279] Sheng M, Thompson MA, Greenberg ME. Creb: a Ca^2+^-regulated transcription factor phosphorylated by calmodulin-dependent kinases. Science. 1991;252(5011):1427–30. 1646483 10.1126/science.1646483

[280] Whitmarsh AJ, Davis RJ. Transcription factor AP-1 regulation by mitogen-activated protein kinase signal transduction pathways. J Mol Med (Berl). 1996;74(10):589–607. 8912180 10.1007/s001090050063

[281] Wang X, Li B, Kim YJ, Wang YC, Li Z, Yu J, et al. Targeting monoamine oxidase a for T cell-based cancer immunotherapy. Sci Immunol. 2021;6(59):eabh2383. 33990379 10.1126/sciimmunol.abh2383

[282] Coyle CM, Laws KR. The use of ketamine as an antidepressant: a systematic review and meta-analysis. Hum Psychopharmacol Clin Exp. 2015;30(3):152–63. 10.1002/hup.2475 25847818

[283] Machado-Vieira R, Henter ID, Zarate CA Jr. New targets for rapid antidepressant action. Prog Neurobiol. 2017;152:21–37. 26724279 10.1016/j.pneurobio.2015.12.001 PMC4919246

[284] Zhao J, Zhang R, Wang W, Jiang S, Liang H, Guo C, et al. Low-dose ketamine inhibits neuronal apoptosis and neuroinflammation in PC12 cells via α7nAChR mediated TLR4/MAPK/NF-κB signaling pathway. Int Immunopharmacol. 2023;117:109880. 36842233 10.1016/j.intimp.2023.109880

[285] Liu X, Liu X, Mao W, Guo Y, Bai N, Jin L, et al. *Tetrastigma* polysaccharide reprogramming of tumor-associated macrophages via PPARγ signaling pathway to play antitumor activity in breast cancer. J Ethnopharmacol. 2023;314:116645. 37196813 10.1016/j.jep.2023.116645

[286] Mehta AK, Kadel S, Townsend MG, Oliwa M, Guerriero JL. Macrophage biology and mechanisms of immune suppression in breast cancer. Front Immunol. 2021;12:643771. 33968034 10.3389/fimmu.2021.643771 PMC8102870

[287] Jiang Y, Li Y, Zhu B. T-cell exhaustion in the tumor microenvironment. Cell Death Dis. 2015;6(6):e1792. 26086965 10.1038/cddis.2015.162 PMC4669840

[288] Barron T, Yalçın B, Su M, Byun YG, Gavish A, Shamardani K, et al. Gabaergic neuron-to-glioma synapses in diffuse midline gliomas. Nature. 2025;639(8056):1060–8. 39972132 10.1038/s41586-024-08579-3 PMC11946904

[289] Ryu JY, Min KL, Chang MJ. Effect of anti-epileptic drugs on the survival of patients with glioblastoma multiforme: a retrospective, single-center study. PLoS ONE. 2019;14(12):e0225599. 31790459 10.1371/journal.pone.0225599 PMC6886804

[290] Gonzales CN, Negussie MB, Krishna S, Ambati VS, Hervey-Jumper SL. Malignant glioma remodeling of neuronal circuits: therapeutic opportunities and repurposing of antiepileptic drugs. Trends Cancer. 2024;10(12):1106–15. 39327186 10.1016/j.trecan.2024.09.003

[291] Albiñana V, Gallardo-Vara E, Casado-Vela J, Recio-Poveda L, Botella LM, Cuesta AM. Propranolol: a “pick and roll” team player in benign tumors and cancer therapies. J Clin Med. 2022;11(15):4539. 35956154 10.3390/jcm11154539 PMC9369479

[292] Ren L, Xu X, Liu X, Ning H, Ding Q, Yang M, et al. Recent advances in propranolol hydrochloride formulations for the treatment of infantile hemangiomas. Drug Des Devel Ther. 2025;19:1163–83. 39991089 10.2147/DDDT.S496847 PMC11846487

[293] Hiller JG, Cole SW, Crone EM, Byrne DJ, Shackleford DM, Pang JB, et al. Preoperative β-blockade with propranolol reduces biomarkers of metastasis in breast cancer: a phase II randomized trial. Clin Cancer Res. 2020;26(8):1803–11. 31754048 10.1158/1078-0432.CCR-19-2641

[294] Liao P, Song K, Zhu Z, Liu Z, Zhang W, Li W, et al. Propranolol suppresses the growth of colorectal cancer through simultaneously activating autologous CD8^+^ T cells and inhibiting tumor AKT/MAPK pathway. Clin Pharmacol Ther. 2020;108(3):606–15. 32418204 10.1002/cpt.1894

[295] Padmanaban V, Keller I, Seltzer ES, Ostendorf BN, Kerner Z, Tavazoie SF. Neuronal substance P drives metastasis through an extracellular RNA-TLR7 axis. Nature. 2024;633(8028):207–15. 39112700 10.1038/s41586-024-07767-5 PMC11633843

[296] Chandel V, Raj S, Kumar P, Gupta S, Dhasmana A, Kesari KK, et al. Metabolic regulation in HPV associated head and neck squamous cell carcinoma. Life Sci. 2020;258:118236. 32795537 10.1016/j.lfs.2020.118236

[297] Wilkie MD, Lau AS, Vlatkovic N, Jones TM, Boyd MT. Metabolic signature of squamous cell carcinoma of the head and neck: consequences of TP53 mutation and therapeutic perspectives. Oral Oncol. 2018;83:1–10. 30098763 10.1016/j.oraloncology.2018.05.018

[298] Shin JM, Kamarajan P, Fenno JC, Rickard AH, Kapila YL. Metabolomics of head and neck cancer: a mini-review. Front Physiol. 2016;7:526. 27877135 10.3389/fphys.2016.00526 PMC5099236

[299] Voelxen NF, Blatt S, Knopf P, Henkel M, Appelhans C, Righesso LAR, et al. Comparative metabolic analysis in head and neck cancer and the normal gingiva. Clin Oral Investig. 2018;22(2):1033–43. 28735466 10.1007/s00784-017-2185-0

[300] Zhang Y, Lin C, Liu Z, Sun Y, Chen M, Guo Y, et al. Cancer cells co-opt nociceptive nerves to thrive in nutrient-poor environments and upon nutrient-starvation therapies. Cell Metab. 2022;34(12):1999–2017.e10. 36395769 10.1016/j.cmet.2022.10.012

[301] Lu H, Zhang H, Weng ML, Zhang J, Jiang N, Cata JP, et al. Morphine promotes tumorigenesis and cetuximab resistance via EGFR signaling activation in human colorectal cancer. J Cell Physiol. 2021;236(6):4445–54. 33184860 10.1002/jcp.30161

[302] Lennon FE, Mirzapoiazova T, Mambetsariev B, Poroyko VA, Salgia R, Moss J, et al. The mu opioid receptor promotes opioid and growth factor-induced proliferation, migration, and epithelial mesenchymal transition (EMT) in human lung cancer. PLoS ONE. 2014;9(3):e91577. 24662916 10.1371/journal.pone.0091577 PMC3963855

[303] Schito L. Hypoxia-dependent angiogenesis and lymphangiogenesis in cancer. Adv Exp Med Biol. 2019;1136:71–85. 31201717 10.1007/978-3-030-12734-3_5

[304] Xia Y, Sun M, Huang H, Jin WL. Drug repurposing for cancer therapy. Signal Transduct Target Ther. 2024;9(1):92. 38637540 10.1038/s41392-024-01808-1 PMC11026526

[305] Chehelgerdi M, Chehelgerdi M, Allela OQB, Pecho RDC, Jayasankar N, Rao DP, et al. Progressing nanotechnology to improve targeted cancer treatment: overcoming hurdles in its clinical implementation. Mol Cancer. 2023;22(1):169. 37814270 10.1186/s12943-023-01865-0 PMC10561438

[306] Lei Y, Hamada Y, Li J, Cong L, Wang N, Li Y, et al. Targeted tumor delivery and controlled release of neuronal drugs with ferritin nanoparticles to regulate pancreatic cancer progression. J Control Release. 2016;232:131–42. 27046157 10.1016/j.jconrel.2016.03.023

[307] Chu X, Zhuang H, Liu Y, Li J, Wang Y, Jiang Y, et al. Blocking cancer-nerve crosstalk for treatment of metastatic bone cancer pain. Adv Mater. 2022;34(17):e2108653. 35244228 10.1002/adma.202108653

[308] Sun J, Wang D, Wei Y, Wang D, Ji Z, Sun W, et al. Capsaicin-induced Ca^2+^ overload and ablation of TRPV1-expressing axonal terminals for comfortable tumor immunotherapy. Nanoscale. 2024;17(6):3288–305. 10.1039/d4nr04454a 39688368

[309] Bhansali D, Tu NH, Inoue K, Teng S, Li T, Tran HD, et al. PAR_2_ on oral cancer cells and nociceptors contributes to oral cancer pain that can be relieved by nanoparticle-encapsulated AZ3451. Biomaterials. 2025;314:122874. 39418848 10.1016/j.biomaterials.2024.122874 PMC12519818

[310] Xu Y, Xu C, Song H, Feng X, Ma L, Zhang X, et al. Biomimetic bone-periosteum scaffold for spatiotemporal regulated innervated bone regeneration and therapy of osteosarcoma. J Nanobiotechnology. 2024;22(1):250. 38750519 10.1186/s12951-024-02430-7 PMC11094931

[311] Zhang Q, Zhuang H, Wen X, Su Y, Wang J, Qin H, et al. Organelle remodeling enhances mitochondrial ATP disruption for blocking neuro-pain signaling in bone tumor therapy. Chem Eng J. 2025:159825.

[312] Qin J, Liu J, Wei Z, Li X, Chen Z, Li J, et al. Targeted intervention in nerve-cancer crosstalk enhances pancreatic cancer chemotherapy. Nat Nanotechnol. 2024;20(2):311–24. 39496914 10.1038/s41565-024-01803-1

[313] Liang F, You Q, Yu B, Wang C, Yang Y, Zhu L, et al. Neurotransmitter-mimicking nanovesicles facilitate postoperative glioblastoma stem cell-specific treatment for preventing tumor recurrence. Adv Sci (Weinh). 2024:e2409713. 10.1002/advs.202409713 PMC11831431 39721005

[314] Ding L, Tang S, Yu A, Wang A, Tang W, Jia H, et al. Nanoemulsion-assisted siRNA delivery to modulate the nervous tumor microenvironment in the treatment of pancreatic cancer. ACS Appl Mater Interfaces. 2022;14(8):10015–29. 35188730 10.1021/acsami.1c21997 PMC9153289

[315] Ma GL, Lin WF. Immune checkpoint inhibition mediated with liposomal nanomedicine for cancer therapy. Mil Med Res. 2023;10(1):20. 37106400 10.1186/s40779-023-00455-x PMC10142459

[316] Kaduri M, Sela M, Kagan S, Poley M, Abumanhal-Masarweh H, Mora-Raimundo P, et al. Targeting neurons in the tumor microenvironment with bupivacaine nanoparticles reduces breast cancer progression and metastases. Sci Adv. 2021;7(41):eabj5435. 34613777 10.1126/sciadv.abj5435 PMC8494443

[317] He J, Zhang X, Xing H, Tan J, Zhang L, Xu Z, et al. Nanoparticle-mediated synergistic disruption of tumor innervation and redox homeostasis for potent antineoplastic therapy. J Control Release. 2024;376:457–69. 39437966 10.1016/j.jconrel.2024.10.039

[318] Zhou S, Li J, Yu J, Wang Y, Wang Z, He Z, et al. Tumor microenvironment adrenergic nerves blockade liposomes for cancer therapy. J Control Release. 2022;351:656–66. 36183971 10.1016/j.jconrel.2022.09.049

[319] Yang C, He Y, Chen F, Zhang F, Shao D, Wang Z. Leveraging β-adrenergic receptor signaling blockade for improved cancer immunotherapy through biomimetic nanovaccine. Small. 2023;19(14):e2207029. 36703529 10.1002/smll.202207029

[320] Singh AK, McGuirk JP. CAR T cells: continuation in a revolution of immunotherapy. Lancet Oncol. 2020;21(3):e168–78. 32135120 10.1016/S1470-2045(19)30823-X

[321] Yang P, Chen X, Yu F, Wang L, Li M, Bai Z, et al. CAR T cells secreting ngf-neutralizing SCFV enhance efficacy in clear cell renal cell carcinoma by relieving immunosuppression through immunosympathectomy. J Immunother Cancer. 2024;12(12):e009910. 39653553 10.1136/jitc-2024-009910 PMC11629019

[322] Branella GM, Spencer HT. Natural receptor- and ligand-based chimeric antigen receptors: strategies using natural ligands and receptors for targeted cell killing. Cells. 2021;11(1):21. 35011583 10.3390/cells11010021 PMC8750724

[323] Ramírez-Chacón A, Betriu-Méndez S, Bartoló-Ibars A, González A, Martí M, Juan M. Corrigendum: ligand-based CAR T-cell: different strategies to drive T-cells in future new treatments. Front Immunol. 2022;13:1078003. 36479103 10.3389/fimmu.2022.1078003 PMC9721137

[324] Khotskaya YB, Holla VR, Farago AF, Mills Shaw KR, Meric-Bernstam F, Hong DS. Targeting TRK family proteins in cancer. Pharmacol Ther. 2017;173:58–66. 28174090 10.1016/j.pharmthera.2017.02.006

[325] Liang D, Tang J, Sun B, He S, Yang D, Ma H, et al. Novel CAR-T cells targeting TRKB for the treatment of solid cancer. Apoptosis. 2024;29(11–12):2183–96. 38498249 10.1007/s10495-024-01936-7

[326] Chokshi CR, Shaikh MV, Brakel B, Rossotti MA, Tieu D, Maich W, et al. Targeting axonal guidance dependencies in glioblastoma with ROBO1 CAR T cells. Nat Med. 2024;30(10):2936–46. 39095594 10.1038/s41591-024-03138-9

[327] Di Carlo E, Sorrentino C. State of the ART CRISPR-based strategies for cancer diagnostics and treatment. Biomark Res. 2024;12(1):156. 39696697 10.1186/s40364-024-00701-x PMC11657220

[328] Galappaththi SL, Katz B, Howze PHt, Hoover G, Grelet S. A CRISPR/Cas9-based assay for high-throughput studies of cancer-induced innervation. Cancers (Basel). 2023;15(7):2026. 37046688 10.3390/cancers15072026 PMC10093009

[329] Sun Q, van de Lisdonk D, Ferrer M, Gegenhuber B, Wu M, Tollkuhn J, et al. Area postrema neurons mediate interleukin-6 function in cancer-associated cachexia. bioRxiv. 2023:2023.01.12.523716. 10.1038/s41467-024-48971-1 PMC11144211 38824130

[330] Fang N, Gu T, Wang Y, Wang S, Wang F, An Y, et al. Expression of pten-long mediated by CRISPR/Cas9 can repress U87 cell proliferation. J Cell Mol Med. 2017;21(12):3337–46. 28631420 10.1111/jcmm.13236 PMC5706501

[331] Lino CA, Harper JC, Carney JP, Timlin JA. Delivering CRISPR: a review of the challenges and approaches. Drug Deliv. 2018;25(1):1234–57. 29801422 10.1080/10717544.2018.1474964 PMC6058482

[332] Pineda M, Lear A, Collins JP, Kiani S. Safe CRISPR: challenges and possible solutions. Trends Biotechnol. 2019;37(4):389–401. 30352704 10.1016/j.tibtech.2018.09.010

[333] Bedbrook CN, Deverman BE, Gradinaru V. Viral strategies for targeting the central and peripheral nervous systems. Annu Rev Neurosci. 2018;41:323–48. 29709207 10.1146/annurev-neuro-080317-062048

[334] Zhong J, Xing X, Gao Y, Pei L, Lu C, Sun H, et al. Distinct roles of TREM2 in central nervous system cancers and peripheral cancers. Cancer Cell. 2024;42(6):968–84.e9. 38788719 10.1016/j.ccell.2024.05.001

[335] Smith TL, Yuan Z, Cardó-Vila M, Sanchez Claros C, Adem A, Cui MH, et al. AAVP displaying octreotide for ligand-directed therapeutic transgene delivery in neuroendocrine tumors of the pancreas. Proc Natl Acad Sci U S A. 2016;113(9):2466–71. 26884209 10.1073/pnas.1525709113 PMC4780640

[336] Wang H, Zheng Q, Lu Z, Wang L, Ding L, Xia L, et al. Role of the nervous system in cancers: a review. Cell Death Discov. 2021;7(1):76. 33846291 10.1038/s41420-021-00450-y PMC8041826

[337] Schmitd LB, Perez-Pacheco C, D’Silva NJ. Nerve density in cancer: less is better. FASEB Bioadv. 2021;3(10):773–86. 34632313 10.1096/fba.2021-00046 PMC8493966

[338] Perez-Pacheco C, Schmitd LB, Furgal A, Bellile EL, Liu M, Fattah A, et al. Increased nerve density adversely affects outcome in oral cancer. Clin Cancer Res. 2023;29(13):2501–12. 37039710 10.1158/1078-0432.CCR-22-3496 PMC10371054

[339] Schmitd LB, Perez-Pacheco C, Bellile EL, Wu W, Casper K, Mierzwa M, et al. Spatial and transcriptomic analysis of perineural invasion in oral cancer. Clin Cancer Res. 2022;28(16):3557–72. 35819260 10.1158/1078-0432.CCR-21-4543 PMC9560986

[340] Heij LR, Tan X, Kather JN, Niehues JM, Sivakumar S, Heussen N, et al. Nerve fibers in the tumor microenvironment are co-localized with lymphoid aggregates in pancreatic cancer. J Clin Med. 2021;10(3):490. 33573277 10.3390/jcm10030490 PMC7866811

[341] Tan X, Bednarsch J, Rosin M, Appinger S, Liu D, Wiltberger G, et al. PD-1^+^ T-cells correlate with nerve fiber density as a prognostic biomarker in patients with resected perihilar cholangiocarcinoma. Cancers (Basel). 2022. 10.3390/cancers14092190. 35565318 10.3390/cancers14092190 PMC9103905

[342] Olar A, He D, Florentin D, Ding Y, Wheeler T, Ayala G. Biological correlates of prostate cancer perineural invasion diameter. Hum Pathol. 2014;45(7):1365–9. 24768607 10.1016/j.humpath.2014.02.011 PMC4492300

[343] Olar A, He D, Florentin D, Ding Y, Ayala G. Biologic correlates and significance of axonogenesis in prostate cancer. Hum Pathol. 2014;45(7):1358–64. 24767770 10.1016/j.humpath.2014.02.009 PMC4492299

[344] Reeves FA, Battye S, Roth H, Peters JS, Hovens C, Costello AJ, et al. Prostatic nerve subtypes independently predict biochemical recurrence in prostate cancer. J Clin Neurosci. 2019;63:213–9. 30772200 10.1016/j.jocn.2019.01.052

[345] Iwasaki T, Hiraoka N, Ino Y, Nakajima K, Kishi Y, Nara S, et al. Reduction of intrapancreatic neural density in cancer tissue predicts poorer outcome in pancreatic ductal carcinoma. Cancer Sci. 2019;110(4):1491–502. 30776178 10.1111/cas.13975 PMC6447831

[346] Dagogo-Jack I, Shaw AT. Tumour heterogeneity and resistance to cancer therapies. Nat Rev Clin Oncol. 2018;15(2):81–94. 29115304 10.1038/nrclinonc.2017.166

[347] Zhu T, Wang T, Feng Z, Gao F, Zhang J, Jin C, et al. Glia maturation factor β as a novel independent prognostic biomarker and potential therapeutic target of kidney renal clear cell carcinoma. Front Oncol. 2022;12:880100. 35860559 10.3389/fonc.2022.880100 PMC9292986

[348] Zhang L, Deng Y, Yang J, Deng W, Li L. Neurotransmitter receptor-related gene signature as potential prognostic and therapeutic biomarkers in colorectal cancer. Front Cell Dev Biol. 2023;11:1202193. 38099288 10.3389/fcell.2023.1202193 PMC10720326

[349] Seyhan AA. Circulating liquid biopsy biomarkers in glioblastoma: advances and challenges. Int J Mol Sci. 2024;25(14):7974. 39063215 10.3390/ijms25147974 PMC11277426

[350] Santangelo A, Tamanini A, Cabrini G, Dechecchi MC. Circulating micrornas as emerging non-invasive biomarkers for gliomas. Ann Transl Med. 2017;5(13):277. 28758103 10.21037/atm.2017.06.15 PMC5515812

[351] Lu WL, Kuang H, Gu J, Hu X, Chen B, Fan Y. Gap-43 targeted indocyanine green-loaded near-infrared fluorescent probe for real-time mapping of perineural invasion lesions in pancreatic cancer *in vivo*. Nanomedicine. 2023;50:102671. 37054805 10.1016/j.nano.2023.102671

[352] Grey N, Silosky M, Lieu CH, Chin BB. Current status and future of targeted peptide receptor radionuclide positron emission tomography imaging and therapy of gastroenteropancreatic-neuroendocrine tumors. World J Gastroenterol. 2022;28(17):1768–80. 35633909 10.3748/wjg.v28.i17.1768 PMC9099199

[353] Fani M, Maecke HR, Okarvi SM. Radiolabeled peptides: valuable tools for the detection and treatment of cancer. Theranostics. 2012;2(5):481–501. 22737187 10.7150/thno.4024 PMC3364555

[354] Virgolini I, Raderer M, Kurtaran A, Angelberger P, Banyai S, Yang Q, et al. Vasoactive intestinal peptide-receptor imaging for the localization of intestinal adenocarcinomas and endocrine tumors. N Engl J Med. 1994;331(17):1116–21. 7935635 10.1056/NEJM199410273311703

[355] Reubi JC. Peptide receptors as molecular targets for cancer diagnosis and therapy. Endocr Rev. 2003;24(4):389–427. 12920149 10.1210/er.2002-0007

[356] Rangger C, Helbok A, Ocak M, Radolf T, Andreae F, Virgolini IJ, et al. Design and evaluation of novel radiolabelled vip derivatives for tumour targeting. Anticancer Res. 2013;33(4):1537–46. 23564795

[357] You H, Shang W, Min X, Weinreb J, Li Q, Leapman M, et al. Sight and switch off: nerve density visualization for interventions targeting nerves in prostate cancer. Sci Adv. 2020;6(6):eaax6040. 32076639 10.1126/sciadv.aax6040 PMC7002130

[358] Gonzalez P, Debnath S, Chen YA, Hernandez E, Jha P, Dakanali M, et al. A theranostic small-molecule prodrug conjugate for neuroendocrine prostate cancer. Pharmaceutics. 2023;15(2):481. 36839802 10.3390/pharmaceutics15020481 PMC9967013

[359] Shi HX, Tao HT, He JJ, Zhu FY, Xie CQ, Cheng YN, et al. Targeting DKK1 enhances the antitumor activity of paclitaxel and alleviates chemotherapy-induced peripheral neuropathy in breast cancer. Mol Cancer. 2024;23(1):152. 39085861 10.1186/s12943-024-02067-y PMC11290233

[360] Uccello TP, Lesch ML, Ullman NA, Kintzel SA, Gradzewicz LB, Velagaleti T, et al. Radiation therapy exacerbates tumor-promoting innervation and nerve signaling in rectal cancer. Int J Radiat Oncol Biol Phys. 2023;115(3):733–45. 36202180 10.1016/j.ijrobp.2022.09.080 PMC9898185

[361] Tetzlaff SK, Reyhan E, Layer N, Bengtson CP, Heuer A, Schroers J, et al. Characterizing and targeting glioblastoma neuron-tumor networks with retrograde tracing. Cell. 2024;188(2):390–411.e36. 39644898 10.1016/j.cell.2024.11.002

[362] Kwak S, Lee JY, Kim MJ, Lee HJ, Lee DK, Kang J, et al. Combination of PD-1 checkpoint blockade and botulinum toxin type A1 improves antitumor responses in mouse tumor models of melanoma and colon carcinoma. Immunol Invest. 2023;52(6):749–66. 37403798 10.1080/08820139.2023.2232403

[363] Zhang J, Liu J, Yue Y, Wang L, He Q, Xu S, et al. The immunotoxin targeting PRLR increases tamoxifen sensitivity and enhances the efficacy of chemotherapy in breast cancer. J Exp Clin Cancer Res. 2024;43(1):173. 38898487 10.1186/s13046-024-03099-4 PMC11188579

[364] Fang X, Ding H, Chen Y, Wang Q, Yuan X, Zhang C, et al. Wireless optogenetic targeting nociceptors helps host cells win the competitive colonization in implant-associated infections. Small Methods. 2024;8(12):e2400216. 39087367 10.1002/smtd.202400216

[365] Watanabe M, Narita M, Hamada Y, Yamashita A, Tamura H, Ikegami D, et al. Activation of ventral tegmental area dopaminergic neurons reverses pathological allodynia resulting from nerve injury or bone cancer. Mol Pain. 2018;14:1744806918756406. 29357732 10.1177/1744806918756406 PMC5802605

[366] Xu XR, Xiao Q, Hong YC, Liu YH, Liu Y, Tu J. Activation of dopaminergic VTA inputs to the mpfc ameliorates chronic stress-induced breast tumor progression. CNS Neurosci Ther. 2021;27(2):206–19. 33112032 10.1111/cns.13465 PMC7816210

[367] Zhang QR, Wang SX, Chen R. Integrated bioelectronic and optogenetic methods to study brain-body circuits. ACS Nano. 2024;18(44):30117–22. 39443299 10.1021/acsnano.4c07256 PMC11544702

[368] Deng Y, Xiao M, Wan AH, Li J, Sun L, Liang H, et al. RNA and RNA derivatives: light and dark sides in cancer immunotherapy. Antioxid Redox Signal. 2022;37(16–18):1266–90. 35369726 10.1089/ars.2022.0035

[369] Wang Y, Zhang L, Xu Z, Miao L, Huang L. MRNA vaccine with antigen-specific checkpoint blockade induces an enhanced immune response against established melanoma. Mol Ther. 2018;26(2):420–34. 29249397 10.1016/j.ymthe.2017.11.009 PMC5835019

[370] Sun Q, Hong Z, Zhang C, Wang L, Han Z, Ma D. Immune checkpoint therapy for solid tumours: clinical dilemmas and future trends. Signal Transduct Target Ther. 2023;8(1):320. 37635168 10.1038/s41392-023-01522-4 PMC10460796

[371] Kon E, Ad-El N, Hazan-Halevy I, Stotsky-Oterin L, Peer D. Targeting cancer with mRNA-lipid nanoparticles: key considerations and future prospects. Nat Rev Clin Oncol. 2023;20(11):739–54. 37587254 10.1038/s41571-023-00811-9

[372] Maryanovich M, Zahalka AH, Pierce H, Pinho S, Nakahara F, Asada N, et al. Adrenergic nerve degeneration in bone marrow drives aging of the hematopoietic stem cell niche. Nat Med. 2018;24(6):782–91. 29736022 10.1038/s41591-018-0030-x PMC6095812

[373] Zhang Y, Liu X, Shen T, Wang Q, Zhou S, Yang S, et al. Small circular RNAs as vaccines for cancer immunotherapy. Nat Biomed Eng. 2025;9(2):249–67. 39920212 10.1038/s41551-025-01344-5 PMC12100636

[374] Lian X, Chatterjee S, Sun Y, Dilliard SA, Moore S, Xiao Y, et al. Bone-marrow-homing lipid nanoparticles for genome editing in diseased and malignant haematopoietic stem cells. Nat Nanotechnol. 2024;19(9):1409–17. 38783058 10.1038/s41565-024-01680-8 PMC11757007

[375] Niu D, Wu Y, Lian J. Circular RNA vaccine in disease prevention and treatment. Signal Transduct Target Ther. 2023;8(1):341. 37691066 10.1038/s41392-023-01561-x PMC10493228

[376] Zhu Y, Zhu L, Wang X, Jin H. RNA-based therapeutics: an overview and prospectus. Cell Death Dis. 2022;13(7):644. 35871216 10.1038/s41419-022-05075-2 PMC9308039

[377] Zamecnik PC, Stephenson ML. Inhibition of rous sarcoma virus replication and cell transformation by a specific oligodeoxynucleotide. Proc Natl Acad Sci U S A. 1978;75(1):280–4. 75545 10.1073/pnas.75.1.280 PMC411230

[378] Paunovska K, Loughrey D, Dahlman JE. Drug delivery systems for RNA therapeutics. Nat Rev Genet. 2022;23(5):265–80. 34983972 10.1038/s41576-021-00439-4 PMC8724758

[379] Zhang Q, Pan J, Xiong D, Wang Y, Miller MS, Sei S, et al. Pulmonary aerosol delivery of LET-7B microrna confers a striking inhibitory effect on lung carcinogenesis through targeting the tumor immune microenvironment. Adv Sci. 2021;8(17):e2100629. 10.1002/advs.202100629 PMC8425922 34236760

[380] El Fatimy R, Subramanian S, Uhlmann EJ, Krichevsky AM. Genome editing reveals glioblastoma addiction to microRNA-10B. Mol Ther. 2017;25(2):368–78. 28153089 10.1016/j.ymthe.2016.11.004 PMC5368404

[381] Zou Y, Sun X, Wang Y, Yan C, Liu Y, Li J, et al. Single siRNA nanocapsules for effective siRNA brain delivery and glioblastoma treatment. Adv Mater. 2023;35(16):e2300777. 37078222 10.1002/adma.202300777

[382] Cohen ZR, Ramishetti S, Peshes-Yaloz N, Goldsmith M, Wohl A, Zibly Z, et al. Localized RNAi therapeutics of chemoresistant grade IV glioma using hyaluronan-grafted lipid-based nanoparticles. ACS Nano. 2015;9(2):1581–91. 25558928 10.1021/nn506248s

[383] Wang Z, Tang XL, Zhao MJ, Zhang YD, Xiao Y, Liu YY, et al. Biomimetic hypoxia-triggered RNAi nanomedicine for synergistically mediating chemo/radiotherapy of glioblastoma. J Nanobiotechnology. 2023;21(1):210. 37408007 10.1186/s12951-023-01960-w PMC10324258

[384] Fan HY, Liang XH, Tang YL. Neuroscience in peripheral cancers: tumors hijacking nerves and neuroimmune crosstalk. MedComm. 2024;5(11):e784. 39492832 10.1002/mco2.784 PMC11527832

[385] Drost J, Clevers H. Organoids in cancer research. Nat Rev Cancer. 2018;18(7):407–18. 29692415 10.1038/s41568-018-0007-6

[386] Lu T, Wang M, Zhou W, Ni Q, Yue Y, Wang W, et al. Decoding transcriptional identity in developing human sensory neurons and organoid modeling. Cell. 2024;187(26):7374–93.e28. 39536745 10.1016/j.cell.2024.10.023

[387] Vaes N, Schonkeren SL, Rademakers G, Holland AM, Koch A, Gijbels MJ, et al. Loss of enteric neuronal NDRG4 promotes colorectal cancer via increased release of NID1 and FBLN2. EMBO Rep. 2021;22(6):e51913. 33890711 10.15252/embr.202051913 PMC8183412

[388] Kim J, Kim R, Lee W, Kim GH, Jeon S, Lee YJ, et al. Assembly of glioblastoma tumoroids and cerebral organoids: a 3d in vitro model for tumor cell invasion. Mol Oncol. 2024. 10.1002/1878-0261.13740. 39473365 10.1002/1878-0261.13740 PMC11887666

[389] Ma X, Wang Q, Li G, Li H, Xu S, Pang D. Cancer organoids: a platform in basic and translational research. Genes Dis. 2024;11(2):614–32. 37692477 10.1016/j.gendis.2023.02.052 PMC10491878

[390] Marx V. Closing in on cancer heterogeneity with organoids. Nat Methods. 2024;21(4):551–4. 38528185 10.1038/s41592-024-02231-8

[391] Wefel JS, Kayl AE, Meyers CA. Neuropsychological dysfunction associated with cancer and cancer therapies: a conceptual review of an emerging target. Br J Cancer. 2004;90(9):1691–6. 15150608 10.1038/sj.bjc.6601772 PMC2410277

[392] Staff NP, Grisold A, Grisold W, Windebank AJ. Chemotherapy-induced peripheral neuropathy: a current review. Ann Neurol. 2017;81(6):772–81. 28486769 10.1002/ana.24951 PMC5656281

[393] Bannwarth B, Kostine M. Nerve growth factor antagonists: is the future of monoclonal antibodies becoming clearer?. Drugs. 2017;77(13):1377–87. 28660479 10.1007/s40265-017-0781-6

[394] Gibson EM, Nagaraja S, Ocampo A, Tam LT, Wood LS, Pallegar PN, et al. Methotrexate chemotherapy induces persistent tri-glial dysregulation that underlies chemotherapy-related cognitive impairment. Cell. 2019;176(1–2):43–55.e13. 30528430 10.1016/j.cell.2018.10.049 PMC6329664

[395] Pease-Raissi SE, Pazyra-Murphy MF, Li Y, Wachter F, Fukuda Y, Fenstermacher SJ, et al. Paclitaxel reduces axonal Bclw to initiate IP_3_ R1-dependent axon degeneration. Neuron. 2017;96(2):373–86.e6. 29024661 10.1016/j.neuron.2017.09.034 PMC5680044

[396] Sarfati D, Koczwara B, Jackson C. The impact of comorbidity on cancer and its treatment. CA. 2016;66(4):337–50. 26891458 10.3322/caac.21342

[397] Huang JW, Cao CA, Zheng WH, Jia CR, Liu X, Gao SQ, et al. The mechanism of cancer-depression comorbidity. Neuroscience. 2024;556:25–30. 39094819 10.1016/j.neuroscience.2024.07.040

[398] Wang L, Zhang W, He X, Zha H, editors. Personalized prescription for comorbidity. In: Database Systems for Advanced Applications: 23rd International Conference, DASFAA 2018, Gold Coast, QLD, Australia, May 21–24, 2018, Proceedings, Part II 23; 2018: Springer.

[399] Stairmand J, Signal L, Sarfati D, Jackson C, Batten L, Holdaway M, et al. Consideration of comorbidity in treatment decision making in multidisciplinary cancer team meetings: a systematic review. Ann Oncol. 2015;26(7):1325–32. 25605751 10.1093/annonc/mdv025

[400] Ojima K, Kakegawa W, Yamasaki T, Miura Y, Itoh M, Michibata Y, et al. Coordination chemogenetics for activation of GPCR-type glutamate receptors in brain tissue. Nat Commun. 2022;13(1):3167. 35710788 10.1038/s41467-022-30828-0 PMC9203742

[401] Costa PAC, Silva WN, Prazeres P, Picoli CC, Guardia GDA, Costa AC, et al. Chemogenetic modulation of sensory neurons reveals their regulating role in melanoma progression. Acta Neuropathol Commun. 2021;9(1):183. 34784974 10.1186/s40478-021-01273-9 PMC8594104

[402] Ben-Shaanan TL, Azulay-Debby H, Dubovik T, Starosvetsky E, Korin B, Schiller M, et al. Activation of the reward system boosts innate and adaptive immunity. Nat Med. 2016;22(8):940–4. 27376577 10.1038/nm.4133

[403] Gomez JL, Bonaventura J, Lesniak W, Mathews WB, Sysa-Shah P, Rodriguez LA, et al. Chemogenetics revealed: dreadd occupancy and activation via converted clozapine. Science. 2017;357(6350):503–7. 28774929 10.1126/science.aan2475 PMC7309169

[404] Phillips JA, Hutchings C, Djamgoz MBA. Clinical potential of nerve input to tumors: a bioelectricity perspective. Bioelectricity. 2021;3(1):14–26. 34476375 10.1089/bioe.2020.0051 PMC8390776

[405] Armbruster BN, Li X, Pausch MH, Herlitze S, Roth BL. Evolving the lock to fit the key to create a family of G protein-coupled receptors potently activated by an inert ligand. Proc Natl Acad Sci U S A. 2007;104(12):5163–8. 17360345 10.1073/pnas.0700293104 PMC1829280

[406] Oesterle TS, Bormann NL, Al-Soleiti M, Kung S, Singh B, McGinnis MT, et al. Invasive and non-invasive neuromodulation for the treatment of substance use disorders: a review of reviews. Brain Sci. 2025;15(7):723. 40722314 10.3390/brainsci15070723 PMC12294003

[407] Soltani Dehnavi S, Eivazi Zadeh Z, Harvey AR, Voelcker NH, Parish CL, Williams RJ, et al. Changing fate: reprogramming cells via engineered nanoscale delivery materials. Adv Mater. 2022;34(33):e2108757. 35396884 10.1002/adma.202108757

[408] Couch Y, Buzàs EI, Di Vizio D, Gho YS, Harrison P, Hill AF, et al. A brief history of nearly everything - the rise and rise of extracellular vesicles. J Extracell Vesicles. 2021;10(14):e12144. 34919343 10.1002/jev2.12144 PMC8681215

[409] Luo W, Dai Y, Chen Z, Yue X, Andrade-Powell KC, Chang J. Spatial and temporal tracking of cardiac exosomes in mouse using a nano-luciferase-CD63 fusion protein. Commun Biol. 2020;3(1):114. 32157172 10.1038/s42003-020-0830-7 PMC7064570

[410] Beetler DJ, Di Florio DN, Bruno KA, Ikezu T, March KL, Cooper LT, et al. Extracellular vesicles as personalized medicine. Mol Aspects Med. 2023;91:101155. 36456416 10.1016/j.mam.2022.101155 PMC10073244

[411] Arsić A, Stajković N, Spiegel R, Nikić-Spiegel I. Effect of vectashield-induced fluorescence quenching on conventional and super-resolution microscopy. Sci Rep. 2020;10(1):6441. 32296095 10.1038/s41598-020-63418-5 PMC7160131

[412] Zhu B, Yin H, Zhang D, Zhang M, Chao X, Scimeca L, et al. Synthetic biology approaches for improving the specificity and efficacy of cancer immunotherapy. Cell Mol Immunol. 2024;21(5):436–47. 38605087 10.1038/s41423-024-01153-x PMC11061174

[413] Benzaquen D, Lawrence YR, Taussky D, Zwahlen D, Oehler C, Champion A. The crosstalk between nerves and cancer-a poorly understood phenomenon and new possibilities. Cancers (Basel). 2024;16(10):1875. 38791953 10.3390/cancers16101875 PMC11120349

